# Towards Enhanced Tunability of Aqueous Biphasic Systems: Furthering the Grasp of Fluorinated Ionic Liquids in the Purification of Proteins

**DOI:** 10.3390/ijms25115766

**Published:** 2024-05-25

**Authors:** Sara F. Carvalho, Margarida H. Custódio, Ana B. Pereiro, João M. M. Araújo

**Affiliations:** LAQV, REQUIMTE, Department of Chemistry, NOVA School of Science and Technology, NOVA University Lisbon, 2829-516 Caparica, Portugal; sfe.carvalho@campus.fct.unl.pt (S.F.C.); m.custodio@campus.fct.unl.pt (M.H.C.); anab@fct.unl.pt (A.B.P.)

**Keywords:** lysozyme, BSA, aqueous biphasic systems, biocompatible fluorinated ionic liquids, selective partition

## Abstract

This work unfolds functionalized ABSs composed of FILs ([C_2_C_1_Im][C_4_F_9_SO_3_] and [$N_{1112(\mathrm{OH})}$][C_4_F_9_SO_3_]), mere fluoro-containing ILs ([C_2_C_1_Im][CF_3_SO_3_] and [C_4_C_1_Im][CF_3_SO_3_]), known globular protein stabilizers (sucrose and [$N_{1112(\mathrm{OH})}$][C_4_F_9_SO_3_]), low-molecular-weight carbohydrate (glucose), and even high-charge density salt (K_3_PO_4_). The ternary phase diagrams were determined, stressing that FILs highly increased the ability for ABS formation. The functionalized ABSs (FILs vs. mere fluoro-containing ILs) were used to extract lysozyme (Lys). The ABSs’ biphasic regions were screened in terms of protein biocompatibility, analyzing the impact of ABS phase-forming components in Lys by UV-VIS spectrophotometry, CD spectroscopy, fluorescence spectroscopy, DSC, and enzyme assay. Lys partition behavior was characterized in terms of extraction efficiency (% EE). The structure, stability, and function of Lys were maintained or improved throughout the extraction step, as evaluated by CD spectroscopy, DSC, enzyme assay, and SDS-PAGE. Overall, FIL-based ABSs are more versatile and amenable to being tuned by the adequate choice of the phase-forming components and selecting the enriched phase. Binding studies between Lys and ABS phase-forming components were attained by MST, demonstrating the strong interaction between Lys and FILs aggregates. Two of the FIL-based ABSs (30 %wt [C_2_C_1_Im][C_4_F_9_SO_3_] + 2 %wt K_3_PO_4_ and 30 %wt [C_2_C_1_Im][C_4_F_9_SO_3_] + 25 %wt sucrose) allowed the simultaneous purification of Lys and BSA in a single ABS extraction step with high yield (extraction efficiency up to 100%) for both proteins. The purity of both recovered proteins was validated by SDS-PAGE analysis. Even with a high-charge density salt, the FIL-based ABSs developed in this work seem more amenable to be tuned. Lys and BSA were purified through selective partition to opposite phases in a single FIL-based ABS extraction step. FIL-based ABSs are proposed as an improved extraction step for proteins, based on their biocompatibility, customizable properties, and selectivity.

## 1. Introduction

Aqueous biphasic systems (ABSs), also known as aqueous two-phase systems (ATPSs), were originally proposed by Albertsson [1]. These liquid–liquid systems consist of two liquid phases that exist in equilibrium above specific concentrations of at least two different water-soluble compounds in water. These systems can include a variety of components such as polymers, salts, alcohols, carbohydrates, or ionic liquids (ILs) [2,3]. ABSs have numerous advantages in biotechnological applications [4,5], such as scaling up feasibility, ease of continuous process, lower interfacial tension, and, one of the most relevant, their biocompatibility, mostly due to the high water content of both phases, providing a biocompatible and non-denaturing environment for cells, proteins, and other biomolecules [2,6,7,8]. Additionally, ABSs easily allow integration into various conventional downstream processes (clarification, concentration, or partial purification) as a single-step operation [9], with low investment costs and low environmental toxicity risks compared to traditional biomolecule purification methods [10,11]. ABSs were implemented for the separation, recovery, and purification of nucleic acids, DNA, cells, membrane viruses, enzymes, proteins, and other value-added biomolecules [2,3,12]. Two transversal drawbacks of polymer/polymer, polymer/salt, and alcohol/salt ABSs are that they exhibit a narrow hydrophilic–hydrophobic range and a small range of polarities between the two phases [2,13]. To address these limitations and enhance the protein purification performance of ABS, several strategies were implemented, namely, the functionalization of polyethylene glycols [14,15], the use of ILs as adjuvants in polymer/salt ABSs [3,16], and the development of ionic-liquid-based ABSs [2,3,13,17,18,19]. Nevertheless, the paramount feature afforded by IL-based ABSs is certainly their remarkable performance in extractions and selectivity for a wide variety of biomolecules, simply attained by judicious selection of ABS phase-forming components and their compositions [2,3,19].

Ionic liquids are salts that are liquid at low temperatures, in contrast to common electrolytes, due to asymmetry and delocalized electrical charge distribution of the constituent ions, which prevent crystallization. The physical and chemical properties of ILs, properly manipulated for specific applications by the judicious selection of cations and anions, are transferable to aqueous solutions of ILs. In this framework, IL-based ABSs have demonstrated a sizable applicability through adequate control of the polarities and affinities of the phases [2,18,19,20]. Additionally, the tunability of ILs, at least ideally, allows them to cover the full hydrophilicity–hydrophobicity range, and selective extractions can be readily envisioned. In downstream processes, the toxicity of the substances involved must be considered, as well as the biocompatibility and efficiency of protein stability. For ionic-liquid-based processes, these can be achieved by the development of functionalized ILs, such as the fluorinated ionic liquids (FILs) developed in our study. The development of bioprivileged and functionalized FILs was attained by increasing the perfluoroalkylsulfonate anion chain ([C_4_F_9_SO_3_]^−^ or greater) [20,21,22,23,24,25,26,27,28]. These FILs have total miscibility in water, forming diverse aggregated structures, from spherical to lamellar micelles, depending on the concentration in aqueous solution [21]. This rich-aggregation behavior stimulates the networking of water aggregates, which is expanded by means of increasing the fluorinated chain, since the nonpolar part drives the water molecules into the polar domain of the IL [20]. Further, the impact of water addition upon the ionic liquid’s H-bond acceptance ability is reduced in these FILs, which is a key factor in the development of functionalized materials for dissolution or extraction processes of biomolecules [20]. Additionally, the [C_4_F_9_SO_3_]-based FILs are non-toxic and biocompatible in aquatic species with different levels of biological organization (Daphnia magna, Lemna minor, and Vibrio fischeri) and in four human cell lines (Caco-2, EA.hy926, HaCaT, and HepG2) [22,23]. Additionally, the effect of [C_4_F_9_SO_3_]-based FILs on BSA, lysozyme, IFN-α2b, and human phenylalanine hydroxylase as model proteins was evaluated, establishing these FILs as biocompatible and apt potential biomaterials for drug delivery systems [24,25,26,27,28]. Moreover, the functionalization of ABSs was attained via the development of ABSs formed by [C_4_F_9_SO_3_]-based FILs. Firstly, implementing a variety of monosaccharides, disaccharides, and polyols, know as being less effective in inducing liquid–liquid demixing, as selective and efficient extractive platforms of food colorants [3]. In a concurrent work [19], a set of bioprivileged FIL-based ABSs, developed in the present work, were engineered for the selective purification of interferon alpha-2b (IFN-α2b) and serum albumin (bovine serum albumin, BSA, used as serum albumin protein model) in a single ABS extraction step. IFN-α2b (the high-value-added compound) is an essential cytokine widely used in the treatment of hepatitis B and C, hairy cell leukemia, melanoma, and non-Hodgkin’s lymphoma [29], and serum albumin is the most abundant plasma protein with numerous physiological functions (e.g., lipid metabolism, metal ion transport, binding small therapeutic molecule drugs, and maintaining colloid osmotic pressure of the blood) [30,31,32].

Proteins are intricate biomolecules that integrate numerous vital processes that rely on the preservation of their secondary structural elements to remain active. These elements are intricately shaped by a delicate equilibrium between distinct interactions, namely hydrogen bonds, disulfide bridges, ionic interactions, and hydrophobic interactions [24,25,26,27,28,33]. Consequently, native globular proteins are densely packed to avoid non-specific aggregation [34]. The destabilization of the structure of proteins can expose buried hydrophobic domains, potentially leading to non-specific interactions [35]. The intrinsic instability of proteins, structural and chemical, associated with short half-lives when subjected to physical and chemical stress, limits the extraction and purification steps of proteins [19,28,36]. Though proteins are stabilized by the equilibrium between intramolecular interactions and interactions with the solvent environment, the employment of biocompatible fluorinated ionic liquids (as previously detailed, as well as biocompatible ABS phase-forming components) can provide an alternative strategy to preserve their stability [19,24,25,26,27,28,37]. Lysozyme (Lys; muramidase, EC 3.2.1.17), an extensively studied protein characterized as a valuable protein model [24,25,38], was used herein as a protein model as well. Lys is a natural 14 kDa globular protein found in mucous secretions (tears, saliva, and mucus) and tissues of animals and plants, where it plays a crucial role in innate defense mechanisms, protecting against bacteria, viruses, and fungi. Given its enzymatic activity, through hydrolysis of the β-1,4-glycosidic bonds in the peptidoglycan of the Gram positive bacterial cell wall, it can serve in plenty of applications, from food preservatives to therapeutic proteins against bacterial infections or immune response control [39]. This protein additionally contains intrinsic fluorescence, provided by tryptophan residues 62 and 108, which may be used to conduct an array of studies, including fluorescence spectroscopy [40].

In this work, it is demonstrated that the functionalization of ionic liquids, conventional ILs vs. mere fluoro-containing ILs vs. fluorinated ILs, is transferable to functionalized ABSs, allowing the formation of ABSs with more biocompatible phase-forming components, such as low-molecular-weight carbohydrates (glucose and sucrose) or choline dihydrogen phosphate ([$N_{1112(\mathrm{OH})}$][H_2_PO_4_], a known globular protein stabilizer as sucrose). The impact of the proposed functionalization is assessed by the determination of forty-three phase diagrams (solubility curves) at 25 °C and atmospheric pressure (plus ten mixtures whose liquid–liquid demixing is not verified). To evaluate the potential of the biocompatible FIL-based ABSs as extractive or purification platforms for proteins, twelve of the functionalized ABSs developed (FIls vs. mere fluoro-containing ILs) were tested in the partition of lysozyme—used as a protein model. Prior to partition studies, the twelve biphasic regions were screened in terms of protein biocompatibility. Lysozymes, in aqueous solution with the ABS phase-forming components, were characterized by UV-VIS spectrophotometry (transmittance and absorption spectra), circular dichroism (CD) spectroscopy, fluorescence spectroscopy, differential scanning calorimetry (DSC), and enzyme assay (EA). Lysozyme partition behavior in the twelve biphasic systems is characterized in terms of extraction efficiency (% EE), and the partition Lys is characterized by enzyme assay (EA), differential scanning calorimetry (DSC), circular dichroism (CD) spectroscopy, and SDS-polyacrylamide gel electrophoresis (SDS-PAGE). Binding studies of lysozyme with the ABS phase-forming components were assessed by microscale thermophoresis (MST) to unveil the interactions ruling partition. Additionally, the functionalized biocompatible FIL-based ABSs allowed the simultaneous purification of Lys and BSA (two globular proteins) in a single ABS extraction step with high yield (% EE) and purity (assessed by SDS-PAGE analysis).

## 2. Results and Discussion

### 2.1. Phase Diagrams for Functionalized Aqueous Biphasic Systems

**Figure 1:**
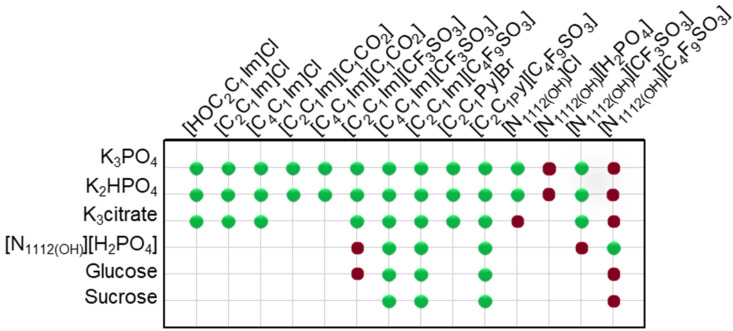
Mapping the combinations of conventional ILs, mere fluoro-containing ILs, and fluorinated ILs with inorganic salts, low-molecular-weight carbohydrates, and a known globular protein stabilizer ([$N_{1112(\mathrm{OH})}$][H_2_PO_4_]) for ABS formation at 25 °C. The colors refer to the demixing: green for mixtures which undergo demixing and form ABSs, red for mixtures where no macroscopic demixing was observed.

In this work, novel systems combining biocompatible [C_4_F_9_SO_3_]-based FILs ([C_2_C_1_Im][C_4_F_9_SO_3_], [$N_{1112(\mathrm{OH})}$][C_4_F_9_SO_3_] and [C_2_C_1_Py][C_4_F_9_SO_3_]) and mere fluoro-containing ILs ([C_2_C_1_Im][CF_3_SO_3_], [C_4_C_1_Im][CF_3_SO_3_] and [$N_{1112(\mathrm{OH})}$][CF_3_SO_3_]) with inorganic salts (K_3_PO_4_, K_2_HPO_4_, and K_3_citrate), low-molecular-weight carbohydrates (glucose and sucrose), and a known globular protein stabilizer ([$N_{1112(\mathrm{OH})}$][H_2_PO_4_]) were investigated concerning their ability to form aqueous biphasic systems and to demonstrate that the FIL-based ABSs are more versatile and amenable to be tuned, allowing to replace high-charge density salts with more benign phase-forming components. In this framework, the mapping of combinations to test liquid–liquid demixing was extended to conventional ILs (e.g., [C_2_C_1_Im]Cl, [C_4_C_1_Im]Cl, [C_2_C_1_Im][C_1_CO_2_] or [C_4_C_1_Im][C_1_CO_2_]), as illustrated in Figure 1. The chemical structures, acronym, and designation of all the studied ionic liquids, including conventional ILs, mere fluoro-containing ILs, and fluorinated ILs, are listed in Table S1 of Supplementary Materials. Since different ionic liquids (FILs, mere fluoro-containing ILs, and conventional ILs), inorganic/organic salts, and carbohydrates were used, the influence of the structure of both components on the solubility curves (phase diagrams) could be evaluated. Accordingly, the binodal curves and respective tie-lines were determined for each system at 25 °C and atmospheric pressure. The impact of all the ABS phase-forming components on the ternary phase diagrams will be discussed. All the ternary phase diagrams expressed in %wt for the ABSs listed in Figure 1 are presented in Tables S2–S21 and plotted in Figures S1–S6 of Supplementary Materials. A few systems have been previously reported in the literature and are in good agreement with our experimental data [2,18,36,41,42,43]. Further analysis on the functionalization of ABSs was only attained for the twelve biphasic systems, addressing the functionalization of FILs vs. mere fluoro-containing ILs, listed in Table 1. In these studied systems, the bottom phase is the ionic-liquid-rich phase (enriched in FILs or mere fluoro-containing ILs), and the top phase is the non-ionic-liquid-rich phase (enriched in inorganic salts, carbohydrates, or [$N_{1112(\mathrm{OH})}$][H_2_PO_4_]). The binodal curves obtained for the studied twelve biphasic systems (Table 1) were fitted using Equation (1) and the respective *A*, *B*, and *C* parameters were estimated by least-squares regression [44]. This fitting procedure may provide experimentally unavailable data for the ABSs, if needed. The detailed compositions of the coexisting phases, as well as other relevant phase properties, are summarized in Table 1. The water amount (%wt H_2_O), phase volume, and pH of the coexisting phases were experimentally measured as described in Section 3. The ionic liquid ([C_2_C_1_Im][C_4_F_9_SO_3_], [$N_{1112(\mathrm{OH})}$][C_4_F_9_SO_3_], [C_4_C_1_Im][CF_3_SO_3_] and [C_2_C_1_Im][CF_3_SO_3_]) composition (%wt IL) and non-ionic liquid ([$N_{1112(\mathrm{OH})}$][H_2_PO_4_], K_3_PO_4_, glucose, and sucrose) composition (%wt non-IL) were determined by the fitting of Equation (1) (Section 3). The tie-line length and tie-line slope for the twelve biphasic systems are listed in Table S22 of Supplementary Materials.

**Figure 2:**
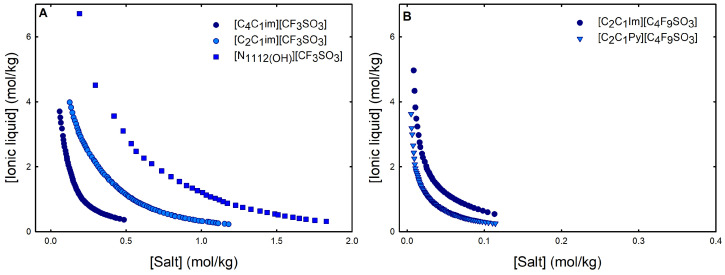
Ternary phase diagrams in molality for (**A**) mere fluoro-containing ILs ([C_2_C_1_Im][CF_3_SO_3_], [C_4_C_1_Im][CF_3_SO_3_] and [$N_{1112(\mathrm{OH})}$][CF_3_SO_3_]) and (**B**) fluorinated ILs ([C_2_C_1_Im][C_4_F_9_SO_3_] and [C_2_C_1_Py][C_4_F_9_SO_3_]) combined with K_3_PO_4_ at 25 °C and atmospheric pressure.

**Figure 3:**
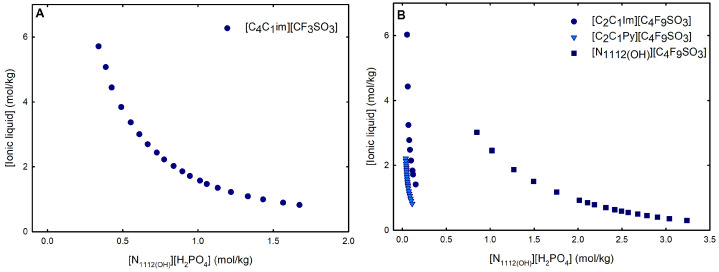
Ternary phase diagrams in molality for (**A**) mere fluoro-containing IL ([C_4_C_1_Im][CF_3_SO_3_]) and (**B**) fluorinated ILs ([C_2_C_1_Im][C_4_F_9_SO_3_] [C_2_C_1_Py][C_4_F_9_SO_3_] and [$N_{1112(\mathrm{OH})}$][C_4_F_9_SO_3_]) combined with K_3_PO_4_ at 25 °C and atmospheric pressure.

**Figure 4:**
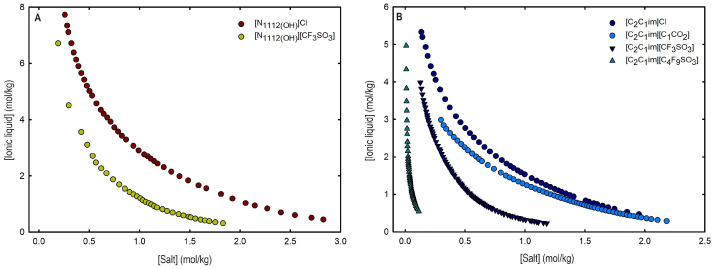
Ternary phase diagrams in molality for (**A**) cholinium-based ILs ([$N_{1112(\mathrm{OH})}$]Cl and [$N_{1112(\mathrm{OH})}$][CF_3_SO_3_]) and (**B**) imidazolium-based ILs ([C_2_C_1_Im]Cl, [C_2_C_1_Im][C_1_CO_2_], [C_2_C_1_Im][CF_3_SO_3_] and [C_2_C_1_Im][C_4_F_9_SO_3_]) combined with K_3_PO_4_ at 25 °C and atmospheric pressure.

Ternary phase diagrams depict the minimum concentration of ABS phase-forming compounds necessary for liquid–liquid demixing. Simply said, phase separation occurs in mixtures with compositions above the binodal curve (known as the biphasic region), whereas the monophasic region exists below the binodal curve. When selecting a suitable system, the closer the binodal curve is to the origin axis the binodal curve is, the greater the system’s ability to induce phase separation. Figure 2 depicts the ternary phase diagrams in molality for [CF_3_SO_3_]-based mere fluoro-containing ILs (Figure 2A) and [C_4_F_9_SO_3_]-based FILs (Figure 2B) combined with K_3_PO_4_, in order to ascertain the cation impact on the ABS phase formation. [C_4_C_1_Im][CF_3_SO_3_] promotes ABSs more effectively than [C_2_C_1_Im][CF_3_SO_3_], whereas [$N_{1112(\mathrm{OH})}$][CF_3_SO_3_] has the least potential. For [C_4_F_9_SO_3_]-based FILs, liquid–liquid demixing is not verified with [$N_{1112(\mathrm{OH})}$][C_4_F_9_SO_3_] and the following order in demixing is verified: [C_2_C_1_Py]^+^ > [C_2_C_1_Im]^+^. For [$N_{1112(\mathrm{OH})}$][H_2_PO_4_]-based ABSs, liquid–liquid demixing is not verified for [C_2_C_1_Im][CF_3_SO_3_] and [$N_{1112(\mathrm{OH})}$][CF_3_SO_3_]; only for the cation with a longer alkyl chain length [C_4_C_1_Im][CF_3_SO_3_] is demixing verified (Figure 1). For [C_2_C_1_Im][C_4_F_9_SO_3_] liquid–liquid demixing is verified (Figure 3). For FIL-based ABSs combined with [$N_{1112(\mathrm{OH})}$][H_2_PO_4_], [C_2_C_1_Py][C_4_F_9_SO_3_] performs slightly better than [C_2_C_1_Im][C_4_F_9_SO_3_] and [$N_{1112(OH)}$][C_4_F_9_SO_3_] is the least effective with the smallest biphasic region. Both Figure 2 and Figure 3 allow to rank the cations used in the ABS functionalization proposed in this work (fluorinated ILs vs. mere fluoro-containing ILs) in decreasing the order of ABS-forming ability, [C_2_C_1_Py]^+^ >[C_4_C_1_Im]^+^ > [C_2_C_1_Im]^+^ > [$N_{1112(OH)}$]^+^. The FIL-based functionalized ABSs allows liquid–liquid demixing with a known globular protein stabilizer ([$N_{1112(\mathrm{OH})}$][H_2_PO_4_]) with an ionic liquid with short cation alkyl chain length ([C_2_C_1_Im][C_4_F_9_SO_3_] vs. [C_2_C_1_Im][CF_3_SO_3_]) and a cholinium-based ionic liquid ([$N_{1112(\mathrm{OH})}$][C_4_F_9_SO_3_] vs. [$N_{1112(\mathrm{OH})}$][CF_3_SO_3_]), with all the advantages of shorter cation alkyl chain length ILs and cholinium-based ILs for protein applications. It is relevant to highlight that [$N_{1112(\mathrm{OH})}$][C_4_F_9_SO_3_] only present liquid–liquid demixing with [$N_{1112(\mathrm{OH})}$][H_2_PO_4_] (Figure 1). Even with K_3_PO_4_, one of the strongest kosmotropic salts available (producing intense salting-out effects) [45], liquid–liquid demixing is not verified. The other studied FILs ([C_2_C_1_Im][C_4_F_9_SO_3_] and [C_2_C_1_Py][C_4_F_9_SO_3_]) allow liquid–liquid demixing with all second ABS phase-forming components (Figure 1), inorganic salts (K_3_PO_4_, K_2_HPO_4_ and K_3_citrate), low-molecular-weight carbohydrates (glucose and sucrose), and a known globular protein stabilizer ([$N_{1112(\mathrm{OH})}$][H_2_PO_4_]). Figure 4 illustrates the functionalization trend of ABSs addressed in this work, conventional ILs vs. mere fluoro-containing ILs vs. fluorinated ILs, through anion functionalization fixing the IL cation. For [$N_{1112(\mathrm{OH})}$]-based ILs, liquid–liquid demixing was not verified with [$N_{1112(\mathrm{OH})}$][C_4_F_9_SO_3_] and [$N_{1112(\mathrm{OH})}$][H_2_PO_4_] (Figure 1). The systems with K_3_PO_4_ were selected to assess the functionalization due to the higher number of positive liquid–liquid demixings. As shown in Figure 4A, the biphasic region of a conventional [$N_{1112(\mathrm{OH})}$]-based IL can be improved by changing the constituent anion from a halogen (Cl^−^) to [CF_3_SO_3_]^−^ (conventional ILs vs. mere fluoro-containing ILs). For [C_2_C_1_Im]-based ILs, liquid–liquid demixing is verified with conventional ILs ([C_2_C_1_Im]Cl and [C_2_C_1_Im][C_1_CO_2_]), mere fluoro-containing ILs ([C_2_C_1_Im][CF_3_SO_3_]), and fluorinated ILs ([C_2_C_1_Im][C_4_F_9_SO_3_]), as depicted in Figure 4B. The functionalization trend allows for an increase in the system’s ability to induce phase separation, reducing the amount of ABS phase-forming components for liquid–liquid demixing. The contributing effect of the studied IL functionalization, conventional ILs vs. mere fluoro-containing ILs vs. fluorinated ILs, to form ABSs follows the decreasing order (in molality units): [C_4_F_9_SO_3_]^−^ > [CF_3_SO_3_]^−^ > [C_1_CO_2_]^−^ > Cl^−^.

**Figure 5:**
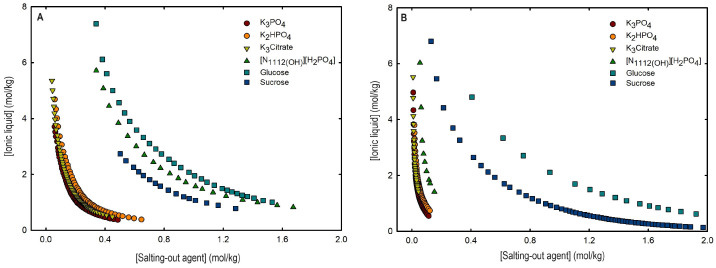
Ternary phase diagrams in molality for (**A**) [C_4_C_1_Im][CF_3_SO_3_] (mere fluoro-containing IL) and (**B**) [C_2_C_1_Im][C_4_F_9_SO_3_] (FIL) combined with inorganic salts (K_3_PO_4_, K_2_HPO_4_, and K_3_Citrate), a know globular protein stabilizer ([$N_{1112(\mathrm{OH})}$][H_2_PO_4_]) and low-molecular-weight carbohydrates (glucose and sucrose) at 25 °C and atmospheric pressure.

An overall analysis of the core ABS functionalization addressed in this work, fluorinated ILs ([C_4_F_9_SO_3_]-based ILs) vs. mere fluoro-containing ILs ([CF_3_SO_3_]-based ILs), in terms of solubility curves (phase diagrams), is detailed in Figure 5, comparing [C_2_C_1_Im][C_4_F_9_SO_3_] with [C_4_C_1_Im][CF_3_SO_3_] combined with all the second ABS phase-forming components. The mere fluoro-containing [C_2_C_1_Im][CF_3_SO_3_] does not undergo liquid–liquid demixing with low-molecular-weight carbohydrates and [$N_{1112(\mathrm{OH})}$][H_2_PO_4_] demixing is only verified with the studied inorganic salts (Figure 1). Accordingly, we have to increase the cation alkyl chain length ([C_4_C_1_Im][CF_3_SO_3_] vs. [C_2_C_1_Im][CF_3_SO_3_]) to undergo demixing with glucose, sucrose, and [$N_{1112(\mathrm{OH})}$][H_2_PO_4_], with all the disadvantages of longer cation alkyl chain length ILs for protein applications, to address the ABS functionalization ([C_4_F_9_SO_3_]^−^ vs. [CF_3_SO_3_]^−^). The ternary phase diagrams are depicted in Figure 5, demonstrating that the FIL-based ABSs have the highest ability to induce phase separation (solubility curves closer to the origin axis), allowing to reduce the amount of ABS phase-forming components for liquid–liquid demixing. In particular, the solubility curve for [$N_{1112(\mathrm{OH})}$][H_2_PO_4_] (a known globular protein stabilizer) with [C_2_C_1_Im][C_4_F_9_SO_3_] is almost superimposed with the solubility curve with K_3_PO_4_ (one of the strongest kosmotropic salts available, producing intense salting-out effects) as depicted in Figure 5B. A completely different behavior is verified for the phase diagram of [C_4_C_1_Im][CF_3_SO_3_] + [$N_{1112(\mathrm{OH})}$][H_2_PO_4_] (Figure 5A). A general comparison of the phase diagrams for all the ionic liquids combined with K_3_PO_4_ (kosmotropic salt with the higher number of positive liquid–liquid demixing; Figure 5) is depicted in Figure S7 of Supplementary Materials, demonstrating the impact of the global functionalization trend of ABSs addressed in this work, conventional ILs vs. mere fluoro-containing ILs vs. fluorinated ILs, in the ionic liquids’ ability to form ABSs. The decreasing order is as follows (in molality units), [C_2_C_1_Py][C_4_F_9_SO_3_] ≥ [C_2_C_1_Im][C_4_F_9_SO_3_] > [C_4_C_1_Im][CF_3_SO_3_] > [C_2_C_1_Im][CF_3_SO_3_] > [C_2_C_1_Py]Br ≥ [C_4_C_1_Im][C_1_CO_2_] ≈ [C_4_C_1_Im]Cl ≈ [C_2_C_1_Im][C_1_CO_2_] ≥ [$N_{1112(\mathrm{OH})}$][CF_3_SO_3_] ≥ [C_2_C_1_Im]Cl > [HOC_2_C_1_Im]Cl > [$N_{1112(\mathrm{OH})}$]Cl. Once again, the specified trend indicates that the FIL-based ABSs have the highest ability to induce phase separation (solubility curves closer to the origin axis), reducing the amount of ABS phase-forming components for liquid–liquid demixing.

From the inspection of the solubility curves obtained with the FILs [C_2_C_1_Im][C_4_F_9_SO_3_], [C_2_C_1_Py][C_4_F_9_SO_3_] and [$N_{1112(\mathrm{OH})}$][C_4_F_9_SO_3_], depicted in Figure 2, Figure 3, Figure 4 and Figure 5 and Figure S7 of Supplementary Materials, and based on the chemical structures of the ionic liquids (conventional ILs, mere fluoro-containing ILs, and fluorinated ILs) that were not able to promote liquid–liquid demixing (Figure 1), some major perspectives can be ascertained. The ability for liquid–liquid demixing is higher for the cation [C_2_C_1_Py]^+^, a six-carbon ring, than for the cation [C_2_C_1_Im]^+^, a five-carbon ring. This is in agreement with previous works that have shown that ILs having cations containing six-carbon rings (pyridinium and piperidinium) formed ABSs more easily than ILs containing five-carbon rings (imidadolium and pyrrolidinium) [3,46]. Both studies demonstrated that aromaticity does not play a significant role in promoting two-phase formation, which is strongly correlated with the cation size. Since [C_2_C_1_Im][C_4_F_9_SO_3_] and [$N_{1112(\mathrm{OH})}$][C_4_F_9_SO_3_] are able to form ABSs, in contrast with [C_2_C_1_Im][CF_3_SO_3_] and [$N_{1112(\mathrm{OH})}$][CF_3_SO_3_], respectively, in combination with low-molecular-weight carbohydrates (glucose and sucrose) and/or a know globular protein stabilizer ([$N_{1112(\mathrm{OH})}$][H_2_PO_4_]), this means that the anion [C_4_F_9_SO_3_]^−^ has a higher ability to promote liquid–liquid demixing than [CF_3_SO_3_]^−^. The longer fluorinated alkyl chain of the anion renders a more hydrophobic character to the ionic liquid, enhancing the two-phase separation. The disruption of the hydrogen-bonding water networks results from a combined effect of the IL anion’s ability to hydrogen bond with water and the ion’s molar volume [47]. Further, the binding strength between the anions and the cations, along with the hydrophobic character and steric hindrance of the cations, are significant factors influencing interactions between water and ILs. As discussed above, no two-phase formation is achieved with the FIL containing cholinium cation in combination with low-molecular-weight carbohydrates (glucose and sucrose) or even with inorganic salts (K_3_PO_4_, K_2_HPO_4_, and K_3_citrate). [$N_{1112(\mathrm{OH})}$][C_4_F_9_SO_3_] only present liquid–liquid demixing with [$N_{1112(\mathrm{OH})}$][H_2_PO_4_] (Figure 1). The higher hydrophilicity of cholinium-based ILs was previously reported [3,18]. In a previous work of one of the authors [3] aiming at establishing more benign alternatives to the salts commonly used in ABS formation, [$N_{1112(\mathrm{OH})}$][C_4_F_9_SO_3_]was combined with a large number of carbohydrates (monosaccharides, disaccharides, and polyols) and no liquid–liquid demixing was observed. Generally, cholinium-based ILs only form ABSs with strong salting-out agents, such as K_3_PO_4_ [18], with polymers where the IL acts as the salting-out agent (the reverse effect) [48], or where more complex molecular phenomena appear to occur [49]. In this work, cholinium chloride present liquid–liquid demixing with strong salting-out salts (K_3_PO_4_ and K_2_HPO_4_) and no demixing is verified with the more benign K_3_citrate salt. The mere fluoro-containing IL [$N_{1112(\mathrm{OH})}$][CF_3_SO_3_] presents liquid–liquid demixing with the studied inorganic salts (K_3_PO_4_, K_2_HPO_4_, and K_3_citrate), and no demixing is verified with the known globular protein stabilizer [$N_{1112(\mathrm{OH})}$][H_2_PO_4_]. In contrast, [$N_{1112(\mathrm{OH})}$][C_4_F_9_SO_3_] allows two-phase formation with [$N_{1112(\mathrm{OH})}$][H_2_PO_4_] and no liquid–liquid demixing is verified with the three studied inorganic salts (Figure 1). The solubility of inorganic salts in [$N_{1112(\mathrm{OH})}$][C_4_F_9_SO_3_] could also be a process that interferes with ABS formation, particularly at lower water contents, potentiated by the peculiar behavior of FILs aqueous solutions, namely their complete miscibility in water due to the formation of distinct stable self-assembled structures [21] and that FILs reduce the impact of the addition of water upon the IL’s hydrogen-bond accepting ability. The solubility of low-molecular-weight carbohydrates in ionic liquids, which depends on the nature of the IL [50], on the length of the cation alkyl chains [51], and also on the molecular weight of the carbohydrate [52], interferes with ABS formation [3].

The formation of functionalized ABSs using the previously discussed FILs based on perfluoroalkylsulfonate anions requires a water-miscible ionic liquid. The implemented fluorinated ILs present distinct transitions due to the formation of stable self-assembled structures, supporting their complete miscibility in water [21]. The critical aggregation concentrations (CACs) of the FILs considered in the present work (Table 1), [C_2_C_1_Im][C_4_F_9_SO_3_] and [$N_{1112(\mathrm{OH})}$][C_4_F_9_SO_3_], are summarized in Table S23 of Supplementary Materials [21]. The Kamlet–Taft parameters of aqueous solutions of the studied FILs were determined to assess the hydrogen-bonding ability and polarizability [20], demonstrating that the impact of the addition of water upon the IL’s hydrogen-bond accepting ability is reduced by FILs, which is a key factor in the development of functionalized materials for applications in aqueous media (for example, extraction processes or dissolution of biomolecules). Further, in that study [20], it was also demonstrated that the networking of water aggregates is stimulated by the rich-aggregation behavior of these FILs, an upmost advantage for the application of these FILs in ABSs.

### 2.2. ABS Phase-Forming Component Effect on the Structure, Stability, and Function of Lysozyme

#### 2.2.1. Stability and Function of Lysozyme

The effect of the ABS phase-forming components, FILs ([C_2_C_1_Im][C_4_F_9_SO_3_] and [$N_{1112(\mathrm{OH})}$][C_4_F_9_SO_3_]), the mere fluoro-containing IL ([C_4_C_1_Im][CF_3_SO_3_]), known globular protein stabilizers ([$N_{1112(\mathrm{OH})}$][H_2_PO_4_] and sucrose), and high-charge density salt (K_3_PO_4_), on the activity of lysozyme was assessed using functional studies based on the muramidase activity of Lys. This activity was monitored spectrophotometrically at 450 nm, detecting the hydrolysis of the Micrococcus lysodeikticus cell wall by the enzyme (Section 3). The activity of lysozyme in water is considered to be 100% and used as a reference. The results of these functional studies (Figures S8–S13 of Supplementary Materials) show that the activity of lysozyme (0.2, 0.5, and 1.0 mg/mL) is not significantly affected by the ABS phase-forming components up to concentrations of 35 %wt (ABS biphasic region of overall systems; Tables S2–S21 and Figures S1–S6 of Supplementary Materials). The functionalized FILs maintain or slightly increase the enzymatic activity of Lys in high-concentrated FIL aqueous solutions, up to 35 %wt (the biphasic region of the FIL-based ABSs). In previous studies, the authors had already verified this behavior for concentrations across the FILs’ CACs (Table S23 of Supplementary Materials; up to ≈7.5 %wt FIL) [24,25]. This behavior was also verified by other authors with surfactant imidazolium-based ILs, although at lower IL concentrations (up to 15 mM), where the increment of lysozyme activity was attributed to conformational changes in the protein structure induced by ILs, stabilizing the integrity of the active site [53,54]. The results attained herein indicate that FILs, even at high concentrations, contribute to a better stabilization of the protein, which is reflected in an increase in lysozyme activity.

**Figure 6:**
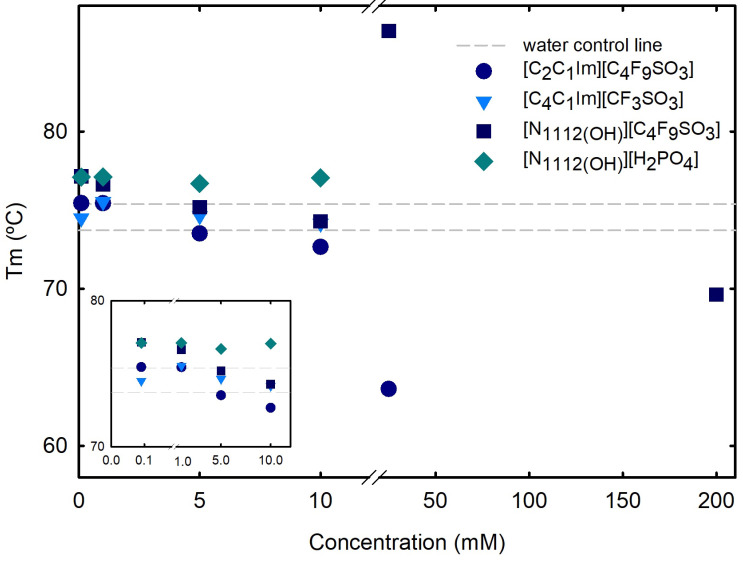
DSC lysozyme’s $T_{m}$ vs. ABS phase-forming component concentration. Lys’s $T_{m}$ in water is plotted as a traced line (control), 73.71 ± 0.004 °C. Snapshot of concentration range 0.1 to 10 mM in the lower-left corner. All Lys’s $T_{m}$s are summarized in Tables S24 and S28.

Differential scanning calorimetry (DSC) measurements (Section 3) were used to determine the melting temperature ($T_{m}$) and enthalpy (ΔH) of lysozyme, at which both the folded and unfolded states coexist at equilibrium. DSC outputs, thermal transitions at the nanoscale level, allow to analyze alterations on proteins structure and stability, providing sharp perceptions of the factors affecting stability, as well as of the folding and unfolding processes. A normalized DSC curve is depicted in Figure S14 of Supplementary Materials for 1.0 mg/mL Lys in water, allowing to determine a $T_{m}$ of 74.56 ± 0.835 °C, in agreement with other reports [24,25,55]. The effect of the following ABS phase-forming components, FILs ([C_2_C_1_Im][C_4_F_9_SO_3_] and [$N_{1112(\mathrm{OH})}$][C_4_F_9_SO_3_]), the mere fluoro-containing IL ([C_4_C_1_Im][CF_3_SO_3_]), and a known globular protein stabilizer ([$N_{1112(\mathrm{OH})}$][H_2_PO_4_]), on the stability of lysozyme is herein discussed. The $T_{m}$ of Lys, which characterizes the equilibrium between folded and unfolded states of the protein and is marked by a two-state transition [56,57], was measured in the absence and presence of [C_2_C_1_Im][C_4_F_9_SO_3_], [$N_{1112(\mathrm{OH})}$][C_4_F_9_SO_3_], [C_4_C_1_Im][CF_3_SO_3_], and [$N_{1112(\mathrm{OH})}$][H_2_PO_4_] at increasing concentrations (Table S24 of Supplementary Materials and Figure 6). For concentrations up to 10 mM (below the first CAC of both studied FILs, where only monomer behavior is verified; Table S23 of Supplementary Materials), all the analyzed ABS phase-forming components have no impact on Lys’s $T_{m}$. The effects of both FIL ([C_2_C_1_Im][C_4_F_9_SO_3_] and [$N_{1112(\mathrm{OH})}$][C_4_F_9_SO_3_]) concentrations on Lys’s $T_{m}$ above the first CAC (25 mM; Table S24 of Supplementary Materials), are Lys’s $T_{m}$ decrease with [C_2_C_1_Im][C_4_F_9_SO_3_] (DSC curve depicted in Figure S15 of Supplementary Materials) and increase with [$N_{1112(\mathrm{OH})}$][C_4_F_9_SO_3_] (DSC curve depicted in Figure S16 of Supplementary Materials). Additionally, with [$N_{1112(\mathrm{OH})}$][C_4_F_9_SO_3_], three transitions are verified (80.04 °C, 83.39 °C and 86.40 °C). The $T_{m}$ of lysozyme in aqueous solutions of the same FILs up to concentrations above the first CAC was measured in another work [24] using differential scanning fluorimetry (DSF), showing that the stability of Lys is not considerably affected in these conditions. Additionally, for the higher studied concentrations (55 mM, 120 mM, and 200 mM), to characterize the CACs above the first CAC (Table S23 of Supplementary Materials), no signal or insufficient signal was detected. For 200 mM [$N_{1112(\mathrm{OH})}$][C_4_F_9_SO_3_] (analyses of the third CAC; Table S23 of Supplementary Materials), a slight decrease of Lys’s $T_{m}$ is verified and four transitions are determined (Figure S17 of Supplementary Materials). These results are in agreement with a previous work by the authors [25], where the interaction of lysozyme with both FILs ([C_2_C_1_Im][C_4_F_9_SO_3_] and [$N_{1112(\mathrm{OH})}$][C_4_F_9_SO_3_]), protein–FIL complexes, and the encapsulation of Lys by the FILs was verified. In that work, the encapsulated lysozyme in [C_2_C_1_Im][C_4_F_9_SO_3_] aqueous solutions above the second and third CAC, upon centrifugation, was recovered and resuspended in the working buffer, allowing the determination of Lys’s $T_{m}$, which remained very close to the values of the Lys reference.

#### 2.2.2. Structural Properties of Lysozyme

**Figure 7:**
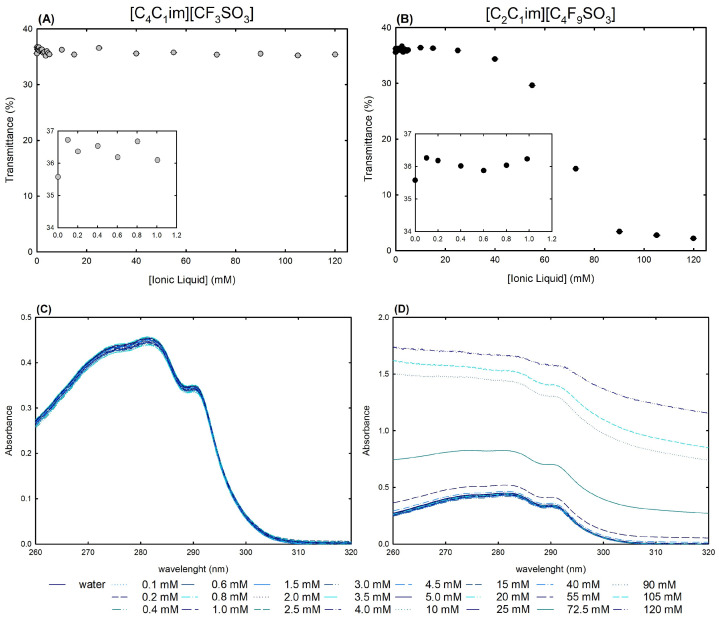
(**A**,**B**) Transmittance and (**C**,**D**) UV-VIS absorption spectra of 0.2 mg/mL lysozyme in aqueous solutions of [C_4_C_1_Im][CF_3_SO_3_] (mere fluoro-containing IL) and [C_2_C_1_Im][C_4_F_9_SO_3_] (FIL) (0–120 mM) at 25 °C.

As a first approach for the structural studies, UV-VIS spectrophotometry was used to measure the transmittance (Figure 7A,B) and absorption spectra (Figure 7C,D) of lysosyme 0.2 mg/mL in water and in twenty-four [C_2_C_1_Im][C_4_F_9_SO_3_] FIL and [C_4_C_1_Im][CF_3_SO_3_] mere fluoro-containing IL solutions ranging from 0.1 mM to 120 mM (encompassing all the CACs of the FIL; Table S23 of Supplementary Materials).

Streamlining, lysozyme (the isoelectric point of Lys is around 11.0 [58]) may interact with aqueous solutions of ionic liquids in two ways: electrostatic interactions, in which positively charged net lysozyme forms a neutral complex with ILs, resulting in a turbid aqueous solution; and non-electrostatic interactions, in which different levels of hydrophobicity lead to different interactions with the protein, resulting in different degrees of turbidity [25,26,59]. Monitoring the changes in turbidity offers an insight into the protein–ionic liquid aqueous solution interactions, which allows for the assessment of the ionic liquid functionalization of the fluorinated IL vs. the mere fluoro-contaning IL ([C_2_C_1_Im][C_4_F_9_SO_3_] vs. [C_4_C_1_Im][CF_3_SO_3_]). Figure 7A,B show the turbidity of lysozyme in terms of [C_4_C_1_Im][CF_3_SO_3_] and [C_2_C_1_Im][C_4_F_9_SO_3_] aqueous solution concentrations at a fixed 0.2 mg/mL Lys concentration. Since Lys + [C_4_C_1_Im][CF_3_SO_3_] aqueous solutions exhibit a steady turbidity profile with increasing IL concentrations, it is reasonable to establish that turbidity is independent of the IL concentration and no substantial interactions may occur. In opposition, [C_2_C_1_Im][C_4_F_9_SO_3_] FIL revealed a sharp decrease in transmittance after 15 mM (corresponding to the first CAC; Table S23 of Supplementary Materials), as a result of strong interactions between lysozyme and FIL, protein–FIL complexes, and lysosyme encapsulation by the FIL aggregates, which were verified by the authors in previous works [24,25,28]. The results of Rather et al. [59] also confirmed the significant composition and concentration dependence of the lysozyme–surfactant IL interactions, mainly in the CAC concentration regime. Additionally, an increase in turbidity is observed for both [C_4_C_1_Im][CF_3_SO_3_] and [C_2_C_1_Im][C_4_F_9_SO_3_] at concentrations below the first CAC of the FIL (only monomers exist in solution for both ionic liquids) in comparison with lysozyme in water (snapshots in Figure 7A,B). This increased turbidity might be related to the electrostatic interactions between the lysozyme positively charged net and the [C_4_C_1_Im][CF_3_SO_3_] or [C_2_C_1_Im][C_4_F_9_SO_3_] negative counterpart, resulting in an increase in turbidity [60,61]. Still, [C_2_C_1_Im][C_4_F_9_SO_3_] and [C_4_C_1_Im][CF_3_SO_3_] have differing behaviors up to 1.0 mM; the FIL exhibited a U-shaped profile with a minimum transmittance around 0.6 mM, whereas a random turbidity fluctuation was observed for the mere fluoro-containing IL.

Absorption spectra of proteins and ionic liquid aqueous solutions are very useful to obtain insights into the interactions between proteins and ionic liquids [25,28,53,54,56,60,62]. Aromatic amino acid residues from lysozyme, specifically six tryptophan residues, can be used as labels to identify changes in protein structure and conformation. These residues can be found in the active site or in the hydrophobic region of the protein [53,54]. The wavelength of these amino acids lies between 260 nm and 300 nm, presenting a characteristic maximum peak at 280 nm. Any peak deviations might indicate modifications occurring in the local environment of the characteristic lysozyme tryptophan residues. Typically, these changes are based on the shift to lower or higher wavelength values of the absorbance maximum that can indicate protein conformation changes. Additionally, several works report an increase or decrease in absorbance intensity in the presence of different families of ionic liquids [25,28,53,54,56,60,62]. The UV-VIS absorption spectra of Lys 0.2 mg/mL at varying concentrations of [C_4_C_1_Im][CF_3_SO_3_] a mere fluoro-containing IL, are depicted in Figure 7C. The characteristic lysozyme spectra were maintained at all concentrations (0.1–120 mM; up to ≈3.5 %wt IL) in comparison to lysozyme 0.2 mg/mL in water (lysozyme reference, also depicted in the figure). For the aqueous solutions of lysozyme 0.2 mg/mL with the [C_2_C_1_Im][C_4_F_9_SO_3_] FIL in the same concentration range (0.1–120 mM; up to ≈5.0 %wt FIL), encompassing all four FIL self-aggregates (120 mM above of the fourth CAC FIL; Table S23 of Supplementary Materials), Lys spectra show no deviation of the maximum peak and have the same profile as lysozyme 0.2 mg/mL in water, indicating no conformation change of the protein (Figure 7D). In opposition to the solutions with the [C_4_C_1_Im][CF_3_SO_3_] mere fluoro-containing IL, turbidity is observed in all the Lys solutions with [C_2_C_1_Im][C_4_F_9_SO_3_] FIL above 25 mM (above the first CAC of FIL), and for higher FIL concentrations in the CAC concentration regime, the spectra lose baseline and become a flat line. The coverage of the protein surface by the [C_2_C_1_Im][C_4_F_9_SO_3_] FIL can be suggested by the absence of the characteristic absorption band in the absorption spectra, or suggested by the turbidity of the solution as a result of the presence of protein–FIL complexes or encapsulation of Lys by [C_2_C_1_Im][C_4_F_9_SO_3_] aggregates. This result is not attributed to unfolding once the unfolding process is detected through a shift of the peak absorbance to values around 301 nm, as previously reported [63].

**Figure 8:**
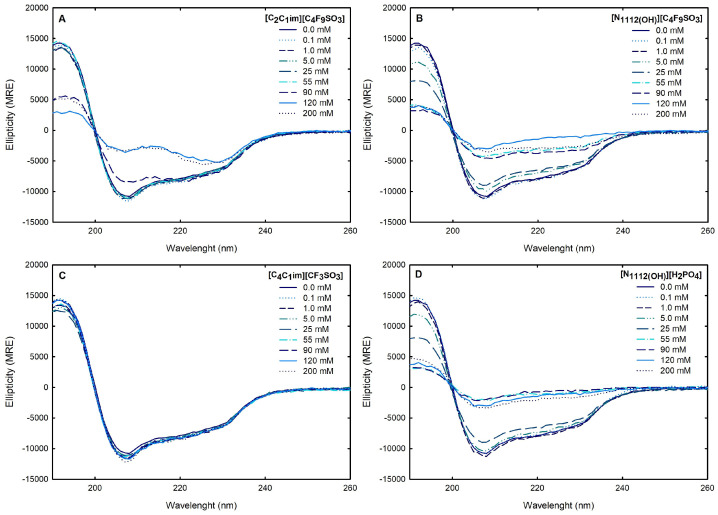
CD spectra of 1.0 mg/mL lyzozyme in water, (**A**) 0.1–200 mM [C_2_C_1_Im][C_4_F_9_SO_3_], (**B**) 0.1–200 mM [$N_{1112(\mathrm{OH})}$][C_4_F_9_SO_3_], (**C**) 0.1–200 mM [C_4_C_1_Im][CF_3_SO_3_] and (**D**) 0.1–200 mM [$N_{1112(\mathrm{OH})}$][C_4_F_9_SO_3_]. All spectra were acquired at 25 °C.

**Figure 9:**
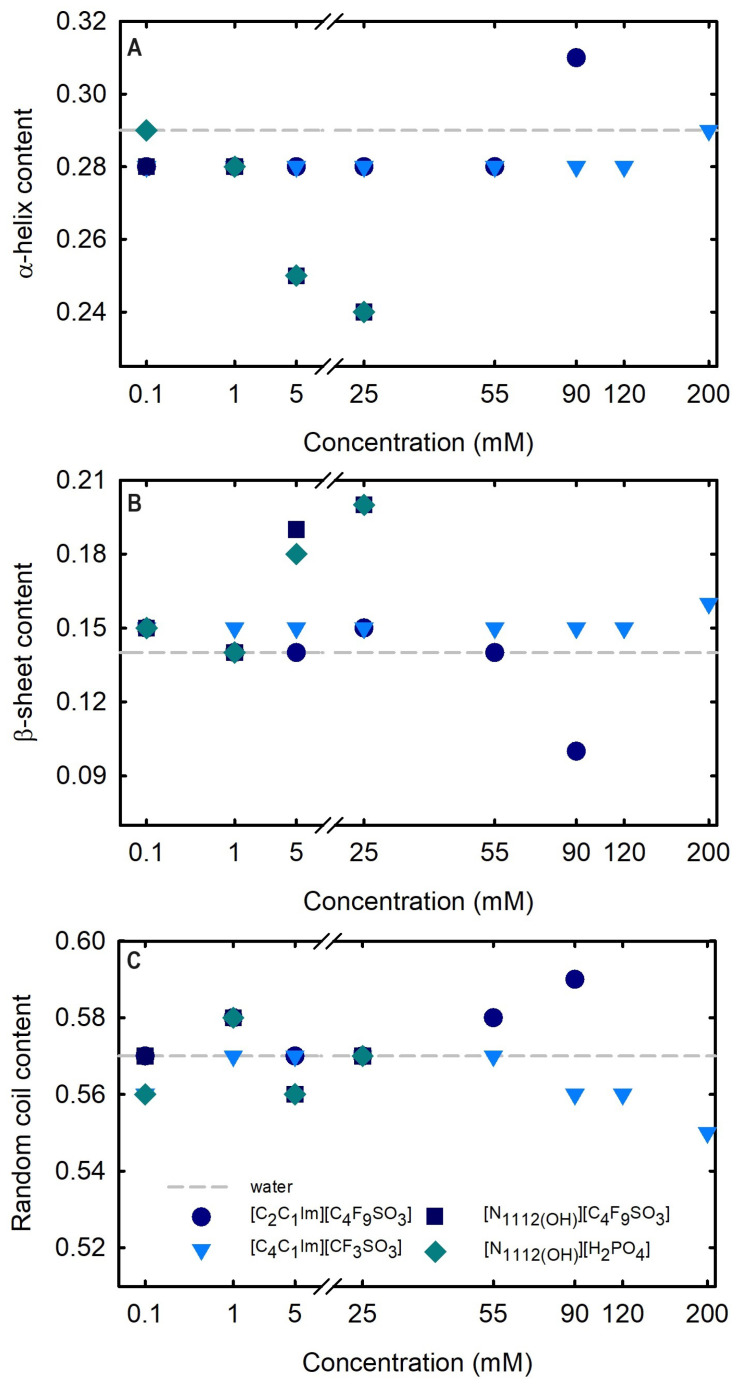
Effect of ABS phase-forming component concentration on the estimated structural content ((**A**) α-helix, (**B**) β-strand, and (**C**) random coil). Determined structural content of Lys 1.0 mg/mL in water is plotted as traced line (control). All Lys structural content (α-helix, β-strand, and random coil) is summarized in Table S25 of Supplementary Materials.

Circular dichroism (CD) spectroscopy is broadly applied to assess the secondary structure of proteins because different structural elements have characteristic CD spectra [64]. Far-UV (190–260 nm) CD spectroscopy was used to assess Lys secondary structure. The spectrum obtained at 25 °C for 1.0 mg/mL Lys in water is presented in Figure 8. The effects of the FILs [C_2_C_1_Im][C_4_F_9_SO_3_] and [$N_{1112(\mathrm{OH})}$][C_4_F_9_SO_3_] the mere fluoro-containing [C_4_C_1_Im][CF_3_SO_3_], and [$N_{1112(\mathrm{OH})}$][H_2_PO_4_] on Lys CD spectra are also depicted in Figure 8 with incremental concentrations from 0.1 mM to 200 mM (encompassing all the CACs for FILs; Table S23 of Supplementary Materials). The protein relative content of all the secondary structural features (α-helix, β-sheet, and random coil) was predicted using the K2D3 algorithm [65] for Lys in water and in the aqueous solutions of the ABS phase-forming components, as depicted in Table S25 of Supplementary Materials and Figure 9. The secondary structure predictions obtained for lysozyme, α-helix of 29% and 14% of β-strand, are in good agreement with the analysis based on various methods [24,64] and yielding values of ≈30% and ≈19%, respectively. For [C_4_C_1_Im][CF_3_SO_3_] (mere fluoro-containing IL), the structural content was estimated for the concentration range (0.1–200 mM). For both [$N_{1112(\mathrm{OH})}$][C_4_F_9_SO_3_] FIL and [$N_{1112(\mathrm{OH})}$][H_2_PO_4_] (known globular protein stabilizer), the structural content was only estimated up to 25 mM (above the first of [$N_{1112(\mathrm{OH})}$][C_4_F_9_SO_3_]). For the other FIL, [C_2_C_1_Im][C_4_F_9_SO_3_], the structural content was estimated up to 90 mM (above the third CAC; Table S23 of Supplementary Materials). No substantial protein structural modification upon the ABS phase-forming component addition was verified. A similar result was attained in a previous work [24] for cholinium-based FILs ([$N_{1112(\mathrm{OH})}$][C_4_F_9_SO_3_] and [$N_{1112(\mathrm{OH})}$][C_4_F_9_CO_2_]) and [$N_{1112(\mathrm{OH})}$][H_2_PO_4_] at concentrations below and above the first CAC of both FILs. The pairwise comparison of CD spectra with the different ABS phase-forming components (at all concentrations) with the Lys reference (Lys in water) suggests no substantial protein structural modification upon ABS phase-forming components at concentrations below and above the high-order CAC of both FILs (up to 200 mM).

**Figure 10:**
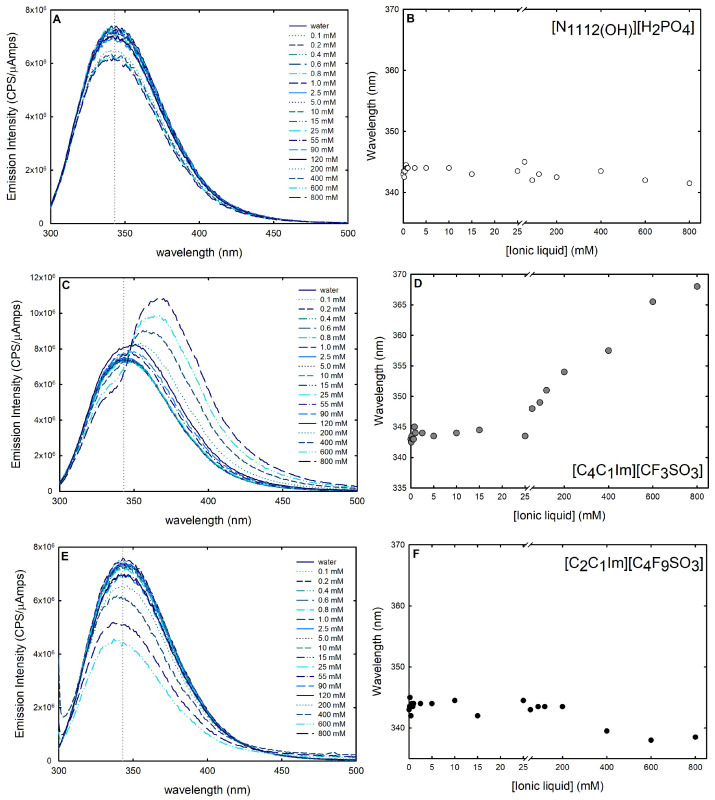
Intrinsic fluorescence spectra of lysozyme 1.0 mg/mL in the presence of (**A**) 0.1–800 mM [$N_{1112(\mathrm{OH})}$][H_2_PO_4_] (globular protein stabilizer), (**C**) 0.1–800 mM [C_4_C_1_Im][CF_3_SO_3_] (mere fluoro-containing IL), and (**E**) 0.1–800 mM [C_2_C_1_Im][C_4_F_9_SO_3_] (fluorinated IL). Lysozyme changes in intrinsic fluorescence in (**B**) 0.1–800 mM [$N_{1112(\mathrm{OH})}$][H_2_PO_4_] (globular protein stabilizer), (**D**) 0.1–800 mM [C_4_C_1_Im][CF_3_SO_3_] (mere fluoro-containing IL) and (**F**) 0.1–800 mM [C_2_C_1_Im][C_4_F_9_SO_3_] (fluorinated IL) are shown at maximum wavelength (vertical dashed line in (**A**,**C**,**E**)). All spectra were acquired at 25 °C.

In addition to CD studies, fluorescence spectroscopy was used to further investigate the changes in lysozyme stability and conformational dynamics in the presence of the following ABS phase-forming components: [C_2_C_1_Im][C_4_F_9_SO_3_] (FIL), [C_4_C_1_Im][CF_3_SO_3_] (mere fluoro-containing IL), and [$N_{1112(\mathrm{OH})}$][H_2_PO_4_] (globular protein stabilizer). Intrinsic fluorescence signals, on account of fluorescence-emitting amino acids (phenylalanine, tyrosine, or tryptophan) present in the sequence of a protein, are highly sensitive to the protein’s conformational changes and, hence, can be exploited as a probe to explore both unfolding and protein–ligand interactions. Even though large changes in protein conformation can modify the intensity of tyrosine and phenylalanine fluorescence signals, their low extinction coefficients and quantum yields, as well as their relative lower sensitivity to environmental changes, make them a less appealing candidate [40]. Tryptophan is frequently found concealed in the hydrophobic core of proteins due to its aromatic nature. Hence, these side chains become more solvent-exposed upon disruption of the protein’s tertiary or quaternary structure. The tryptophan release energy is particularly sensitive to the polarity (and dynamics) of the environment because the excited-state dipole moment is quite large [62,66]. Reactions to denaturation, substrate binding, subunit association, and conformational transitions create variations in the tryptophan emission spectra [40]. The spectrum shift generally known as blue shift, a shift of the maximum emission towards smaller wavelengths, denotes that the aromatic residue is buried in the native structure of the protein [67]. A shift of the maximum emission towards larger wavelengths, known as red shift, suggests an exposition of the tryptophan and is related with the unfolded state of the protein structure [67]. Thus, a red shift of the emission spectrum suggests a destructive impact on the protein’s structural features by the ligand, and a blue shift is synonymous with a beneficial effect on the structure of the protein. Furthermore, the amount of exposure of the fluorophores to the solvent is associated with the increase or decrease in the intensity of the emission spectrum. Tryptophan residues were selectively excited at 295 nm to prevent the response of other aromatic residues (e.g., tyrosine) [67], and the emission spectra of intrinsic fluorescence of lysozyme 0.2 mg/mL in water and upon successive additions of [C_2_C_1_Im][C_4_F_9_SO_3_] [C_4_C_1_Im][CF_3_SO_3_] and [$N_{1112(\mathrm{OH})}$][H_2_PO_4_] (up to 800 mM; ≈25 %wt [C_2_C_1_Im][C_4_F_9_SO_3_], concentration range in the ABS) were recorded from 300 nm to 500 nm wavelengths (Figure 10). Variations in lysozyme spectra are caused by ligand-induced changes in the microenvironment of tryptophan residues, either by shifting the maximum peak wavelength (blue or red shift) or by altering peak intensities. These changes can be used as a measure of the effect of the ABS phase-forming components on protein folding and unfolding [28].

The known protein stabilizer [$N_{1112(\mathrm{OH})}$][H_2_PO_4_] is thought to operate in a similar mechanism as some other stabilizing salts in preserving the folded conformation of protein, shielding repulsive interactions, stabilizing surface electrostatics, and minimizing the protein propensity for unfolding [68]. In the presence of an increasing concentration of [$N_{1112(\mathrm{OH})}$][H_2_PO_4_] (up to 800 mM; ≈15 %wt), a reduction in fluorescence emission intensity is observed (Figure 10A), while the maximum emission wavelength remains constant (Figure 10B), meaning that [$N_{1112(\mathrm{OH})}$][H_2_PO_4_] contributes to a more folded conformation. A similar behavior is observed for the FIL [C_2_C_1_Im][C_4_F_9_SO_3_] (up to 800 mM; ≈25 %wt); no detectable shift in the fluorescence maximum peak wavelength is verified (Figure 10F), and a steady decline in the lysozyme intrinsic fluorescence is observed (Figure 10E) for concentrations above the first CAC (Table S23 of Supplementary Materials). A plateau in the fluorescence emission intensity is observed up to ≈15 mM. Above 15 mM (above the first CAC; FIL aggregates in solution), a reduction in fluorescence emission intensity is observed. The quenching of the emission intensity is prompted by the aggregation of the FIL, suggesting that the interaction with the FIL hides the fluorophores from the solvent. Further, the fluorescence maximum peak wavelength remains constant (Figure 10F), with a slight blue shift above 200 mM for [C_2_C_1_Im][C_4_F_9_SO_3_], indicating that [C_2_C_1_Im][C_4_F_9_SO_3_] has a productive behavior towards lysozyme in the range of concentrations studied (0.1–800 mM). In opposition, with increasing concentrations of [C_4_C_1_Im][CF_3_SO_3_] (up to 800 mM; ≈19 %wt), an increase in Lys fluorescence emission intensity is observed (Figure 10C). Further, above 25 mM, the maximum emission wavelength presents a red shift from 334 nm to 368 nm (Figure 10D). This implies protein denaturation above this concentration of [C_2_C_1_Im][C_4_F_9_SO_3_]. A red shift at maximum wavelength is usually related to globular protein unfolding [69]. These results show the relevant role of FIL self-aggregation structures on the interaction with lysozyme and on the conformation stability of lysozyme. Rather and their colleagues [59] also found that surfactant imidazolium-based ILs significantly enhance the conformational stability of lysozyme in the CAC concentration regime. Further, the results attained herein corroborate and support a previous study by the authors on the intrinsic fluorescence of IFN-α2b with FILs ([C_2_C_1_Im][C_4_F_9_SO_3_] and [$N_{1112(\mathrm{OH})}$][C_4_F_9_SO_3_]) [28].

### 2.3. Lysozyme Partition in Functionalized ABS

#### 2.3.1. Extraction Efficiency (%EE)

The influence of twelve biphasic systems summarized in Table 1, comprising ionic-liquid-based bottom-phase and FILs ([C_2_C_1_Im][C_4_F_9_SO_3_] and [$N_{1112(\mathrm{OH})}$][C_4_F_9_SO_3_]) vs. mere fluoro-containing ILs ([C_4_C_1_Im][CF_3_SO_3_] and [C_2_C_1_Im][CF_3_SO_3_]), and non-ionic-liquid-based top-phases (K_3_PO_4_ [$N_{1112(\mathrm{OH})}$][H_2_PO_4_] glucose, and sucrose), on Lys partition was studied. The phase diagrams of the selected systems are detailed in Section 2.1. Table 1 outlines the volume ratio, pH, and composition of both the ionic-liquid phase (bottom phase) and the non-ionic liquid phase (top phase) of the selected systems. Lys partition behavior in each biphasic system (biphasic point, BP) is described in terms of extraction efficiency (%EE, Equation (6)). All BPs were selected considering the concentration regions where the activity of lysozyme is maintained (screening enzymatic activity of Lys, Section 2.2.1 in [C_2_C_1_Im][C_4_F_9_SO_3_] (FIL), [C_4_C_1_Im][CF_3_SO_3_] (mere fluoro-containing IL), known globular protein stabilizers ([$N_{1112(\mathrm{OH})}$][H_2_PO_4_] and sucrose), and high-charge density salt (K_3_PO_4_) aqueous solutions. Additionally, all BPs are equidistant to each binodal curve, maintaining the ionic liquid concentration fixed at 30 %wt (FIL vs. mere fluoro-containing IL). The %EE results for lysozyme are summarized in Table S26 of Supplementary Materials. The precise quantification of Lys was determined through UV-VIS spectroscopy and BCA protein assay (Section 3). Additionally, the quantification of BSA was determined through MICRO BCA and Bradford protein assays for some specific biphasic systems (FIL-based ABSs due to the surfactant nature of FILs; carbohydrate-based ABS, especially with OH-reductive glucose). A MICRO BCA protein assay is fitted for dilute protein samples, allowing to work with higher dilution factors for the ABS phase samples, which avoids any interference of the ABS phase-forming components on the quantification of protein. A combination of colorimetric protein assays with UV-VIS was implemented to validate the colorimetric protein assays as an accurate procedure for the determination of protein concentration with the most sample types from functionalized ABS phases. The implemented protein assays afford the sample handling ease of a microplate and require a smaller volume of protein sample, MICRO BCA in particular, enabling increased ABS output and miniaturization experiments [19]. All the results are in good agreement. Further, the precise characterization of the protein composition within each phase of two studied ABSs (BP#3 and BP#5), for simultaneous partition of Lys and BSA, was attained using SDS-PAGE analysis (Section 2.3.3).

**Figure 11:**
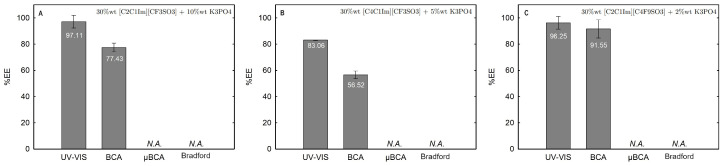
Lys extraction efficiency (%EE; Equation (6)) at 25 °C for the biphasic systems (**A**) BP#1 30 %wt [C_2_C_1_Im][CF_3_SO_3_]+ 10 %wt K_3_PO_4_, (**B**) BP#2 30 %wt [C_4_C_1_Im][CF_3_SO_3_] + 5 %wt K_3_PO_4_ and (**C**) BP#3 30 %wt [C_2_C_1_Im][C_4_F_9_SO_3_] + 2 %wt K_3_PO_4_. All %EE are summarized in Table S26 of Supplementary Materials.

**Figure 12:**
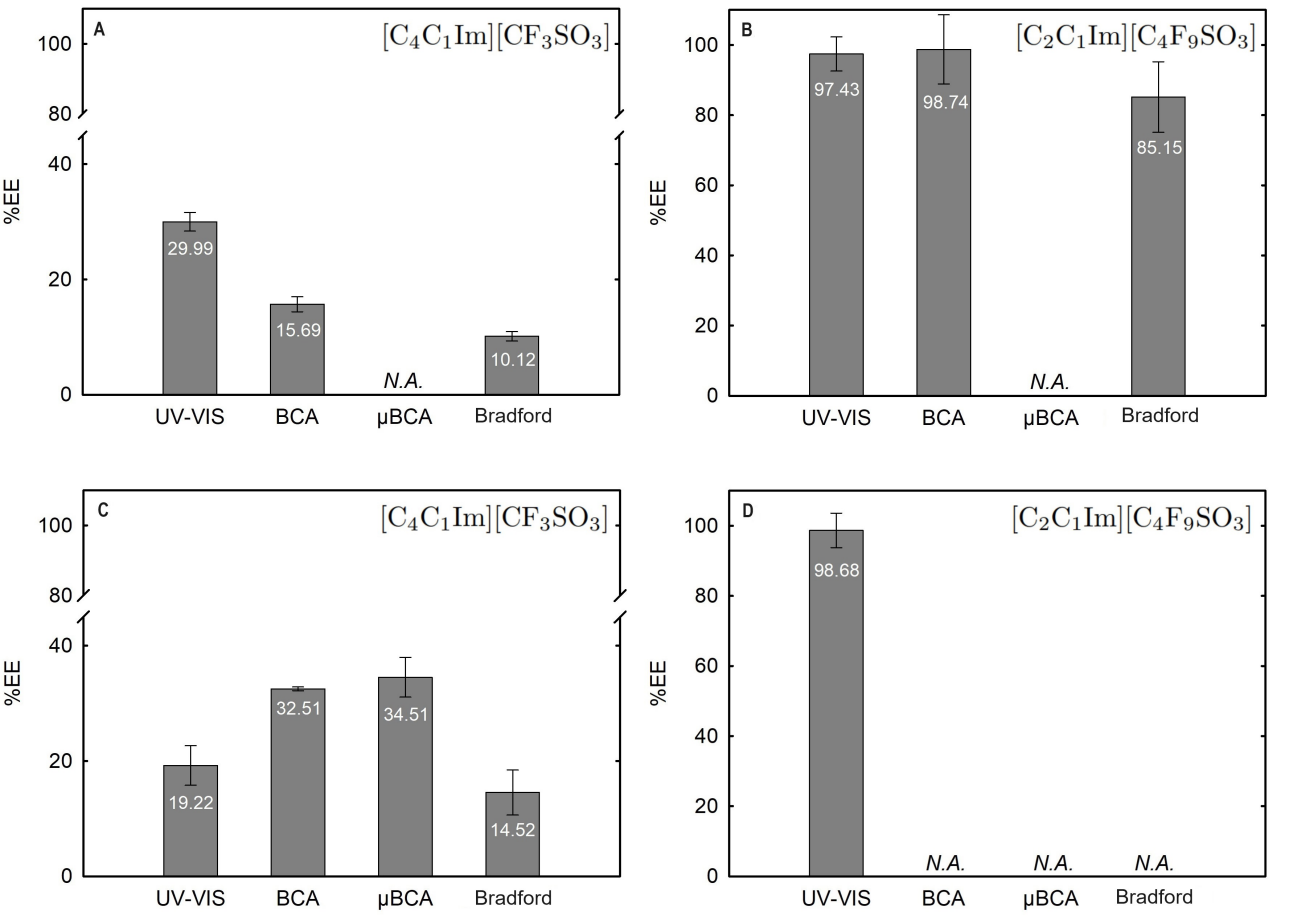
Lys extraction efficiency (%EE; Equation (6)) at 25 °C for the biphasic systems (**A**) BP#4 30 %wt [C_4_C_1_Im][CF_3_SO_3_] + 25 %wt sucrose, (**B**) BP#5 30 %wt [C_2_C_1_Im][C_4_F_9_SO_3_] + 25 %wt sucrose, (**C**) BP#6 30 %wt [C_4_C_1_Im][CF_3_SO_3_] + 25 %wt glucose, and (**D**) BP#7 30 %wt [C_2_C_1_Im][C_4_F_9_SO_3_] + 25 %wt glucose. All %EE are summarized in Table S26 of Supplementary Materials.

**Figure 13:**
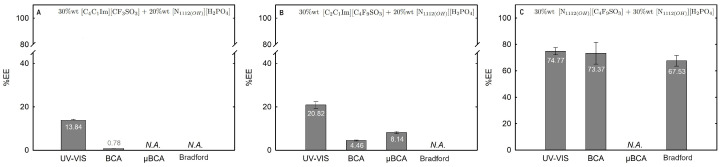
Lys extraction efficiency (%EE; Equation (6)) at 25 °C for the biphasic systems (**A**) BP#11 30 %wt [C_4_C_1_Im][CF_3_SO_3_] + 20 %wt [$N_{1112(\mathrm{OH})}$][H_2_PO_4_], (**B**) BP#10 30 %wt [C_2_C_1_Im][C_4_F_9_SO_3_] + 20 %wt [$N_{1112(\mathrm{OH})}$][H_2_PO_4_] and (**C**) BP#12 30 %wt [$N_{1112(\mathrm{OH})}$][C_4_F_9_SO_3_] + 30 %wt [$N_{1112(\mathrm{OH})}$][H_2_PO_4_]. All %EE are summarized in Table S26 of Supplementary Materials.

**Figure 14:**
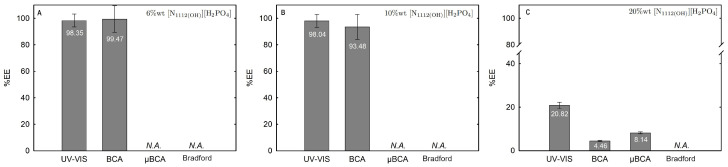
Lys extraction efficiency (%EE; Equation (6)) at 25 °C for the biphasic systems (**A**) BP#8 30 %wt [C_2_C_1_Im][C_4_F_9_SO_3_] + 6 %wt [$N_{1112(\mathrm{OH})}$][H_2_PO_4_], (**B**) BP#9 30 %wt [C_2_C_1_Im][C_4_F_9_SO_3_] + 10 %wt [$N_{1112(\mathrm{OH})}$][H_2_PO_4_] and (**C**) BP#10 30 %wt [C_2_C_1_Im][C_4_F_9_SO_3_] + 20 %wt [$N_{1112(\mathrm{OH})}$][H_2_PO_4_]. All %EE are summarized in Table S26 of Supplementary Materials.

Lysozyme extraction efficiency (%EE) for all the twelve biphasic systems are summarized in Table S26 of Supplementary Materials and depicted in Figure 11 for the systems combined with a high-charge density salt (FIL vs. mere fluoro-containing IL): 30 wt% [C_2_C_1_Im][CF_3_SO_3_] + 10 wt% K_3_PO_4_ (BP#1), 30 wt% [C_4_C_1_Im][CF_3_SO_3_] + 5 wt% K_3_PO_4_ (BP#2), and 30 wt% [C_2_C_1_Im][C_4_F_9_SO_3_] + 2 wt% K_3_PO_4_ (BP#3); Figure 12 for the systems combined with low-molecular-weight carbohydrates (FIL vs. mere fluoro-containing IL): 30 wt% [C_4_C_1_Im][CF_3_SO_3_] + 25 wt% sucrose (BP#4), 30 wt% [C_2_C_1_Im][C_4_F_9_SO_3_] + 25 wt% sucrose (BP#5), 30 wt% [C_4_C_1_Im][CF_3_SO_3_] + 25 wt% glucose (BP#6), and 30 wt% [C_2_C_1_Im][C_4_F_9_SO_3_] + 25 wt% glucose (BP#7); Figure 13 for the systems combined with a protein stabilizer (FIL vs. mere fluoro-containing IL): 30 wt% [C_4_C_1_Im][CF_3_SO_3_] + 20 wt% [$N_{1112(\mathrm{OH})}$][H_2_PO_4_] (BP#11), 30 wt% [C_2_C_1_Im][C_4_F_9_SO_3_] + 20 wt% [$N_{1112(\mathrm{OH})}$][H_2_PO_4_] (BP#10), and 30 wt% [$N_{1112(\mathrm{OH})}$][C_4_F_9_SO_3_] + 30 wt% [$N_{1112(\mathrm{OH})}$][H_2_PO_4_] (BP#12); and Figure 14 for the systems combined with a protein stabilizer (conc. effect): 30 wt% [C_2_C_1_Im][C_4_F_9_SO_3_] + 6 wt% [$N_{1112(\mathrm{OH})}$][H_2_PO_4_] (BP#8), 30 wt% [C_2_C_1_Im][C_4_F_9_SO_3_] + 10 wt% [$N_{1112(\mathrm{OH})}$][H_2_PO_4_] (BP#9), and 30 wt% [C_2_C_1_Im][C_4_F_9_SO_3_] + 20 wt% [$N_{1112(\mathrm{OH})}$][H_2_PO_4_] (BP#10). Proteins are categorized as charged molecules [70]. The pH of each phase of the ABSs can impact the partitioning behavior of Lys, since the charge characteristics of proteins depend on the pH. The isoelectric point of Lys is around 11.0 [58], and it is positively charged under acidic conditions and at neutral pH.

In the K_3_PO_4_-based systems (BP#1, BP#2, and BP#3), both bottom and top phases are at higher pH values, and Lys is negatively charged. The Lys-enriched phase is the ionic-liquid-rich phase, which is a mere fluoro-containig IL ([C_2_C_1_Im][CF_3_SO_3_] and [C_4_C_1_Im][CF_3_SO_3_]) or fluorinated IL ([C_2_C_1_Im][C_4_F_9_SO_3_]) (Table S26 of Supplementary Materials; Figure 11). In conventional inorganic salt-based ABSs, practically all proteins are negatively charged due to the high pH value, and the negatively charged proteins are repelled from the inorganic salt-rich phases and enriched to the non-inorganic salt phase [71]. The results for Lys follow the conventional partition trend. For other globular proteins (BSA), functionalized FIL-based ABSs in combination with high-charge density salts allows more amenable ABSs, allowing lower quantities of K_3_PO_4_ for demixing and the selection of the enriched phase [19]. BSA partition to the inorganic salt-rich phase (not repelled), suggesting that BP#3 allows a single-step selective purification of Lys and BSA (discussed in detail in the following Section 2.3.3). For the four biphasic systems combined with low-molecular-weight carbohydrates, sucrose and glucose, all phases are near neutral pH (5.00–6.75), and Lys is positively charged. For the system based on the mere fluoro-containing IL ([C_4_C_1_Im][CF_3_SO_3_]; BP#4 and BP#6), the Lys-enriched phase is the carbohydrate-rich phase. For the FIL-based system ([C_2_C_1_Im][C_4_F_9_SO_3_]; BP#5 and BP#7), Lys partitions to the FIL-rich phase. The results highlighted in Figure 12 show that these functionalized ABSs, [C_4_C_1_Im][CF_3_SO_3_] (mere fluoro-containing IL) vs. [C_2_C_1_Im][C_4_F_9_SO_3_] (fluorinated IL), in combination with glucose or sucrose (a known globular protein stabilizer), grant the selection of the enriched phase, a benign low-molecular-weight carbohydrate-rich phase [3] or a biocompatible FIL-rich phase [24,25,26,28,72]. Lys is positively charged in all [$N_{1112(\mathrm{OH})}$][H_2_PO_4_]-based ABSs, combined with mere fluoro-containing ILs ([C_4_C_1_Im][CF_3_SO_3_], BP#11) or combined with FIls ([C_2_C_1_Im][C_4_F_9_SO_3_], BP#8, BP#9, and BP#10; [$N_{1112(\mathrm{OH})}$][C_4_F_9_SO_3_], BP#12). For [C_2_C_1_Im][C_4_F_9_SO_3_]-based ABSs (Figure 14), up to 10 wt% [$N_{1112(\mathrm{OH})}$][H_2_PO_4_] Lys-enriched phase is the FIL-rich phase (BP#8 and BP#10), and for a higher [$N_{1112(\mathrm{OH})}$][H_2_PO_4_] concentration (20 wt%, BP#10), Lys partitions to the [$N_{1112(\mathrm{OH})}$][H_2_PO_4_]-rich phase. For the biphasic systems with higher [$N_{1112(\mathrm{OH})}$][H_2_PO_4_] concentration (20 wt%, BP#10 and BP#11; 30 wt%, BP#12), the Lys-enriched phase is the [$N_{1112(\mathrm{OH})}$][H_2_PO_4_]-rich phase for the ABSs based on [C_4_C_1_Im][CF_3_SO_3_] (BP#11) and [C_2_C_1_Im][C_4_F_9_SO_3_] (BP#10), and for the ABSs based on [$N_{1112(\mathrm{OH})}$][C_4_F_9_SO_3_] Lys partitions to the FIL-rich phase, as depicted in Figure 13.

The phase properties and volume ratio of the twelve biphasic systems are detailed in Table 1. The effect of ABS phase pH on Lys’s %EE was detailed previously. The impact of other relevant phase properties, including ionic-liquid-based bottom-phase (FILs, ([C_2_C_1_Im][C_4_F_9_SO_3_] and [$N_{1112(\mathrm{OH})}$][C_4_F_9_SO_3_]), or mere fluoro-containing ILs, [C_4_C_1_Im][CF_3_SO_3_] and [C_2_C_1_Im][CF_3_SO_3_]) and non-ionic-liquid-based top-phases (K_3_PO_4_ sucrose, glucose, and [$N_{1112(\mathrm{OH})}$][H_2_PO_4_]), on Lys’s %EE, namely, amount of water (%wt H_2_O), amount of mere fluoro-containing IL or FIL (%wt IL/FIL), amount of K_3_PO_4_, sucrose, glucose, or [$N_{1112(\mathrm{OH})}$][H_2_PO_4_] (%wt non-IL/FIL), and volume ratio, was assessed. No clear trend was observed in Lys’s %EE with phase properties and volume ratio.

The partition behavior of other globular proteins (BSA) and IFN-α2b in eight of the ABSs outlined in Table 1 was studied in a concurrent work [19], where a single-step simultaneous purification of IFN-α2b and BSA was assessed using these functionalized ABSs. More details on the partition behavior of IFN-α2b and BSA can be found elsewhere [19]. The single-step simultaneous purification of the two globular proteins Lys and BSA will be assessed in the following Section 2.3.3. In previous works from the authors [24,25,26,28,72], biocompatible perfluoroalkylsulfonate-based fluorinated ILs were developed, allowing complex formation between the FIL aggregates and distinct proteins, namely Lys, BSA, and IFN-α2b. Furthermore, two concurrent works based on the development of functionalized ABSs based on these perfluoroalkylsulfonate FILs for the selective partition of IFN-α2b and BSA [19], and Lys (assessed in the present study), were attained. The results point to the existence of other properties besides the protein charge or solvent-accessible surface area that decide protein–FIL interactions, such as the third ABS phase-forming component. Overall, the results prove that FIL-based ABSs are more versatile and amenable to be tuned, allowing the use of more benign phase-forming components, namely sucrose or [$N_{1112(\mathrm{OH})}$][H_2_PO_4_](know globular protein stabilizers), instead of high-charge density salts, and select the enriched phase.

#### 2.3.2. Structure, Stability, and Function of Lysozyme in ABS Phases

Along with the characterization of protein partition behavior in the selected ABSs, using the extraction efficiency (%EE) parameter, it is mandatory that the proteins show no denaturation and that the activity is maintained. The efficacy and safety of therapeutic proteins depend on their folded conformation, and various manipulations during the extraction steps have the potential to modify the active ingredient. Enzyme assays, standardized experimental protocols mostly based upon the detection of luminescent, fluorescent, or spectrophotometric endpoint signals, are well established in order to measure the activity of proteins by providing precise measurements of enzymatic reactions [24,27,73,74]. CD allows to analyze the structural changes occurring when a protein interacts with other molecules [24,26,27,28,75] (and references cited therein), as well as DSC, a thermoanalytical technique often used to address protein folding and stability [76]. In this work, the impact of the ABS phase on the function, stability and structure of Lys was attained in comparison with the behavior of the protein in water. Enzymatic assays, DSC, and CD were used to address the function, stability, and structure of Lys and to understand potential alterations prompted during the ABS extraction step.

**Figure 15:**
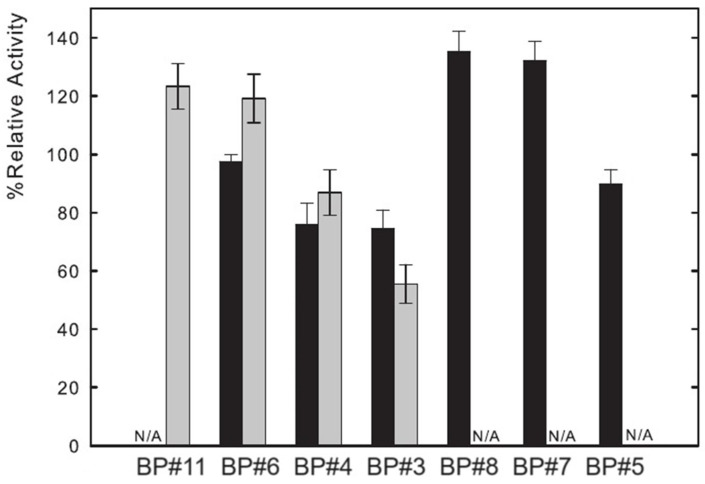
Relative enzymatic activity of partition lysozyme in both phases of seven of the biphasic systems summarized in Table 1. The results are shown in the following order: first, the results for [C_4_C_1_Im][CF_3_SO_3_] (mere fluoro-containing IL), and second, the results for [C_2_C_1_Im][C_4_F_9_SO_3_] (fluorinated IL). Black bars indicate ionic-liquid-rich phase and grey bars indicate non-ionic-liquid-rich phase (N/A, phases where EA was not technically feasible).

Lysozyme catalyzes the hydrolysis of β-(1→ 4) glucosidic linkages between N-acetyl glucosamine and N-acetylmuramic acid. The muramidase activity of Lys was assessed by measuring the absorbance decrease at 450 nm (Section 3), which reflects the hydrolysis of the Micrococcus lysodeikticus cell wall by the enzyme. Several parameters impact this activity, such as pH (optimal value pH = 6.2) and temperature [24,74,77]. Thus, functional experiments were performed with partition Lys in ABSs to study the influence of ABS phase composition on the activity of lysozyme. The functional studies were attained for seven of the biphasic systems listed in Table 1, containing a fluorinated IL ([C_2_C_1_Im][C_4_F_9_SO_3_]), a mere fluoro-containing IL ([C_4_C_1_Im][CF_3_SO_3_]), two known globular protein stabilizers ([$N_{1112(\mathrm{OH})}$][H_2_PO_4_] and sucrose), a low-molecular-weight carbohydrate (glucose), and a high-charge-density salt (K_3_PO_4_). The activity of Lys in water is considered to be 100% and the other activities, partition Lys in ABS phases of BP#11 (30 %wt [C_4_C_1_Im][CF_3_SO_3_] + 20 %wt [$N_{1112(\mathrm{OH})}$][H_2_PO_4_]), BP#6 (30 %wt [C_4_C_1_Im][CF_3_SO_3_] + 25 %wt glucose), BP#4 (30 %wt [C_4_C_1_Im][CF_3_SO_3_] + 25 %wt sucrose), BP#3 (30 %wt [C_2_C_1_Im][C_4_F_9_SO_3_] + 2 %wt K_3_PO_4_), BP#8 (30 %wt [C_2_C_1_Im][C_4_F_9_SO_3_] + 6 %wt [$N_{1112(\mathrm{OH})}$][H_2_PO_4_]), BP#7 (30 %wt [C_2_C_1_Im][C_4_F_9_SO_3_] + 25 %wt glucose), and BP#5 (30 %wt [C_2_C_1_Im][C_4_F_9_SO_3_] + 25 %wt sucrose) (or Lys plus ABS phase-forming component; Section 2.2.1), are reported relatively to this study. The results are shown in Figure 15 following the order of the biphasic systems previously indicated: first, the results for [C_4_C_1_Im][CF_3_SO_3_] (mere fluoro-containing IL) and second, the results for [C_2_C_1_Im][C_4_F_9_SO_3_] (fluorinated IL). These functional studies show that the activity of partition lysozyme is not significantly affected by the ABS phase composition (ABS phase-forming component, %wt in Table 1 and mM in Table S27 of Supplementary Materials). Even for the FIL-based ABSs combined with the high-charge density K_3_PO_4_ (BP#3), only a slight decrease, down to ≈75%, is verified in the FIL-rich phase (Lys-enriched phase; %EE 96.25, Table S26 of Supplementary Materials). The great impact is verified in the K_3_PO_4_-rich phase of BP#3, which decreases down to ≈56% (only 3.75% of partition Lys; Table S26 of Supplementary Materials). For the biphasic systems containing known globular protein stabilizers ([$N_{1112(\mathrm{OH})}$][H_2_PO_4_] and sucrose) or low-molecular-weight carbohydrates (glucose), only the system BP#4 based on [C_4_C_1_Im][CF_3_SO_3_] (mere fluoro-containing IL) combined with sucrose presents a slighter decrease in lysozyme activity, down to ≈76% in the IL-rich phase and down to ≈87% in the sucrose-rich phase. Clearly, the functionalized FIL-based ABSs maintain or slightly increase the enzymatic activity.

**Figure 16:**
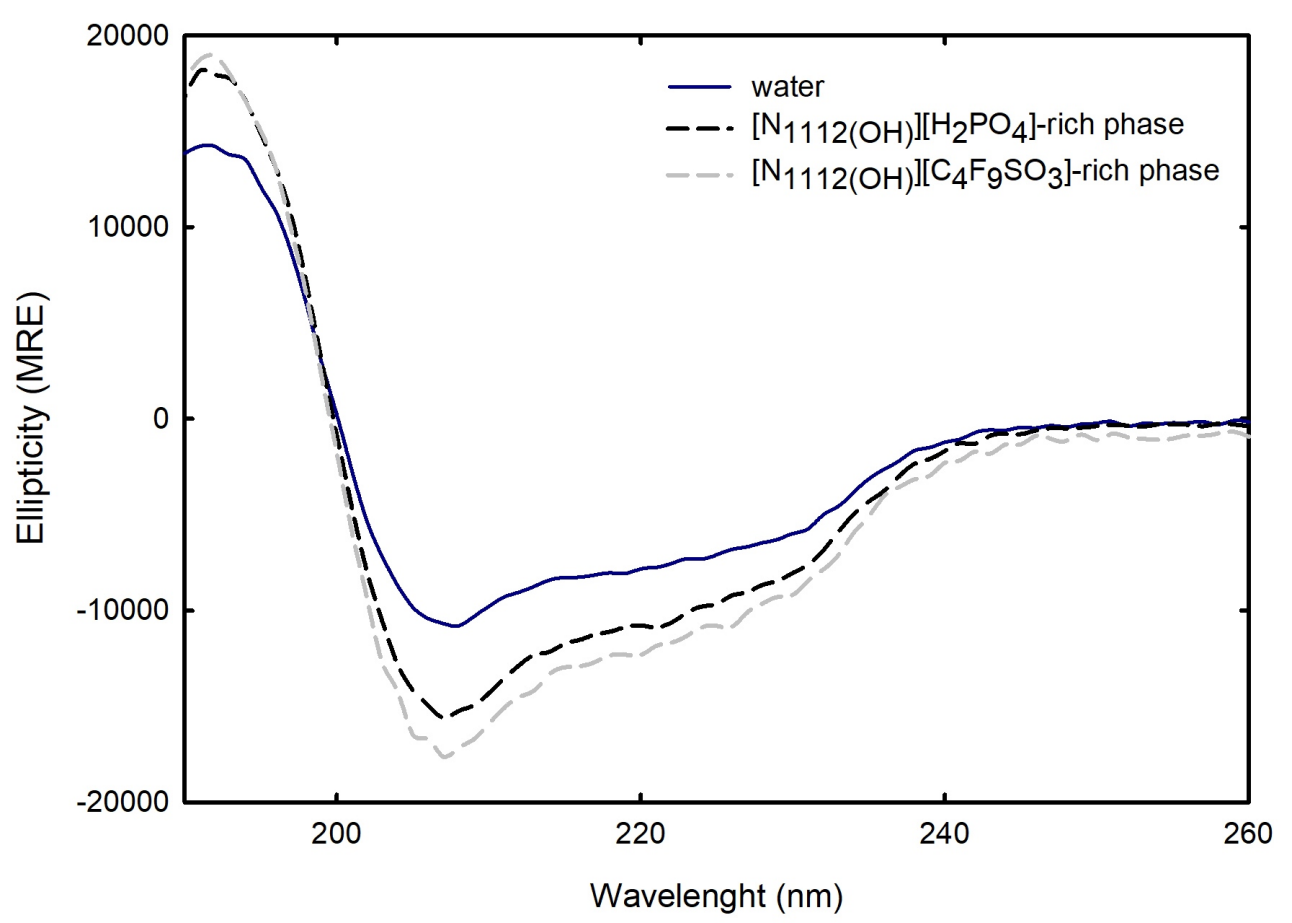
CD Spectra of Lys 1.0 mg/mL in water, Lys ≈ 2.0 mg/mL in [$N_{1112(\mathrm{OH})}$][C_4_F_9_SO_3_]-rich phase, and Lys ≈ 0.4 mg/mL in [$N_{1112(\mathrm{OH})}$][H_2_PO_4_]-rich phase of BP#12 (30 %wt [$N_{1112(\mathrm{OH})}$][C_4_F_9_SO_3_] + 30 %wt [$N_{1112(\mathrm{OH})}$][H_2_PO_4_]). All spectra were acquired at 25 °C.

For the FIL-based biphasic systems ([C_2_C_1_Im][C_4_F_9_SO_3_] and [$N_{1112(\mathrm{OH})}$][C_4_F_9_SO_3_]), since [C_2_C_1_Im][C_4_F_9_SO_3_]-rich phases are not technically feasible for CD, because of the high signal intensity of the imidazolium ring in parallel to the high concentration of [C_2_C_1_Im][C_4_F_9_SO_3_] in FIL-rich phases (the same is verified for [C_2_C_1_Im][CF_3_SO_3_]- and [C_4_C_1_Im][CF_3_SO_3_]-rich phases of the mere fluoro-containing IL-based ABSs), for example, ranging from 3007 mM (BP#3) to 11,456 mM (BP#10) [C_2_C_1_Im][C_4_F_9_SO_3_] in the [C_2_C_1_Im][C_4_F_9_SO_3_]-rich phases, spectra were only measured for the [$N_{1112(\mathrm{OH})}$][C_4_F_9_SO_3_]-based system (BP#12). As depicted in Figure 16, comparing partition Lys spectra in each biphasic phase of BP#12 (30 %wt [$N_{1112(\mathrm{OH})}$][C_4_F_9_SO_3_] + 30 %wt [$N_{1112(\mathrm{OH})}$][H_2_PO_4_]), [$N_{1112(\mathrm{OH})}$][H_2_PO_4_]-, and [$N_{1112(\mathrm{OH})}$][C_4_F_9_SO_3_]-rich phases, with the native conformation of the protein in water, no substantial alteration on the predicted structure is verified despite the high concentration of [$N_{1112(\mathrm{OH})}$][C_4_F_9_SO_3_] and [$N_{1112(\mathrm{OH})}$][H_2_PO_4_]. The estimated structural content for Lys on both [$N_{1112(\mathrm{OH})}$][H_2_PO_4_]- and [$N_{1112(\mathrm{OH})}$][C_4_F_9_SO_3_]-rich phases is slightly higher for α-helix (up to circa 40%), maintaining the 16% of β-strand and decreasing the random coil to 44%. Further, Lys is stabilized in both phases of BP#12, a [$N_{1112(\mathrm{OH})}$][C_4_F_9_SO_3_]-rich phase at concentrations of up to 6237 mM [$N_{1112(\mathrm{OH})}$][C_4_F_9_SO_3_] and 1990 mM [$N_{1112(\mathrm{OH})}$][H_2_PO_4_] and a [$N_{1112(\mathrm{OH})}$][H_2_PO_4_]-rich phase at concentrations of up to 415 mM [$N_{1112(\mathrm{OH})}$][C_4_F_9_SO_3_] and 4758 mM [$N_{1112(\mathrm{OH})}$][H_2_PO_4_] Table S27 of Supplementary Materials).

DSC was used to probe Lys’s stability in the phases of eight biphasic systems summarized in Table S26 of Supplementary Materials. The systems based on the two low-molecular-weight carbohydrates (sucrose and glucose), in combination with the [C_4_C_1_Im][CF_3_SO_3_] mere fluoro-containing IL and [C_2_C_1_Im][C_4_F_9_SO_3_] FIL (BP#4, BP#5, BP#6, and BP#7), and the systems based on the known globular protein stabilizer [$N_{1112(\mathrm{OH})}$][H_2_PO_4_] in combination with the [C_4_C_1_Im][CF_3_SO_3_] mere fluoro-containing IL and [C_2_C_1_Im][C_4_F_9_SO_3_] FIL (BP#8, BP#10, BP#11, and BP#12). The effect of each ABS phase composition (ABS phase-forming component, %wt in Table 1 and mM Table S27 of Supplementary Materials) on Lys’s $T_{m}$ and protein stabilization is addressed in Table S28 of Supplementary Materials. Due to phase composition (sucrose- and glucose-rich phases of BP#4 and BP#6, respectively, [C_2_C_1_Im][C_4_F_9_SO_3_]-rich phase of BP#10, [C_4_C_1_Im][CF_3_SO_3_]-rich phase of BP#11 and [$N_{1112(\mathrm{OH})}$][C_4_F_9_SO_3_]-rich phase of BP#12) or a limited amount of partition lysozyme (Lys-enriched phase is the counterpart phase; %EE for BP#5, BP#7, and BP#8 summarized in Table S26 of Supplementary Materials, it is not technically feasible to run a DSC of the specified phases. Lys’s $T_{m}$, the temperature at which the folded and unfolded states of the protein happen at equilibrium, for the detailed ABS phases are summarized in Table S28 of Supplementary Materials. For comparison, a set of representative normalized DSC curves of 0.5 mg/mL, 1.0 mg/mL, and 3.0 mg/mL Lys in water (concentration range of partition Lys in the considered biphasic phases) were acquired, and the $T_{m}$s are also listed. Lys’s $T_{m}$ values for the [C_4_C_1_Im][CF_3_SO_3_]- and [C_2_C_1_Im][C_4_F_9_SO_3_]-rich phases of biphasic systems combined with sucrose and glucose showed a significant decrease. These results are in opposition to the functional experiments (Figure 15), where lysozyme activity is maintained or slightly increased. A similar result is observed for [C_2_C_1_Im][C_4_F_9_SO_3_]-rich phase of BP#8, where no $T_{m}$ was detected in the DSC run (%EE 98.35; Table S26 of Supplementary Materials). The results for FIL-based systems are indicative of the formation of complexes between Lys and the FIL [C_2_C_1_Im][C_4_F_9_SO_3_] aggregates, which has been demonstrated in previous works [19,24,25,26,28,72,78]. In those works, the formation of complexes between FIL aggregates and proteins, namely Lys, BSA, and IFN-α2b, allows, upon centrifugation, to extract the complexed protein from the solution [19,24,25,26,72]. So, we analyzed the Lys precipitated from the [C_2_C_1_Im][C_4_F_9_SO_3_]-rich phases (BP#5, BP#7, and BP#8) and resuspended in water. The normalized DSC curves for ≈1.0 mg/mL resuspended Lys in water (Lys precipitated from [C_2_C_1_Im][C_4_F_9_SO_3_]-rich phase of BP#5, BP#7, and BP#8, and resuspended in water) and 1.0 mg/mL BSA in water were obtained, which allowed to determine a protein $T_{m}$ of 69.04 ± 0.005 °C (BP#5), 68.43 ± 0.004 °C (BP#7), 69.15 ± 0.013 °C (BP#8), and 73.71 ± 0.004 °C, respectively. The $T_{m}$ values are outlined in Table S28 of Supplementary Materials, indicating that partition Lys’s $T_{m}$ is maintained and that the [C_2_C_1_Im][C_4_F_9_SO_3_]-rich phase is a stabilizing medium. Similar results are verified for the [C_2_C_1_Im][C_4_F_9_SO_3_]-based BP#10 and BP#12 systems from the direct $T_{m}$ measured in the [$N_{1112(\mathrm{OH})}$][H_2_PO_4_]-rich phase. Which allowed to determine a protein $T_{m}$ of 76.31 ± 0.005 °C (1.0 mg/mL Lys, BP#10) and 66.70 ± 0.008 °C (0.5 mg/mL Lys, BP#12). For the system based on [C_4_C_1_Im][CF_3_SO_3_] (mere fluoro-containing IL), which also allows the direct $T_{m}$ measured in the [$N_{1112(\mathrm{OH})}$][H_2_PO_4_]-rich phase (BP#11), a significant decrease in Lys’s $T_{m}$ is verified ($T_{m}$ of 54.89 ± 0.012 °C for ≈1.0 mg/mL in the [$N_{1112(\mathrm{OH})}$][H_2_PO_4_]-rich phase vs. 73.71 ± 0.004 °C for 1.0 mg/mL in water).

#### 2.3.3. Lys and BSA Simultaneous Partition

Comparing the Lys extraction efficiency (%EE) depicted in Table S26 of Supplementary Materials and illustrated in Figures S18–S29 of Supplementary Materials (individual partition studies in Section 2.3.1), with the bovine serum albumin (BSA) %EE from the individual partition of BSA analyzed in other work by the authors [19], indicates that the two globular proteins, Lys and BSA, behave differently depending on the developed functionalized ABSs. Since both Lys and BSA are charged molecules, their partition behavior can be impacted by the ABS phases pH [70]. The isoelectric points of Lys and BSA are around 11.0 [58] and 4.7 [79], respectively. Both proteins are positively charged under acidic conditions, and BSA is negatively charged at neutral pH. Lysozyme is a basic globular glycoprotein that has a molecular weight of 14 kDa, dimensions of about 27.8 Å × 11.8 Å × 11.8 Å [58], and a solvent-accessible surface area (SASA) of circa 7000 Å^2^ [80]. BSA is an acidic, non-glycosylated, multidomain globular protein with a molecular weight of 66 kDa, dimensions of about 140 Å × 40 Å × 40 Å [81], a SASA of approximately 30,000 Å^2^ [80], and the surface is more hydrophilic than hydrophobic [82]. Furthermore, globular proteins such as Lys (basic) and BSA (acidic) have distinct interaction behaviors in the presence of monovalent and divalent ions due to the different pI values [83].

The partition behavior of Lys and BSA is different for the systems with K_3_PO_4_ (BP#1 and BP#3) and sucrose (BP#4 and BP#5). In the K_3_PO_4_-based systems, both phases have higher pH values, and both Lys and BSA are negatively charged. The Lys-enriched phases are the ionic-liquid-rich phases ([C_2_C_1_Im][CF_3_SO_3_] and [C_2_C_1_Im][C_4_F_9_SO_3_]), and the BSA-enriched phases are the IL-rich phase for the system with mere fluoro-containing IL (BP#1) and the K_3_PO_4_-rich phase for the system with fluorinated IL (BP#3). For the sucrose-based systems, Lys is positively charged in both systems, and BSA is near the isoelectric point in BP#4 and negatively charged in BP#5. The Lys-enriched phase is the sucrose-rich phase for the system with mere fluoro-containing IL ([C_4_C_1_Im][CF_3_SO_3_]; BP#4) and the FIL-rich phase for the system with fluorinated IL ([C_2_C_1_Im][C_4_F_9_SO_3_]; BP#5). In opposition, the BSA-enriched phase is the IL-rich phase for the system with mere fluoro-containing IL ([C_4_C_1_Im][CF_3_SO_3_]; BP#4) and the sucrose-rich phase for the system with fluorinated IL ([C_2_C_1_Im][C_4_F_9_SO_3_]; BP#5). For the [$N_{1112(\mathrm{OH})}$][H_2_PO_4_]-based systems BP#8, BP#9, BP#10, and BP#12, Lys is positively charged in all systems, and the total BSA charge depends on [$N_{1112(\mathrm{OH})}$][H_2_PO_4_] concentration. Up to 10 %wt [$N_{1112(\mathrm{OH})}$][H_2_PO_4_] (BP#8 and BP#9) BSA is positively charged, and, for higher concentrations, 20%wt [$N_{1112(\mathrm{OH})}$][H_2_PO_4_] (BP#10) and 30%wt [$N_{1112(\mathrm{OH})}$][H_2_PO_4_] (BP#12), the pH of both phases is near the isoelectric point of BSA. The partition behavior of both proteins is the same for the systems based on [C_2_C_1_Im][C_4_F_9_SO_3_] (BP#8, BP#9, and BP#10). Up to 10 %wt [$N_{1112(\mathrm{OH})}$][H_2_PO_4_] the Lys- and BSA-enriched phase is the FIL-rich phase (BP#8 and BP#9). For higher [$N_{1112(\mathrm{OH})}$][H_2_PO_4_] concentrations (20 %wt; BP#10), Lys and BSA partition into the [$N_{1112(\mathrm{OH})}$][H_2_PO_4_]-rich phase. The partition behavior of Lys and BSA is opposite in the system based on [$N_{1112(\mathrm{OH})}$][C_4_F_9_SO_3_] (BP#12), with the positively charged Lys partitioning to the [$N_{1112(\mathrm{OH})}$][C_4_F_9_SO_3_]-rich phase and the neutral BSA partitioning to the [$N_{1112(\mathrm{OH})}$][H_2_PO_4_]-rich phase. Lys hydrophobicity is higher than BSA [84], and hydrophobic interactions between Lys molecules are stronger than those between BSA molecules [85]. These two features are advantageous to promote the interaction with the anionic counterparts of the FILs, which also have a strong hydrophobic nature.

**Figure 17:**
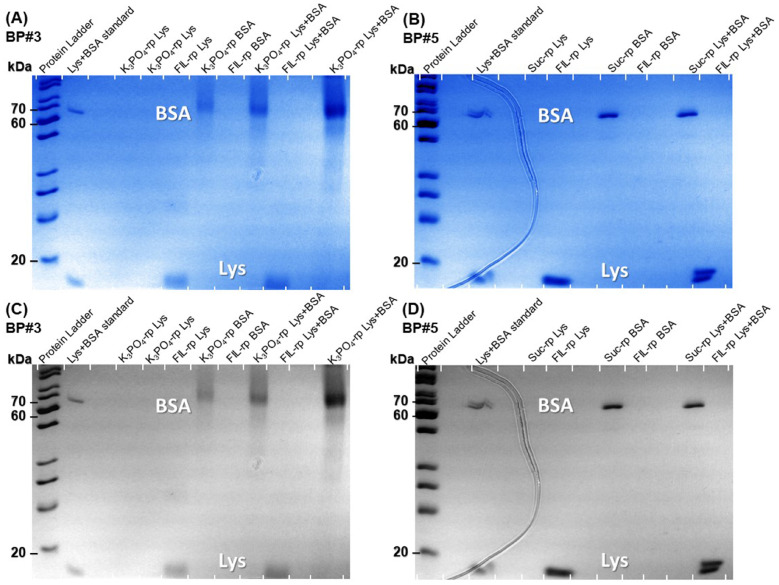
SDS-PAGE analysis of the standard protein marker (protein ladder), Lys and BSA standards, samples from FIL-rich phase and non-FIL-rich phase (K_3_PO_4_-rich phase and sucrose-phase) from individual partitions (lane label, Lys or BSA) and simultaneous partition (lane label, Lys + BSA) stained with Coomassie blue. (**A**) BP#3, 30 %wt [C_2_C_1_Im][C_4_F_9_SO_3_] + 2 %wt K_3_PO_4_. (**B**) BP#5, 30 %wt [C_2_C_1_Im][C_4_F_9_SO_3_] + 25 %wt sucrose. The monochromatic version is displayed in (**C**) and (**D**), respectively.

The Lys and BSA extraction efficiency (%EE) attained from the individual partition studies, detailed in Table S26 of Supplementary Materials and Figure 11, Figure 12 and Figure 13 for Lys and, in a previous work by the authors for BSA [19], suggest that the systems 30 %wt [C_2_C_1_Im][C_4_F_9_SO_3_] + 2 %wt K_3_PO_4_ (BP#3), 30 %wt [C_2_C_1_Im][C_4_F_9_SO_3_] + 25 %wt sucrose (BP#5), and 30 %wt [$N_{1112(\mathrm{OH})}$][C_4_F_9_SO_3_] + 30 %wt [$N_{1112(\mathrm{OH})}$][H_2_PO_4_] (BP#12) allow a single-step simultaneous purification of Lys and BSA. All three biphasic systems are FIL-based ([C_2_C_1_Im][C_4_F_9_SO_3_] and [$N_{1112(\mathrm{OH})}$][C_4_F_9_SO_3_]), and, for those systems, the Lys-enriched phase is the FIL-rich phase. The rich-aggregation behavior of the [C_2_C_1_Im][C_4_F_9_SO_3_] and [$N_{1112(\mathrm{OH})}$][C_4_F_9_SO_3_] FILs in aqueous media [21,26,28,78] and the reported protein–protein interactions [85,86] and complex formation [87,88] between Lys and BSA can modify the individual partition behavior of both proteins. The FIL-based biphasic systems BP#3 and BP#5 were selected as model systems to assess the single-step simultaneous purification of Lys and BSA. For these systems, the %EE of Lys is greater than 95% and the %EE of BSA ranges in a 0–5% interval, allowing a higher purification factor. Accordingly, both phases of these two FIL-based ABSs (BP#3 and BP#5) in the simultaneous partition of Lys and BSA were analyzed using the SDS-PAGE method to assess the purity of Lys and BSA. Figure 17 shows the SDS-PAGE analysis for both biphasic systems (a monochromatic version is also displayed), displaying the SDS-PAGE profile of a standard protein marker (protein ladder, lane 1), Lys and BSA standard, FIL-rich phase and non-FIL-rich phase ([C_2_C_1_Im][C_4_F_9_SO_3_]-rich phase and K_3_PO_4_-rich phase), sample of individual partition (lane label, Lys, or BSA), and FIL-rich phase, and non-FIL-rich phase sample of simultaneous partition (lane label, Lys+BSA). A detailed identification of all lanes in Figure 17A,B is summarized in Table S29 of Supplementary Materials. An equivalent sample of each ABS phase having approximately 0.5 μg per lane of the enriched protein was analyzed for the biphasic systems BP#3 and BP#5. The SDS-PAGE profiles of the FIL-rich phases of the two biphasic systems for the simultaneous partition of Lys and BSA did not detect any BSA (lane label Lys+BSA). In the non-FIL-rich phase counterparts, the K_3_PO_4_-rich phase (BP#3) and sucrose-rich phase (BP#5), no Lys was detected with the SDS-PAGE profiles of the phases (lane label, Lys+BSA). The SDS-PAGE analysis shows high purity for Lys and BSA. The FIL-rich phase of the ABSs BP#3 and BP#5 exhibited only one band after 20 kDa (protein ladder), indicating that Lys (14 kDa) was successfully purified to the FIL-rich phase. The non-FIL-rich phase counterparts exhibited only one band between 70 kDa and 60 kDa (protein ladder), indicating that BSA (66 kDa) was simultaneously purified to the opposite phase of the optimized FIL-based ABS. The SDS-PAGE analysis confirmed the successful single-step simultaneous purification of Lys and BSA. Additionally, the SDS-PAGE profiles depicted in Figure 17 for Lys and BSA individual and simultaneous partitions corroborate the partition behavior (%EE) of Lys and BSA, quantified through UV-VIS, MICRO BCA, BCA, and Bradford protein assay for Lys (Table S26 of Supplementary Materials), and quantified through MICRO BCA, BCA, and Bradford protein assay for BSA [19]. All the %EE results for Lys and BSA are in agreement with the implemented experimental SDS-PAGE procedure (Section 3.10), including the determined detection limit of approximately 15 ng.

#### 2.3.4. Lysozyme Interaction with ABS Phase-Forming Components

Microscale thermophoresis (MST) allows a quantitative measurement of binding affinities regardless of the size and physical properties of the target molecules as a result of fluorescence changes due to the motion of fluorescent molecules along local temperature gradients in reduced volumes, providing a highly sensitive characterization of biomolecular interactions. Accordingly, it has been used in binding assays of biomolecules (DNA, enzymes, and proteins) with small molecules of interest (substarcts, ligands, and liposomes, among others). Protein–ligand interactions induce modifications in proteins’ size, charge, and solvation energy, which are detected by thermophoresis [89]. Even if the biding produces reduced structural modifications, they can be detected by MST due to the variations in the solvation entropy of the fluorescent labeled protein. These changes can be used for plotting the normalized fluorescence (Fnorm) of the labeled protein vs. the logarithm of the concentration of ligand, and fitting a binding curve with the models provided by the software to estimate the equilibrium dissociation constant ($K_{d}$) [90,91]. The strength of protein–ligand binding can be characterized through affinity, inversely proportional to the $K_{d}$. A lower $K_{d}$ value indicates stronger binding and higher affinity between the biomolecule and the ligand [92]. MST allows a rapid and quantitative characterization of interactions based on the thermophoretic behavior of biomolecules and their sensitivity to non-covalent binding [93]. MST, a quite novel technique, was already used to determine the binding affinities of interferons [94,95] and other proteins [66,91,96] with different types of ligands. In recent research comparing two SARS-CoV-2 spike variants that bind human cell lines with differing affinities, MST has been shown to be helpful and widely applicable, making a significant contribution to the public health challenge during the COVID-19 pandemic due to the specificity and sensitivity of this technique [97,98]. Further, the authors also applied the MST assay to infer the interactions between IFN-*α*2b [19,28] and BSA [19] with ionic liquids.

In this work, Lys was labeled with fluorescein-isothiocyanate (FITC) using a commercial labeling kit (Section 3). Usually, in the labeling process, the position of dye is statistically distributed, and one amine per protein is labeled. Therefore, given the typical number of lysine residues in proteins, the risk of the label impairing the binding is minimal [66,91]. The four ABS phase-forming components, [$N_{1112(\mathrm{OH})}$][H_2_PO_4_] (globular protein stabilizer), [C_2_C_1_Im][C_4_F_9_SO_3_] and [$N_{1112(\mathrm{OH})}$][C_4_F_9_SO_3_] (FILs), and [C_4_C_1_Im][CF_3_SO_3_] (mere fluoro-containing IL), were used as ligands to study the binding with fluorescently labeled Lys (FITC-Lys). The MST assays were performed as described in Section 3.11. The solutions were prepared with a concentration of Lys fixed at 0.41 μM in water. In each run of MST, a maximum concentration of ligand was selected and consecutively diluted in sixteen capillaries. From the CAC values of both FILs (Table S23 of Supplementary Materials), five different maximum concentrations for [C_2_C_1_Im][C_4_F_9_SO_3_] and four different maximum concentrations for [$N_{1112(\mathrm{OH})}$][C_4_F_9_SO_3_] were selected to cover the range below the formation of aggregates (<first CAC) and to study the different CACs (four CACs for [C_2_C_1_Im][C_4_F_9_SO_3_] and three CACs for [$N_{1112(\mathrm{OH})}$][C_4_F_9_SO_3_]) [21]. Due to the experimental specifications of the MST assay (Section 3.11), only the aggregation phenomenon of the first CAC can be analyzed individually. For the MST runs with the concentration of the first capillary above the second CAC, the determined $K_{d}$ values for the runs that yield binding are influenced by the several aggregates, which make it impossible to attribute the $K_{d}$ value to a specific CAC. The determined $K_{d}$ comprises the influence of the distinct aggregates, allowing a qualitative analysis of the impact of the various aggregates on the binding affinity. For the mere fluoro-containing IL ([C_4_C_1_Im][CF_3_SO_3_]) and known protein stabilizer ([$N_{1112(\mathrm{OH})}$][H_2_PO_4_]), ABS phase-forming components without self-aggregation behavior, a concentration of 100 mM was analyzed (fourth CAC [C_2_C_1_Im][C_4_F_9_SO_3_]; highest CAC order of the analyzed FILs).

For all the studied systems (four ligands: FILs, [C_2_C_1_Im][C_4_F_9_SO_3_] and [$N_{1112(\mathrm{OH})}$][C_4_F_9_SO_3_]; mere fluoro-containing IL, [C_4_C_1_Im][CF_3_SO_3_]; globular protein stabilizer, [$N_{1112(\mathrm{OH})}$][H_2_PO_4_]), response curves were captured as titration series over an increasing concentration of ligand and normalized to their initial fluorescence in equilibrium. The eight assays are depicted as normalized fluorescence (Fnorm) in Figures S30–S33 of Supplementary Materials). For the concentration range below the first CAC for both FILs, where only FIL monomers are present in aqueous solution, no binding was detected with Lys (Figures S30A and S31A of Supplementary Materials). The same result was attained for the ABS phase-forming components without self-aggregation behavior, [C_4_C_1_Im][CF_3_SO_3_] and [$N_{1112(\mathrm{OH})}$][H_2_PO_4_] for the concentration range [100 mM; 3.05 × 10^−3^] analyzed (Figures S32 and S33 of Supplementary Materials). These results are summarized in Table 2. Similar results were observed for BSA (a globular protein like Lys) and IFN-α2b labeled with the same fluorescent tag (FITC-BSA and FITC-IFN-α2b) at the same concentration (0.41 μM) [19], and for IFN-α2b at a different concentration (2.7 μM) labeled with a different fluorescent tag (Alexa Fluor 555) [28].

**Figure 18:**
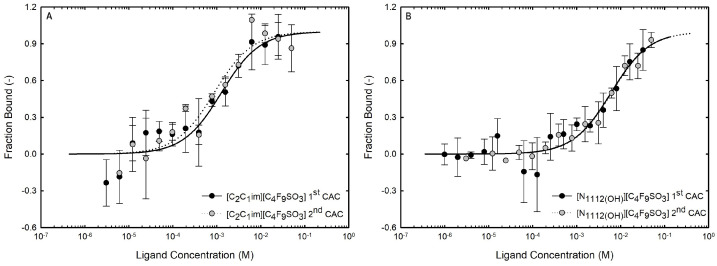
MST binding curves of 0.41 μM FITC-Lys with (**A**) [C_2_C_1_Im][C_4_F_9_SO_3_], and (**B**) [$N_{1112(\mathrm{OH})}$][C_4_F_9_SO_3_] at different starting concentrations. For (**A**) [C_2_C_1_Im][C_4_F_9_SO_3_], concentration ranges from [14.4, 34.48[ mM ($K_{d}$ = 1.27 ± 0.64 mM) to [34.48, 76.54[ mM ($K_{d}$ = 0.87 ± 0.36 mM). For (**B**) [$N_{1112(\mathrm{OH})}$][C_4_F_9_SO_3_], concentration ranges from [16.02, 35.17[ mM ($K_{d}$ = 6.05 ± 1.67 mM) to [35.17, 185.65[ mM ($K_{d}$ = 6.25 ± 1.47 mM). The fraction bound is plotted as a function of ligand concentration, and curves are fitted using the kd method of the NanoTemper Analysis software (TA NanoAnalyse Data Analysis v3.12.0). All the samples were measured in water at 25 °C. Error bars represent the standard deviation of 3 measurements. The determined $K_{d}$s are summarized in Table 2.

For [C_2_C_1_Im][C_4_F_9_SO_3_] (Figure S30B,C of Supplementary Materials) and [$N_{1112(\mathrm{OH})}$][C_4_F_9_SO_3_] (Figure S31B,C of Supplementary Materials)) at concentrations above the first CAC, binding between Lys and each FIL was observed for different stages of aggregation. Lys was only titrated up to 50 mM of [C_2_C_1_Im][C_4_F_9_SO_3_] and [$N_{1112(\mathrm{OH})}$][C_4_F_9_SO_3_] due to experimental limitations. The MST equipment software (MO.Affinity Analysis v2.3), based on a set of quality checks, does not consider capillaries with ligand concentrations higher than 50 mM due to fluorescence inhomogeneity or ligand-induced fluorescence change. This constrains the analysis of binding affinity between Lys and both FILs to the first CAC and second CAC aggregates of [C_2_C_1_Im][C_4_F_9_SO_3_] and [$N_{1112(\mathrm{OH})}$][C_4_F_9_SO_3_] (Table S23 of Supplementary Materials). In previous works, the authors already determined the binding affinities of BSA and IFN-α2b with these two FILs above the highest CAC (fourth CAC for [C_2_C_1_Im][C_4_F_9_SO_3_] and third CAC for [$N_{1112(\mathrm{OH})}$][C_4_F_9_SO_3_]; Table S23 of Supplementary Materials). The dose response curves of Lys binding with [C_2_C_1_Im][C_4_F_9_SO_3_] and [$N_{1112(\mathrm{OH})}$][C_4_F_9_SO_3_] that were fitted for $K_{d}$ determination are depicted in Figure 18, plotting fraction bound vs. ligand concentration. The values of $K_{d}$ for both ligands ([C_2_C_1_Im][C_4_F_9_SO_3_] and [$N_{1112(\mathrm{OH})}$][C_4_F_9_SO_3_]) are listed in Table 2. Analyzing the results for both FILs, the Lys bindings with [C_2_C_1_Im][C_4_F_9_SO_3_] and [$N_{1112(\mathrm{OH})}$][C_4_F_9_SO_3_] are distinct. With [$N_{1112(\mathrm{OH})}$][C_4_F_9_SO_3_], the $K_{d}$ increases with the increasing order of the CAC, indicating a weaker binding and lower affinity between Lys and [$N_{1112(\mathrm{OH})}$][C_4_F_9_SO_3_]. The aggregates formed at a lower concentration of [$N_{1112(\mathrm{OH})}$][C_4_F_9_SO_3_] have a higher affinity for Lys. Similar results were obtained for BSA [19] and IFN-α2b [19,28] with [$N_{1112(\mathrm{OH})}$][C_4_F_9_SO_3_]. The aggregates formed at lower concentrations of [$N_{1112(\mathrm{OH})}$][C_4_F_9_SO_3_] have higher affinity for Lys, BSA, and IFN-α2b. Furthermore, the aggregates formed at lower concentrations of [C_2_C_1_Im][C_4_F_9_SO_3_] have a higher affinity for BSA [19] and IFN-α2b [19,28]. Differently, the aggregates formed at a higher concentration of [C_2_C_1_Im][C_4_F_9_SO_3_] have a higher affinity for Lys. In the Lys binding with [C_2_C_1_Im][C_4_F_9_SO_3_], the $K_{d}$ decreases with the increasing order of the CAC, indicating a stronger binding and higher affinity between Lys and [C_2_C_1_Im][C_4_F_9_SO_3_].

The trend is verified for Lys; a decreased $K_{d}$ is determined for the assay that comprises a higher concentration of [C_2_C_1_Im][C_4_F_9_SO_3_] (i.e., a higher order of the CAC), indicating that the aggregates formed at higher concentration of FIL have a higher affinity for Lys, as verified in Figure 18A by the deviation of the sigmoidal dose response to the left [19,28,66]. The Lys binding with [C_2_C_1_Im][C_4_F_9_SO_3_] and [$N_{1112(\mathrm{OH})}$][C_4_F_9_SO_3_] shows that the [C_2_C_1_Im]^+^ cation has more binding affinity with Lys than the [$N_{1112(\mathrm{OH})}$]^+^ cation, demonstrated by the lower $K_{d}$ values and the left shift of the sigmoidal for both the first CAC and second CAC with [C_2_C_1_Im][C_4_F_9_SO_3_] (Figure S34 of Supplementary Materials). Otherwise, for BSA and IFN-α2b the higher affinity is attained with the [$N_{1112(\mathrm{OH})}$]^+^ cation [19,28]. The binding between BSA and [C_2_C_1_Im][C_4_F_9_SO_3_] was previously studied by some of the authors using isothermal titration calorimetry (ITC) [26], a fluorescence-labeling-free technique that rules out potential interference of protein labeling, supporting the results attained with MST [19]. The ITC results demonstrate that BSA interacts with [C_2_C_1_Im][C_4_F_9_SO_3_], even FIL monomers, and it is encapsulated by FIL aggregates while its stability is improved. The MST assays with Lys and the ABS phase-forming components, [C_2_C_1_Im][C_4_F_9_SO_3_] and [$N_{1112(\mathrm{OH})}$][C_4_F_9_SO_3_] (FILs), [C_4_C_1_Im][CF_3_SO_3_] (mere fluoro-containing IL), and the known globular protein stabilizer [$N_{1112(\mathrm{OH})}$][H_2_PO_4_], used as ligands, have provided valuable insights about the binding affinity between ABS phase-forming components and protein partition. The strong binding between Lys with the aggregates of both studied FILs and the different affinity of the distinct aggregates support the results previously discussed about the functionalized FIL-based ABSs and the selective partition of Lys and BSA.

## 3. Materials and Methods

### 3.1. Reagents

Lyophilized lysozyme from chicken egg white, ≥90% purity, and Micrococcus Lysodeikticus lyophilized cell substrate were both acquired from Millipore (Darmstadt, Germany) Choline ((2-hydroxyethyl)trimethylammonium) dihydrogen phosphate >98 %wt, [$N_{1112(\mathrm{OH})}$][H_2_PO_4_], phosphate buffer (PBS), D-(+)-Glucose (C_6_H_1_2O_6_) ≥99.5%, sucrose (C_1_2H_2_2O_1_1) ≥ 99.5%, and potassium phosphate tribasic (K_3_PO_4_) reagent grade ≥98% salt were purchased from Sigma-Aldrich. The Pierce^*TM*^ FITC antibody labeling kit was purchased from Thermo Scientific (Waltham, MA, USA). ILs and FILs were all supplied by IoLiTec GmbH (Heilbronn, Germany), and their respective purities and chemical structures are presented in Table S1 of Supplementary Materials, as well as each acronym used. To reduce the water contents and volatile chemical impurities, all ILs and FILs were further dried at 40 °C and 4 Pa vacuum pressure under constant stirring for at least 48 h preceding their use. Using a Metrohm 831 Karl Fischer Coulometer (Metrohm AG, Herisau, Switzerland), we found a water content below 100 ppm after this procedure. The purity of the used ILs was checked by ^1^H nuclear magnetic resonance (NMR), while the purity of the FILs was checked further by ^19^F as long as ^1^H NMR, using a Bruker Avance (Bruker UK Ltd., Coventry, UK) at 400 MHz. All experiments used double-distilled water, which had passed through a reverse osmosis system and was further treated with Milli-Q Plus 185 water purification equipment (Millipore, Darmstadt, Germany).

### 3.2. ABS Phase Diagrams, Merchuck Fitting, and Tie-Lines

Conventional ILs, mere fluoro-containing ILs, and flourinated ILs were evaluated for their potential for ABSs using the cloud point titration method with distinct salting-out agents: K_3_PO_4_, sucrose, D-(+)-glucose, and [$N_{1112(OH)}$][H_2_PO_4_]. Binodal curves were determined in a temperature-controlled cell at 25 °C (±0.1 °C) under constant stirring and atmospheric pressure. For each system tested, an aqueous solution of the salting-out agent and Mili-Q water were alternately dropwisely added to an aqueous solution of IL or FIL until detection of a cloud solution (indicating a biphasic region) or a clear solution (monophasic region). Aqueous IL and FIL solutions with concentrations ranging from 50 up to 90 %wt were used. The ternary system compositions were gravimetrically determined within ±10^−4^ g of uncertainty, and the experimental binodal curve data were fitted using Merchuck’s system Equation (1) [44], where *Y* and *X* represent the mass fraction percentage of the IL/FIL and non-IL/non-FIL or salt/carbohydrate, respectively, and A, B, and C are the fitting parameters by least squares regression.

(1) $$Y=A^{\left[\left(B\times X^{0.5}\right)-C\times X^{3}\right]}$$

The tie-lines were determined by the gravimetric method as well [44]. Each TL was determined using the system of equations shown below,

(2) $$Y_{\mathrm{IL}/\mathrm{FIL}}=A^{\left[\left(B\times X_{\mathrm{IL}/\mathrm{FIL}}^{0.5}\right)-C\times X_{\mathrm{IL}/\mathrm{FIL}}^{3}\right]}$$

(3) $$Y_{\mathrm{non}-\mathrm{IL}/\mathrm{non}-\mathrm{FIL}}=A^{\left[\left(B\times X_{\mathrm{salt}}^{0.5}\right)-C\times X_{\mathrm{non}-\mathrm{IL}/\mathrm{non}-\mathrm{FIL}}^{3}\right]}$$

(4) $$Y_{\mathrm{IL}/\mathrm{FIL}}=\frac{Y_{m}}{\alpha }-\frac{1-\alpha }{\alpha }\times Y_{\mathrm{non}-\mathrm{IL}/\mathrm{non}-\mathrm{FIL}}$$

(5) $$Y_{\mathrm{non}-\mathrm{IL}/\mathrm{non}-\mathrm{FIL}}=\frac{X_{m}}{\alpha }-\frac{1-\alpha }{\alpha }\times X_{\mathrm{non}-\mathrm{IL}/\mathrm{non}-\mathrm{FIL}}$$

where Y, X, and m are, respectively, the IL/FIL and the salting-out reagent weight fraction percentages and the mixture; and α is the ratio between Il/FIL-rich phase mass and the total mass of the mixture. A, B, and C are the fitted constants determined by Equation (1).

### 3.3. Phase Characterization and Partition

Before conducting partition assays, a selection of biphasic points was prepared, and their phases were identified and characterized. Each mixture was vigorously stirred and centrifuged at 25 °C with 10,000 rpm for 3 min to ensure complete phase separation and equilibrium at the stationary state between the coexisting phases. Each BP system was gently separated, the volume of each phase was measured, and a comprehensive characterization of both phases was conducted. The pH was measured using a Mettler Toledo pH meter. Karl Fisher coulometric titration was used to determine the water content in each phase. Density was measured on the Anton Paar SVM 3000 (Anton Paar GmbH, Graz, Austria) with an uncertainty of ±0.0005 g/cm^3^. To identify each phase main component, ^1^H and ^19^F NMR spectra were acquired for each BP system phase using a Bruker Avance 400 (Bruker UK Ltd., Coventry, UK) at 400 MHz with deuterated water solvent. Partition assays were then prepared employing the same procedure, albeit with 1 mg/mL of Lys protein concentration for a total mass of 2 g.

### 3.4. Protein Quantification

UV-Vis spectrophotometry was performed for protein detection on each phase for all the studied systems using a double-beam UV6300PL spectrophotometer at 280 nm. Aqueous solutions of 0.1% and 1% (*w/w*) K_3_PO_4_ were used for calibration curves, and 0.4 mL and 4.0 mL pathlength cuvettes were used. We diluted the phases at the minimum possible concentration to eliminate phase component interference and analyzed a 280 nm peak profile. The extraction efficiency was determined by the percentage ratio between the mass of protein in the IL/FIL-aqueous-rich phase (m Lys IL/FIL-rp) and the total mass of protein in the mixture as expressed by Equation (6). At least three individual experiments were carried out in order to determine %EE. Two different bicinchoninic acid (BCA) methods, Pierce^*TM*^ BCA and MICRO BCA^*TM*^ Protein Assay KIT from Thermo Scientific, were performed for protein concentration detection on FIL-rich phases and FIL/IL-rich phases, respectively. Microplates were sealed with SecureSeal Thermal Adhesive Sealing Film (Thermo Scientific, Carlsbad, CA, USA) and mixed on a plate shaker for 30 s prior to incubation at 37 °C for 30 min in BCA or 2 h in MICRO BCA assays. Absorbance at 562 nm was measured on a Thermo Scientific Multiskan Go. A ready-to-use Coomassie Plus^*TM*^ protein assay reagent from Thermo Scientific was used for total protein concentration through the Bradford Assay. The microplate was mixed for 30 s and then incubated at room temperature for 10 min. Absorbance at 595 nm was measured on a Thermo Scientific Multiskan Go. The extraction efficiency was determined by the percentage ratio between the mass of protein in the IL/FIL-aqueous-rich phase (m Lys IL/FIL-rp) and the total mass of protein in the mixture. At least three individual experiments were carried out in order to determine EE%, which was calculated using Equation (6),

(6) $$\mathrm{EE}\%=\frac{m_{\mathrm{protein}\text{FIL-rp}}}{m_{\mathrm{protein}\text{FIL-rp}}+m_{\mathrm{protein}\text{non-FIL-rp}}}.$$

### 3.5. Lysozyme Enzymatic Activity

We tested how well lysozyme broke down the cell wall of Micrococcus lysodeikticus in the presence of two different types of water from all the systems we looked at. As previously described [24], the reaction surmises the turbidity’s changes in bacterial suspension with the decrease in absorbance at 450 nm. We performed absorbance measurements at room temperature in a Multiskan GO Thermo Fisher Scientific microplate reader using U-bottom 96-well microplates. Lysozyme solutions were prepared at 0.2 mg/mL with 5, 10, 15, 25, and 35 %wt of [C_2_C_1_Im][CF_3_SO_3_], [C_2_C_1_Im][C_4_F_9_SO_3_] and sucrose concentrations in 66 mM potassium phosphate buffer pH = 6.2. A 0.3 mg/mL substrate solution of Micrococcus lysodeikticus was prepared with the same buffer, and the prepared solutions were incubated for 30 min at 25 °C. We directly evaluated lysozyme activity in the coexisting phases using the following biphasic point systems: [C_2_C_1_Im][CF_3_SO_3_] + 10%K_3_PO_4_, 30%[C_4_C_1_Im][CF_3_SO_3_] + 20%[$N_{1112(OH)}$][H_2_PO_4_], 30%[C_4_C_1_Im][CF_3_SO_3_] + 25%Glucose, 30%[C_4_C_1_Im][CF_3_SO_3_] + 25%Sucrose, 30%[C_2_C_1_Im][C_4_F_9_SO_3_] + 2%K_3_PO_4_, 30%[C_2_C_1_Im][C_4_F_9_SO_3_] + 6%[$N_{1112(OH)}$][H_2_PO_4_], 30%[C_2_C_1_Im][C_4_F_9_SO_3_] + 25%Glucose, and 30%[C_2_C_1_Im][C_4_F_9_SO_3_] + 25%Sucrose. Lysozyme relative activity was determined considering the blank of lysozyme 0.2 mg/mL as 100% activity, and lysozyme activity was determined comparatively. We mixed 10 microliters of each tested solution with 100 microliters of substrate. For 5 min, we monitored absorbance at 30-s intervals and collected them in triplicate. We conducted at least two independent experiments, ensuring errors were within less than 10%. The linear turbidity decrease of the plotted absorbances stated lysozyme activity.

### 3.6. Turbidimetry and UV-Vis Absorbance

Both turbidimetry and UV-Vis absorbance assays were carried out at 25 °C on a double-beam UV6300PL UV-Vis spectrophotometer (VWR, Leuven, Belgium) with a pair of 10 mm path length quartz cuvettes of 400 µL sample volume. Transmittance spectra for Turbidimetry were acquired with a wavelength range from 190 to 650 nm. For absorbance measurements, all spectra were acquired with 190 to 400 nm wavelength range. A fixed Lys concentration of 0.2 mg/mL was used. Aqueous solutions of [C_4_C_1_Im][CF_3_SO_3_] and [C_2_C_1_Im][C_4_F_9_SO_3_] were analyzed with concentrations varying from 0.1 to 120 mM.

### 3.7. Intrinsic Fluorescence

Intrinsic fluorescence spectroscopy measurements of lysozyme’s tryptophan residues were carried out on a Spex Horiba Jobyin Yvon spectrofluorometer (HORIBA Jobin Yvon, France) using a 10 mm path length quartz cuvette. The excitation wavelength was set at 295 nm and the emission spectra were recorded between 300 and 500 nm at room temperature for 0.2 mg/mL lysozyme fixed concentration in the presence of aqueous solutions of [C_4_C_1_Im][CF_3_SO_3_], [C_2_C_1_Im][C_4_F_9_SO_3_], and [$N_{1112(OH)}$][H_2_PO_4_] with varying concentrations from 0.1 to 800 mM. The excitation and emission slit widths of 2 nm were used for all the measurements. Maximum fluorescence values were attained as the average of three measurements.

### 3.8. DSC

The thermal stability of lysozyme was analyzed in water, ABS phase-forming compounds aqueous solutions, and partition ABS phases. Lysozyme melting temperature ($T_{m}$) was determined in water at concentrations of 1, 2, and 3 mg/mL. All DSC measurements with aqueous solutions of [C_2_C_1_Im][C_4_F_9_SO_3_], [C_4_C_1_Im][CF_3_SO_3_], [$N_{1112(\mathrm{OH})}$][C_4_F_9_SO_3_], and [$N_{1112(\mathrm{OH})}$][H_2_PO_4_] from 0.1 to 200 mM were performed at a constant protein concentration of 1.0 mg/mL. Partition lysozyme on each phase from the prepared biphasic points as well as recovered protein from the FIL-rich phase were also addressed for a selection of studied systems listed in Table 1. We acquired thermograms using a TA^*TM*^ Nano DSC from TA Instruments. All scans were conducted in a 300 μL capillary cell through a temperature ramp from 20 to 90 °C at a heating rate of 1 °C/min and 3 atm pressure. All samples were degassed for 7 min at 20 °C, with the exception of FIL-containing samples owing to their surfactant properties. After subtracting the polynomial fit baselines, raw thermogram curve data were fitted with the TwoStateScaled model using NanoAnalyze TA Instruments software, version 3.12, to determine the protein thermal transition temperatures in each sample. During nanoDSC scans, ΔH and ΔS were obtained and utilized to determine ΔG (ΔG = ΔH − TΔS).

### 3.9. CD

Circular dichroism (CD) spectra were collected in a 10 mm quartz cuvette and a Chirascan spectropolarimeter (Applied Photophysics, Leatherhead, UK) at room temperature. All CD spectra were recorded in far-UV 190 to 260 nm at scan rates of 50 nm/min, 4 accumulations, and a response time of 3 s. Each CD experiment involved the acquisition and combination of two distinct duplicates. All CD spectra were taken at 1 mg/mL fixed Lys concentration, varying the concentration of solvent from 0.1 to 200 mM, and then translated to MRE after being expressed in terms of molar ellipticity. Spectral deconvolution was carried out with Dichroweb using the K2D algorithm [65,99].

### 3.10. SDS-PAGE

Protein samples from two exemplary competitive partition systems (BP#3 and BP#5) were evaluated using SDS-PAGE in a Thermo Fisher SureCast^*TM*^ Gel Handcast System. A mixture of 12% acrylamide resolving gel and 4% acrylamide stacking gel was used for about 40 min, with Tris-Glycine SDS Running Buffer at 125 V and 3 A. Lys and BSA protein samples were prepared by adding an equal volume of Tris-Glycine SDS Sample Buffer, followed by a 5-min incubation at 85 °C. Loaded protein samples were prepared with an optimum protein mass of 0.5 μg in each gel lane. Thermo Scientific PageRuler Unstained Protein Ladder was loaded directly into each running gel as size standards ranging from 10 to 200 kDa. After electrophoresis, we rinsed the gel with water to eliminate SDS and stained it with SimplyBlue SafeStain, gently shaking it at 50 rpm for at least 3 h. We used a staining volume large enough to fully immerse the gel in dye. To remove unspecific staining and reveal protein bands, we decolored the sample using a solution of 10% NaCl and double-distilled water. The DryEase^*TM*^ Mini-Gel Drying System was used to dehydrate each gel overnight, making them easier to store and manipulate during photography.

### 3.11. MST

Binding studies were conducted through microscale thermophoresis measurements of dye-labeled lysozyme. Lysozyme was labeled using Thermo Scientific’s Pierce^*TM*^ FITC Labeling Kit according to the manufacturer’s recommended protocol, followed by UV/VIS spectrophotometry at 494 and 280 nm to attain the degree of labeling. MST tests were conducted in a monolith NT.115 (blue/red) in accordance with the Nano Temper Technologies methodology. Medium MST power and 20% LED power (nano-blue) were set as the instrument’s default settings. For each and every MST measurement, PBS buffer was used. The concentration of FITC-Lys was fixed at 0.4 μM and measured in the presence of [C_2_C_1_Im][C_4_F_9_SO_3_], [C_4_C_1_Im][CF_3_SO_3_], [$N_{1112(OH)}$][C_4_F_9_SO_3_], and [$N_{1112(OH)}$][H_2_PO_4_] as ligand. For each ligand, a series of 16 1:1 dilutions was prepared using the same buffer, varying the starting ligand concentration from 15 to 400 mM. MST traces were recorded using the standard parameters: 5 s MST power off, 30 s MST power on, and 5 s MST power off. Three independently conducted pipetted experiments’ data were examined using NanoTemper Technologies’ MO. Affinity Analysis program, version 2.3.

## 4. Conclusions

This work discloses functionalized ABSs composed of FILs ([C_2_C_1_Im][C_4_F_9_SO_3_] and [$N_{1112(\mathrm{OH})}$][C_4_F_9_SO_3_]), mere fluoro-comtaining ILs ([C_2_C_1_Im][CF_3_SO_3_] and [C_2_C_1_Im][C_4_F_9_SO_3_]), known globular protein stabilizers (sucrose and [$N_{1112(\mathrm{OH})}$][H_2_PO_4_]), a low-molecular-weight carbohydrate (glucose), and even high-charge density salt (K_3_PO_4_). The ternary phase diagrams and tie-lines were determined at 25 °C and atmospheric pressure. The systems based on FILs, [C_4_F_9_SO_3_]-based ILs, significantly increased ABS formation ability. The functionalized ABSs developed (FILs vs. mere fluoro-containing ILs) were used to extract lysozyme, used as a protein model, contributing to expand the range of biocompatible ionic-liquid-based ABSs in the extraction and purification processes of proteins. Prior to protein partition, the ABS biphasic regions were screened in terms of protein biocompatibility, analyzing the impact of ABS phase-forming components in lysozyme by UV-VIS spectrophotometry (transmittance and absorption spectra), circular dichroism spectroscopy, fluorescence spectroscopy, differential scanning calorimetry, and enzyme assay. Lysozyme partition behavior was characterized in terms of extraction efficiency (%EE), and partition Lys was characterized by enzyme assay, differential scanning calorimetry, circular dichroism spectroscopy, and SDS-polyacrylamide gel electrophoresis. The structure, stability, and function of lysozyme were preserved or even improved (FIL-rich phase, glucose-rich phase, and [$N_{1112(\mathrm{OH})}$][H_2_PO_4_]-rich phase) during the extraction step. Results demonstrate that the FIL-based ABSs are more versatile and amenable to being tuned by the judiciously selection of the ABS phase-forming components, and permits the election of the enriched phase. Binding studies between lysozyme and ABS phase-forming components were evaluated by MST, disclosing a strong interaction between FILs ([C_2_C_1_Im][C_4_F_9_SO_3_] and [$N_{1112(\mathrm{OH})}$][C_4_F_9_SO_3_]) aggregates and the protein. Binding were not detected with mere fluoro-containing IL ([C_4_C_1_Im][CF_3_SO_3_]) and known globular protein stabilizers ([$N_{1112(\mathrm{OH})}$][H_2_PO_4_]). Further, two of the functionalized biocompatible FIL-based ABSs allowed the simultaneous purification of Lys and BSA (globular proteins) in a single ABS extraction step with high yield and purity (assessed by SDS-PAGE analysis). The two FIL-based ABSs comprise the [C_2_C_1_Im][C_4_F_9_SO_3_] FIL in combination with K_3_PO_4_ and sucrose (globular protein stabilizer), 30%wt [C_2_C_1_Im][C_4_F_9_SO_3_] + 2%wt K_3_PO_4_ and 30%wt [C_2_C_1_Im][C_4_F_9_SO_3_] + 25%wt sucrose, where the Lys-enriched phase is the FIL-rich phase (bottom phase) and the BSA enriched phase is the K_3_PO_4_-rich phase and sucrose-rich phase (top phase). Even with high-charge density salt K_3_PO_4_, where the salting-out effect of the salt favors the extraction of target products to the conventional IL-rich phase, the FIL-based ABSs developed in this work are more amenable to be tuned. Lys and BSA were purified through selective partition to opposite phases in a single FIL-based ABS extraction step. It was validated that the FIL-based ABS functionalization permits the election of the enriched phase. Regarding their biocompatibility, tailor-made properties and selectivity, FIL-based ABSs are proposed as an enhanced extraction step for proteins.

## Figures and Tables

**Table 1:** ABS phase properties of the selected biphasic system (biphasic points, BP), namely volume ratio, pH, and composition of both ionic-liquid-rich phase (bottom phase; FIL-rich phase, and mere fluoro-containing IL-rich phase) and non-ionic-liquid-rich phase (top phase; K_3_PO_4_-rich phase, sucrose-rich phase, glucose-rich phase, and [$N_{1112(\mathrm{OH})}$][H_2_PO_4_]-rich phase), at 25 °C.

			Ionic-Liquid-Rich Phase (Bottom Phase)				Non-Ionic-Liquid-Rich Phase (Top Phase)			
**BP#**	**ABS Composition (%wt)**	**Volume Ratio ^†^**	**pH**	**%wt H_2_O**	**%wt IL**	**%wt non-IL**	**pH**	**%wt H_2_O**	**%wt IL**	**%wt non-IL**
BP#1	30% [C_2_C_1_Im][CF_3_SO_3_] + 10% K_3_PO_4_	0.60	13.44	40.8785	56.5614	2.0652	13.18	73.4861	8.7068	16.8167
BP#2	30% [C_4_C_1_Im][CF_3_SO_3_] + 5% K_3_PO_4_	0.40	12.87	34.7684	64.4549	0.8176	13.25	78.6973	14.3401	6.7798
BP#3	30% [C_2_C_1_Im][C_4_F_9_SO_3_] + 2% K_3_PO_4_	0.94	12.78	43.6779	54.0350	0.3349	12.70	88.5482	4.2344	6.7525
BP#4	30% [C_4_C_1_Im][CF_3_SO_3_] + 25% sucrose	0.39	5.00	31.1747	57.6533	10.3701	5.25	47.1635	17.7208	32.0208
BP#5	30% [C_2_C_1_Im][C_4_F_9_SO_3_] + 25% sucrose	0.33	6.75	27.6055	66.8372	6.2338	6.75	53.5971	15.5558	31.8248
BP#6	30% [C_4_C_1_Im][CF_3_SO_3_] + 25% glucose	0.42	5.25	24.8533	72.0629	4.7980	5.25	53.2700	11.9669	32.7273
BP#7	30% [C_2_C_1_Im][C_4_F_9_SO_3_] + 25% glucose	0.43	6.50	20.5454	76.1935	4.1664	6.50	58.9178	7.7100	34.4053
BP#8	30% [C_2_C_1_Im][C_4_F_9_SO_3_] + 6% [$N_{1112(\mathrm{OH})}$][H_2_PO_4_]	0.89	3.37	41.3607	55.5876	1.5481	3.41	84.2108	4.0618	12.1251
BP#9	30% [C_2_C_1_Im][C_4_F_9_SO_3_] + 10% [$N_{1112(\mathrm{OH})}$][H_2_PO_4_]	0.59	3.65	17.7743	83.7801	0.6989	3.56	67.3516	5.1015	28.3455
BP#10	30% [C_2_C_1_Im][C_4_F_9_SO_3_] + 20% [$N_{1112(\mathrm{OH})}$][H_2_PO_4_]	0.42	3.99	30.1971	69.0974	1.0569	4.03	83.6562	2.3820	15.7034
BP#11	30% [C_4_C_1_Im][CF_3_SO_3_] + 20% [$N_{1112(\mathrm{OH})}$][H_2_PO_4_]	0.25	3.87	27.6891	66.5303	5.7394	3.78	54.8263	21.0133	23.5764
BP#12	30% [$N_{1112(\mathrm{OH})}$][C_4_F_9_SO_3_] + 30% [$N_{1112(\mathrm{OH})}$][H_2_PO_4_]	0.60	4.15	25.3343	63.9061	10.1689	4.16	45.8046	7.6946	43.9659

^†^ $\frac{\mathrm{volume}\mathrm{IL}-\mathrm{rp}}{\mathrm{volume}\text{non-IL}-\mathrm{rp}}$

**Table 2:** Dissociation constant $K_{d}$ for FITC-Lys with different ligands, FILs ([C_2_C_1_Im][C_4_F_9_SO_3_] and [$N_{1112(\mathrm{OH})}$][C_4_F_9_SO_3_]), mere fluoro-containing IL ([C_4_C_1_Im][CF_3_SO_3_]), and a known globular protein stabilizer ([$N_{1112(\mathrm{OH})}$][H_2_PO_4_]), determined by the analysis of the fitting of the MST dose-curve responses (Figure S34 of Supplementary Materials). The concentration range of the first capillary (maximum concentration) as well as the corresponding aggregates (or monomers) of the ligand, are outlined.

Ligand	Max. Conc. (mM) (First Capillary)	$K_{d}$ (mM)
[C_2_C_1_Im][C_4_F_9_SO_3_]	]0, 14.40[ (monomer)	no binding detected
	[14.4, 34.48[ (1st CAC)	1.27 ± 0.64
	[34.48, 76.54[ (2nd CAC)	0.87 ± 0.36
[$N_{1112(\mathrm{OH})}$][C_4_F_9_SO_3_]	]0, 16.02[ (monomer)	no binding detected
	[16.02, 35.17[ (1st CAC)	6.05 ± 1.67
	[35.17, 185.65[ (2nd CAC)	6.25 ± 1.47
[C_4_C_1_Im][CF_3_SO_3_]	]0, 100] (monomer)	no binding detected
[$N_{1112(\mathrm{OH})}$][H_2_PO_4_]	]0, 100] (monomer)	no binding detected

## Data Availability

Data will be made available upon request.

## Supplementary Materials

The following supporting information can be downloaded at https://www.mdpi.com/article/10.3390/ijms25115766/s1.

## References

[1] Albertsson P.A. (1986). Partition of Cell Particles and Macromolecules.

[2] Freire M.G., Cláudio A.F.M., Araújo J.M., Coutinho J.A., Marrucho I.M., Lopes J.N.C., Rebelo L.P.N. (2012). Aqueous biphasic systems: A boost brought about by using ionic liquids. Chem. Soc. Rev..

[3] Ferreira A.M., Esteves P.D.O., Boal-Palheiros I., Pereiro A.B., Rebelo L.P.N., Freire M.G. (2016). Enhanced tunability afforded by aqueous biphasic systems formed by fluorinated ionic liquids and carbohydrates. Green Chem..

[4] Schwaminger S.P., Zimmermann I., Berensmeier S. (2022). Current research approaches in downstream processing of pharmaceutically relevant proteins. Curr. Opin. Biotechnol..

[5] Nunes J.C.F., Almeida M., Faria J., Silva C., Neves M., Freire M., Tavares A. (2022). Overview on Protein Extraction and Purification Using Ionic-Liquid-Based Processes. J. Solut. Chem..

[6] Przybycien T.M., Pujar N.S., Steele L.M. (2004). Alternative bioseparation operations: Life beyond packed-bed chromatography. Curr. Opin. Biotechnol..

[7] Thömmes J., Etzel M. (2007). Alternatives to Chromatographic Separations. Biotechnol. Prog..

[8] Lu Y.M., Yang Y.Z., Zhao X.D., Xia C.B. (2010). Bovine serum albumin partitioning in polyethylene glycol (PEG)/potassium citrate aqueous two-phase systems. Food Bioprod. Process..

[9] Madhusudhan M.C., Raghavarao K., Nene S. (2008). Integrated process for extraction and purification of alcohol dehydrogenase from Baker’s yeast involving precipitation and aqueous two phase extraction. Biochem. Eng. J..

[10] Louwrier A. (1999). Model isolations of nucleic acids from prokaryotic and eukaryotic sources using an organic/aqueous biphasic system. Biotechnol. Tech..

[11] Zhi W., Deng Q. (2006). Purification of salvianolic acid B from the crude extract of Salvia miltiorrhiza with hydrophilic organic/salt-containing aqueous two-phase system by counter-current chromatography. J. Chromatogr. A.

[12] Jorge A.M., Coutinho J.A., Pereira J.F. (2023). Hydrodynamics of cholinium chloride-based aqueous biphasic systems (ABS): A key study for their industrial implementation. Sep. Purif. Technol..

[13] Pereira J.F.B., Rebelo L.P.N., Rogers R.D., Coutinho J.A.P., Freire M.G. (2013). Combining ionic liquids and polyethylene glycols to boost the hydrophobic–hydrophilic range of aqueous biphasic systems. Phys. Chem. Chem. Phys..

[14] Rosa P.A.J., Azevedo A.M., Ferreira I.F., de Vries J., Korporaal R., Verhoef H.J., Visser T.J., Aires-Barros M.R. (2007). Affinity partitioning of human antibodies in aqueous two-phase systems. J. Chromatogr. A.

[15] Azevedo A.M., Rosa P.A.J., Ferreira I.F., Aires-Barros M.R. (2009). Chromatography-free recovery of biopharmaceuticals through aqueous two-phase processing. Trends Biotechnol..

[16] Santos J.H.P.M., Flores-Santos J.C., Meneguetti G.P., Rangel-Yagui C.O., Coutinho J.A.P., Vitolo M., Ventura S.P.M., Pessoa A. (2018). In situ purification of periplasmatic L-asparaginase by aqueous two phase systems with ionic liquids (ILs) as adjuvants. J. Chem. Technol. Biotechnol..

[17] Gutowski K.E., Broker G.A., Willauer H.D., Huddleston J.G., Swatloski R.P., Holbrey J.D., Rogers R.D. (2003). Controlling the aqueous miscibility of ionic liquids: Aqueous biphasic systems of water-miscible ionic liquids and water-structuring salts for recycle, metathesis, and separations. J. Am. Chem. Soc..

[18] Shahriari S., Tomé L.C., Araújo J.M., Rebelo L.P.N., Coutinho J.A., Marrucho I.M., Freire M.G. (2013). Aqueous biphasic systems: A benign route using cholinium-based ionic liquids. RSC Adv..

[19] Carvalho S.F., Pereiro A.B., Araújo J.M.M. (2024). Simultaneous Purification of Human Interferon Alpha-2b and Serum Albumin Using Bioprivileged Fluorinated Ionic Liquid-Based Aqueous Biphasic Systems. Int. J. Mol. Sci..

[20] Bastos J.C., Carvalho S.F., Welton T., Lopes J.N.C., Rebelo L.P.N., Shimizu K., Araújo J.M.M., Pereiro A.B. (2018). Design of task-specific fluorinated ionic liquids: Nanosegregation versus hydrogen-bonding ability in aqueous solutions. Chem. Commun..

[21] Pereiro A.B., Araújo J.M., Teixeira F.S., Marrucho I.M., Piñeiro M.M., Rebelo L.P.N. (2015). Aggregation behavior and total miscibility of fluorinated ionic liquids in water. Langmuir.

[22] Vieira N.S.M., Bastos J.C., Rebelo L.P.N., Matias A., Araújo J.M.M., Pereiro A.B. (2019). Human cytotoxicity and octanol/water partition coefficients of fluorinated ionic liquids. Chemosphere.

[23] Vieira N.S.M., Stolte S., Araújo J.M.M., Rebelo L.P.N., Pereiro A.B., Markiewicz M. (2019). Acute Aquatic Toxicity and Biodegradability of Fluorinated Ionic Liquids. ACS Sustain. Chem. Eng..

[24] Alves M., Vieira N.S.M., Rebelo L.P.N., Araújo J.M.M., Pereiro A.B., Archer M. (2017). Fluorinated ionic liquids for protein drug delivery systems: Investigating their impact on the structure and function of lysozyme. Int. J. Pharm..

[25] Ferreira M.L., Vieira N.S.M., Araújo J.M.M., Pereiro A.B. (2021). Unveiling the Influence of Non-Toxic Fluorinated Ionic Liquids Aqueous Solutions in the Encapsulation and Stability of Lysozyme. Sustain. Chem..

[26] Alves M.M.S., Araújo J.M.M., Martins I.C., Pereiro A.B., Archer M. (2021). Insights into the interaction of Bovine Serum Albumin with Surface-Active Ionic Liquids in aqueous solution. J. Mol. Liq..

[27] Alves M.M., Leandro P., Mertens H.D., Pereiro A.B., Archer M. (2022). Impact of Fluorinated Ionic Liquids on Human Phenylalanine Hydroxylase—A Potential Drug Delivery System. Nanomaterials.

[28] Ferreira M.L., Vieira N.S., Oliveira A.L., Araújo J.M., Pereiro A.B. (2022). Disclosing the Potential of Fluorinated Ionic Liquids as Interferon-Alpha 2b Delivery Systems. Nanomaterials.

[29] Haria M., Benfield P. (1995). Inlerferon-a-2a A Review of its Pharmacological Properties and Therapeutic Use in the Management of Viral Hepatitis. Drugs.

[30] Kobayashi K. (2006). Summary of recombinant human serum albumin development. Biologicals.

[31] He X.M., Carter D.C. (1992). Atomic structure and chemistry of human serum albumin. Nature.

[32] Ascoli G.A., Domenici E., Bertucci C. (2006). Drug binding to human serum albumin: Abridged review of results obtained with high-performance liquid chromatography and circular dichroism. Chirality.

[33] Schröder C. (2017). Proteins in Ionic Liquids: Current Status of Experiments and Simulations. Top. Curr. Chem..

[34] Molodenskiy D., Shirshin E., Tikhonova T., Gruzinov A., Peters G., Spinozzi F. (2017). Thermally induced conformational changes and protein–protein interactions of bovine serum albumin in aqueous solution under different pH and ionic strengths as revealed by SAXS measurements. Phys. Chem. Chem. Phys..

[35] Pillai V.V.S., Benedetto A. (2018). Ionic liquids in protein amyloidogenesis: A brief screenshot of the state-of-the-art. Biophys. Rev..

[36] Ferreira A.M., Faustino V.F.M., Mondal D., Coutinho J.A.P., Freire M.G. (2016). Improving the extraction and purification of immunoglobulin G by the use of ionic liquids as adjuvants in aqueous biphasic systems. J. Biotechnol..

[37] Sindhu A., Kumar S., Venkatesu P. (2022). Contemporary Advancement of Cholinium-Based Ionic Liquids for Protein Stability and Long-Term Storage: Past, Present, and Future Outlook. ACS Sustain. Chem. Eng..

[38] Swaminathan R., Ravi V.K., Kumar S., Kumar M.V.S., Chandra N. (2011). Lysozyme: A model protein for amyloid research. Adv. Protein Chem. Struct. Biol..

[39] Ferraboschi P., Ciceri S., Grisenti P. (2021). Applications of lysozyme, an innate immune defense factor, as an alternative antibiotic. Antibiotics.

[40] Imoto T., Forstert L.S., Rupley J.A., Tanaka F. (1972). Fluorescence of Lysozyme: Emissions from Tryptophan Residues 62 and 108 and Energy Migration. Proc. Natl. Acad. Sci. USA.

[41] Neves C.M., Ventura S.P., Freire M.G., Marrucho I.M., Coutinho J.A. (2009). Evaluation of cation influence on the formation and extraction capability of ionic-liquid-based aqueous biphasic systems. J. Phys. Chem. B.

[42] Ventura S.P., Sousa S.G., Serafim L.S., Lima Á.S., Freire M.G., Coutinho J.A. (2011). Ionic liquid based aqueous biphasic systems with controlled pH: The ionic liquid cation effect. J. Chem. Eng. Data.

[43] Dai Y., Witkamp G.J., Verpoorte R., Choi Y.H. (2015). Tailoring properties of natural deep eutectic solvents with water to facilitate their applications. Food Chem..

[44] Merchuk J.C., Andrews B.A., Asenjo J.A. (1998). Aqueous two-phase systems for protein separation: Studies on phase inversion. J. Chromatogr. B Biomed. Sci. Appl..

[45] Bridges N.J., Gutowski K.E., Rogers R.D. (2007). Investigation of aqueous biphasic systems formed from solutions of chaotropic salts with kosmotropic salts (salt–salt ABS). Green Chem..

[46] Ventura S.P.M., Sousa S.G., Serafim L.S., Lima Á.S., Freire M.G., Coutinho J.A.P. (2012). Ionic-Liquid-Based Aqueous Biphasic Systems with Controlled pH: The Ionic Liquid Anion Effect. J. Chem. Eng. Data.

[47] Khan I., Kurnia K.A., Mutelet F., Pinho S.P., Coutinho J.A.P. (2014). Probing the Interactions between Ionic Liquids and Water: Experimental and Quantum Chemical Approach. J. Phys. Chem. B.

[48] Li Z., Liu X., Pei Y., Wang J., He M. (2012). Design of environmentally friendly ionic liquid aqueous two-phase systems for the efficient and high activity extraction of proteins. Green Chem..

[49] Pereira J.F.B., Kurnia K.A., Freire M.G., Coutinho J.A.P., Rogers R.D. (2015). Controlling the Formation of Ionic-Liquid-based Aqueous Biphasic Systems by Changing the Hydrogen-Bonding Ability of Polyethylene Glycol End Groups. ChemPhysChem.

[50] Carrero-Carralero C., Ruiz-Aceituno L., Ramos L., Moreno F.J., Sanz M.L. (2014). Influence of Chemical Structure on the Solubility of Low Molecular Weight Carbohydrates in Room Temperature Ionic Liquids. Ind. Eng. Chem. Res..

[51] Paduszyński K., Okuniewski M., Domańska U. (2013). “Sweet-in-Green” Systems Based on Sugars and Ionic Liquids: New Solubility Data and Thermodynamic Analysis. Ind. Eng. Chem. Res..

[52] Swatloski R.P., Spear S.K., Holbrey J.D., Rogers R.D. (2002). Dissolution of Cellose with Ionic Liquids. J. Am. Chem. Soc..

[53] Kumari M., Dohare N., Maurya N., Dohare R., Patel R. (2017). Effect of 1-methyl-3-octyleimmidazolium chloride on the stability and activity of lysozyme: A spectroscopic and molecular dynamics studies. J. Biomol. Struct. Dyn..

[54] Kumari M., Singh U.K., Beg I., Alanazi A.M., Khan A.A., Patel R. (2018). Effect of cations and anions of ionic liquids on the stability and activity of lysozyme: Concentration and temperature effect. J. Mol. Liq..

[55] Rodrigues J.V., Prosinecki V., Marrucho I., Rebelo L.P.N., Gomes C.M. (2011). Protein stability in an ionic liquid milieu: On the use of differential scanning fluorimetry. Phys. Chem. Chem. Phys..

[56] Satish L., Rana S., Arakha M., Rout L., Ekka B., Jha S., Dash P., Sahoo H. (2016). Impact of imidazolium-based ionic liquids on the structure and stability of lysozyme. Spectrosc. Lett..

[57] Vrikkis R.M., Fraser K.J., Fujita K., MacFarlane D.R., Elliott G.D. (2009). Biocompatible Ionic Liquids: A New Approach for Stabilizing Proteins in Liquid Formulation. J. Biomech. Eng..

[58] Pandit S., Kundu S., Abbas S., Aswal V.K., Kohlbrecher J. (2018). Structures and interactions among lysozyme proteins below the isoelectric point in presence of divalent ions. Chem. Phys. Lett..

[59] Rather M.A., Dar T.A., Singh L.R., Rather G.M., Bhat M.A. (2020). Structural-functional integrity of lysozyme in imidazolium based surface active ionic liquids. Int. J. Biol. Macromol..

[60] Mandal B., Mondal S., Pan A., Moulik S.P., Ghosh S. (2015). Physicochemical study of the interaction of lysozyme with surface active ionic liquid 1-butyl-3-methylimidazolium octylsulfate [BMIM] [OS] in aqueous and buffer media. Colloids Surfaces A Physicochem. Eng. Asp..

[61] Mandal B., Ghosh S., Moulik S.P. (2016). Detailed characterization of lysozyme (Lyz)–surfactant (SDDS) interaction and the structural transitions. New J. Chem..

[62] Bisht M., Kumar A., Venkatesu P. (2015). Analysis of the driving force that rule the stability of lysozyme in alkylammonium-based ionic liquids. Int. J. Biol. Macromol..

[63] Segawa S.I., Sugihara M. (1984). Characterization of the transition state of lysozyme unfolding. II. Effects of the intrachain crosslinking and the inhibitor binding on the transition state. Biopolymers.

[64] Greenfield N.J. (2007). Using circular dichroism spectra to estimate protein secondary structure. Nat. Protoc..

[65] Andrade M.A., Chactfn P., Merelo J.J., Mordn F. (1993). Evaluation of secondary structure of proteins from UV circular dichroism spectra using an unsupervised learning neural network. Protein Eng. Des. Sel..

[66] Corin K., Baaske P., Ravel D.B., Song J., Brown E., Wang X., Wienken C.J., Jerabek-Willemsen M., Duhr S., Luo Y. (2011). Designer Lipid-Like Peptides: A Class of Detergents for Studying Functional Olfactory Receptors Using Commercial Cell-Free Systems. PLoS ONE.

[67] Lakowicz J. (2006). Principles of Fluorescence Spectroscopy.

[68] Weaver K.D., Vrikkis R.M., Vorst M.P.V., Trullinger J., Vijayaraghavan R., Foureau D.M., McKillop I.H., MacFarlane D.R., Krueger J.K., Elliott G.D. (2012). Structure and function of proteins in hydrated choline dihydrogen phosphate ionic liquid. Phys. Chem. Chem. Phys..

[69] Kumar S., Kukutla P., Devunuri N., Venkatesu P. (2020). How does cholinium cation surpass tetraethylammonium cation in amino acid-based ionic liquids for thermal and structural stability of serum albumins?. Int. J. Biol. Macromol..

[70] Forciniti D. (2000). Studying the Influence of Salts on Partitioning of Proteins.

[71] Guan Y., Lilley T.H., Treffry T.E., Zhou C.L., Wilkinson P.B. (1996). Use of aqueous two-phase systems in the purification of human interferon-*α*1 from recombinant *Escherichia coli*. Enzym. Microb. Technol..

[72] Vieira N.S., Castro P.J., Marques D.F., Araújo J.M., Pereiro A.B. (2020). Tailor-made fluorinated ionic liquids for protein delivery. Nanomaterials.

[73] Lopes R.R., Tomé C.S., Russo R., Paterna R., Leandro J., Candeias N.R., Gonçalves L.M., Teixeira M., Sousa P.M., Guedes R.C. (2021). Modulation of human phenylalanine hydroxylase by 3-hydroxyquinolin-2(1h)-one derivatives. Biomolecules.

[74] Toro T.B., Nguyen T.P., Watt T.J. (2015). An improved 96-well turbidity assay for T4 lysozyme activity. Methods X.

[75] Johnston M.J.W., Nemr K., Hefford M.A. (2010). Influence of bovine serum albumin on the secondary structure of interferon alpha 2b as determined by far UV circular dichroism spectropolarimetry. Biologicals.

[76] Johnson C.M. (2013). Differential scanning calorimetry as a tool for protein folding and stability. Arch. Biochem. Biophys..

[77] Shugar D. (1952). The measurement of lysozyme activity and the ultra-violet inactivation of lysozyme. Biochim. Biophys. Acta.

[78] Ferreira M.L., Ferreira A.S.D., Araújo J.M.M., Cabrita E.J., Pereiro A.B. (2022). The impact of fluorinated ionic liquids aggregation in the interactions with proteins. Fluid Phase Equilibria.

[79] Li R., Wu Z., Wangb Y., Ding L., Wang Y. (2016). Role of pH-induced structural change in protein aggregation in foam fractionation of bovine serum albumin. Biotechnol. Rep..

[80] Hédoux A., Willart J.F., Paccou L., Guinet Y., Affouard F., Lerbret A., Descamps M. (2009). Thermostabilization Mechanism of Bovine Serum Albumin by Trehalose. J. Phys. Chem. B.

[81] Banipal T.S., Kaur A., Banipal P.K. (2017). Physicochemical aspects of the energetics of binding of sulphanilic acid with bovine serum albumin. Spectrochim. Acta Part A Mol. Biomol. Spectrosc..

[82] Russell B.A., Kubiak-Ossowska K., Mulheran P.A., Birch D.J.S., Chen Y. (2015). Locating the nucleation sites for protein encapsulated gold nanoclusters: A molecular dynamics and fluorescence study. Phys. Chem. Chem. Phys..

[83] Rezwan K., Studart A.R., Vörös J., Gauckler L.J. (2005). Change of *ζ* Potential of Biocompatible Colloidal Oxide Particles upon Adsorption of Bovine Serum Albumin and Lysozyme. J. Phys. Chem. B.

[84] Andrews B.A., Schmidt A.S., Asenjo J.A. (2005). Correlation for the partition behavior of proteins in aqueous two-phase systems: Effect of surface hydrophobicity and charge. Biotechnol. Bioeng..

[85] Moon Y.U., Curtis R.A., Anderson C.O., Blanch H.W., Prausnitz J.M. (2000). Protein—Protein Interactions in Aqueous Ammonium Sulfate Solutions. Lysozyme and Bovine Serum Albumin (BSA). J. Solut. Chem..

[86] Townsend R.J., Hill M., Harris N.R., White N.M. (2004). Modelling of particle paths passing through an ultrasonic standing wave. Ultrasonics.

[87] Santos M.B., de Carvalho C.W.P., Garcia-Rojas E.E. (2018). Heteroprotein complex formation of bovine serum albumin and lysozyme: Structure and thermal stability. Food Hydrocoll..

[88] Sarmah R.J., Kundu S. (2022). Structure and morphology of bovine serum albumin–lysozyme (BSA–Lys) complex films at air–water interface. Food Hydrocoll..

[89] Mao Y., Yu L., Yang R., Qu L.B., Harrington P.D.B. (2015). A novel method for the study of molecular interaction by using microscale thermophoresis. Talanta.

[90] Jerabek-Willemsen M., André T., Wanner R., Roth H.M., Duhr S., Baaske P., Breitsprecher D. (2014). MicroScale Thermophoresis: Interaction analysis and beyond. J. Mol. Struct..

[91] Wienken C.J., Baaske P., Rothbauer U., Braun D., Duhr S. (2010). Protein-binding assays in biological liquids using microscale thermophoresis. Nat. Commun..

[92] Khavrutskii L.A., Yeh J.A., Timofeeva O.A., Tarasov S.G.A., Pritt S.A., Stefanisko K.A., Tarasova N.A. (2013). Protein Purification-free Method of Binding Affinity Determination by Microscale Thermophoresis. JoVE.

[93] Jerabek-Willemsen M., Wienken C.J., Braun D., Baaske P., Duhr S. (2011). Molecular interaction studies using microscale thermophoresis. Assay Drug Dev. Technol..

[94] Zahradník J., Kolářová L., Pařízková H., Kolenko P., Schneider B. (2018). Interferons type II and their receptors R1 and R2 in fish species: Evolution, structure, and function. Fish Shellfish Immunol..

[95] Tosstorff A., Svilenov H., Peters G.H.J., Harris P., Winter G. (2019). Structure-based discovery of a new protein-aggregation breaking excipient. Eur. J. Pharm. Biopharm..

[96] Corrêa D., Henrique C., Ramos I., Corrêa D.H.A., Ramos C.H.I. (2009). The use of circular dichroism spectroscopy to study protein folding, form and function Structure and function of molecular chaperones View project Protein stability, folding and misfolding View project The use of circular dichroism spectroscopy to study protein folding, form and function. Afr. J. Biochem. Res..

[97] Sundaram V., Ramanan R.N., Selvaraj M., Vijayaraghavan R., MacFarlane D.R., Ooi C.W. (2022). Enhanced structural stability of insulin aspart in cholinium aminoate ionic liquids. Int. J. Biol. Macromol..

[98] MacKenzie R., Honig P., Sewards J., Goodwin R., Hellio M.P. (2020). COVID-19 must catalyse changes to clinical development. Nat. Rev. Drug Discov..

[99] Whitmore L., Wallace B.A. (2008). Protein secondary structure analyses from circular dichroism spectroscopy: Methods and reference databases. Biopolymers.

