# Supplementary material for: Towards Enhanced Tunability of Aqueous Biphasic Systems: Furthering the Grasp of Fluorinated Ionic Liquids in the Purification of Proteins
Source: Int J Mol Sci. 2024 May 25;25(11):5766. doi: 10.3390/ijms25115766 (PMC11172314; doi:10.3390/ijms25115766)
Supplement: Supplementary file 1 [file ijms-25-05766-s001.zip › ijms-2985327-supplementary.pdf]

# Supplementary Materials: Towards Enhanced Tunability of Aqueous Biphasic Systems: Furthering the Grasp of Fluorinated Ionic Liquids in the Purification of Proteins

**Sara F. Carvalho [1], Margarida H. Custódio [1], Ana B. Pereiro [1] and João M.M. Araújo [1,*]**

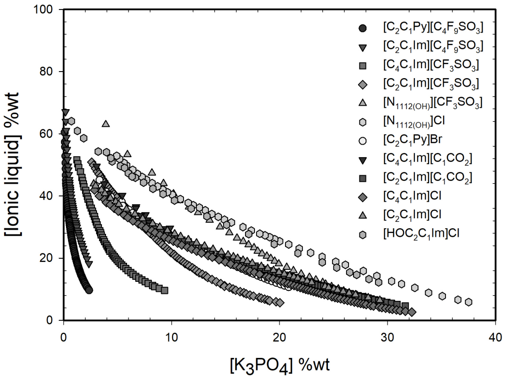


**Figure S1.** Ternary phase diagrams in weight mass % for all the studied conventional ILs ($[HOC_2C_1Im]Cl$, $[C_2C_1Im]Cl$, $[C_4C_1Im]Cl$, $[C_2C_1Im][C_1CO_2]$, $[C_4C_1Im][C_1CO_2]$, $[C_2C_1Py]Br$ and $[N_{1112(OH)}]Cl$), mere fluoro-containing ILs ($[C_2C_1Im][CF_3SO_3]$, $[C_4C_1Im][CF_3SO_3]$ and $[N_{1112(OH)}][CF_3SO_3]$) and fluorinated ILs ($[C_2C_1Im][C_4F_9SO_3]$ and $[C_2C_1Py][C_4F_9SO_3]$) combined with $K_3PO_4$ at 25 °C and atmospheric pressure.

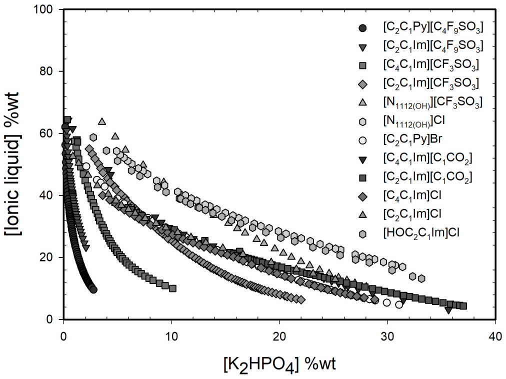


**Figure S2.** Ternary phase diagrams in weight mass % for all the studied conventional ILs ($[HOC_2C_1Im]Cl$, $[C_2C_1Im]Cl$, $[C_4C_1Im]Cl$, $[C_2C_1Im][C_1CO_2]$, $[C_4C_1Im][C_1CO_2]$, $[C_2C_1Py]Br$ and $[N_{1112(OH)}]Cl$), mere fluoro-containing ILs ($[C_2C_1Im][CF_3SO_3]$, $[C_4C_1Im][CF_3SO_3]$ and $[N_{1112(OH)}][CF_3SO_3]$) and fluorinated ILs ($[C_2C_1Im][C_4F_9SO_3]$ and $[C_2C_1Py][C_4F_9SO_3]$) combined with inorganic salt at 25 °C and atmospheric pressure.

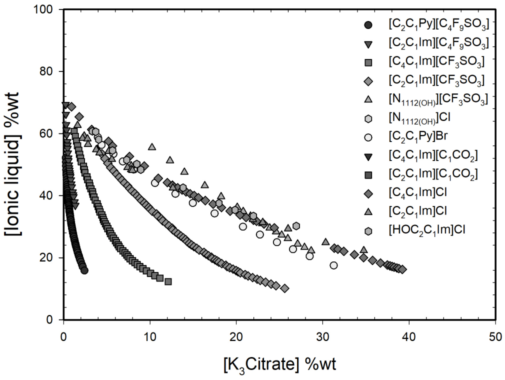


**Figure S3.** Ternary phase diagrams in weight mass % for all the studied conventional ILs ([$HOC_2C_1Im$]Cl, [$C_2C_1Im$]Cl, [$C_4C_1Im$]Cl, [$C_2C_1Im$][$C_1CO_2$], [$C_4C_1Im$][$C_1CO_2$], [$C_2C_1Py$]Br and [$N_{1112(OH)}$]Cl), mere fluoro-containing ILs ([$C_2C_1Im$][$CF_3SO_3$], [$C_4C_1Im$][$CF_3SO_3$] and [$N_{1112(OH)}$][$CF_3SO_3$]) and fluorinated ILs ([$C_2C_1Im$][$C_4F_9SO_3$] and [$C_2C_1Py$][$C_4F_9SO_3$]) combined with salt at 25 °C and atmospheric pressure.

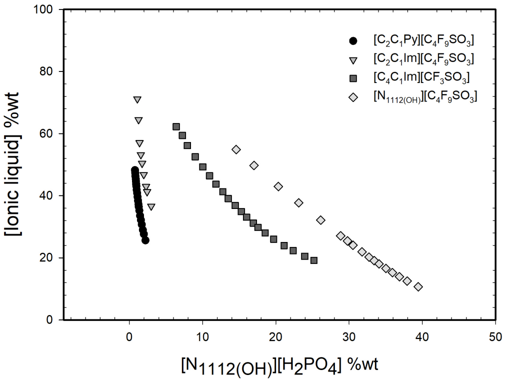


**Figure S4.** Ternary phase diagrams in weight mass % for all studied mere fluoro-containing ILs ([$C_2C_1Im$][$CF_3SO_3$]and [$C_4C_1Im$][$CF_3SO_3$]) and fluorinated ILs ( [$C_2C_1Py$][$C_4F_9SO_3$], [$C_2C_1Im$][$C_4F_9SO_3$] and [$N_{1112(OH)}$][$C_4F_9SO_3$]) combined with [$N_{1112(OH)}$][$H_2PO_4$] at 25 °C and atmospheric pressure.

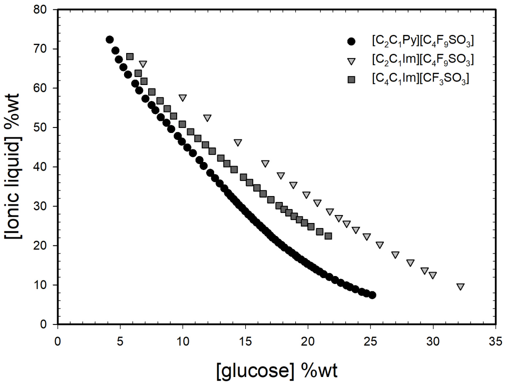


**Figure S5.** Ternary phase diagrams in weight mass % for the studied [$C_2C_1Py$][$C_4F_9SO_3$], [$C_2C_1Im$][$C_4F_9SO_3$] and [$C_4C_1Im$][$CF_3SO_3$] combined with sucrose at 25 °C and atmospheric pressure.

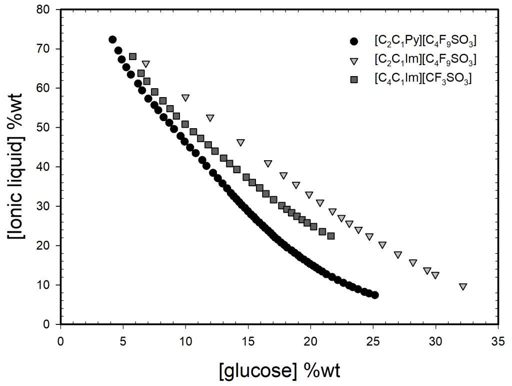


**Figure S6.** Ternary phase diagrams in weight mass % for the studied $[C_2C_1Py][C_4F_9SO_3]$, $[C_2C_1Im][C_4F_9SO_3]$ and $[C_4C_1Im][CF_3SO_3]$ combined with glucose at 25 °C and atmospheric pressure.

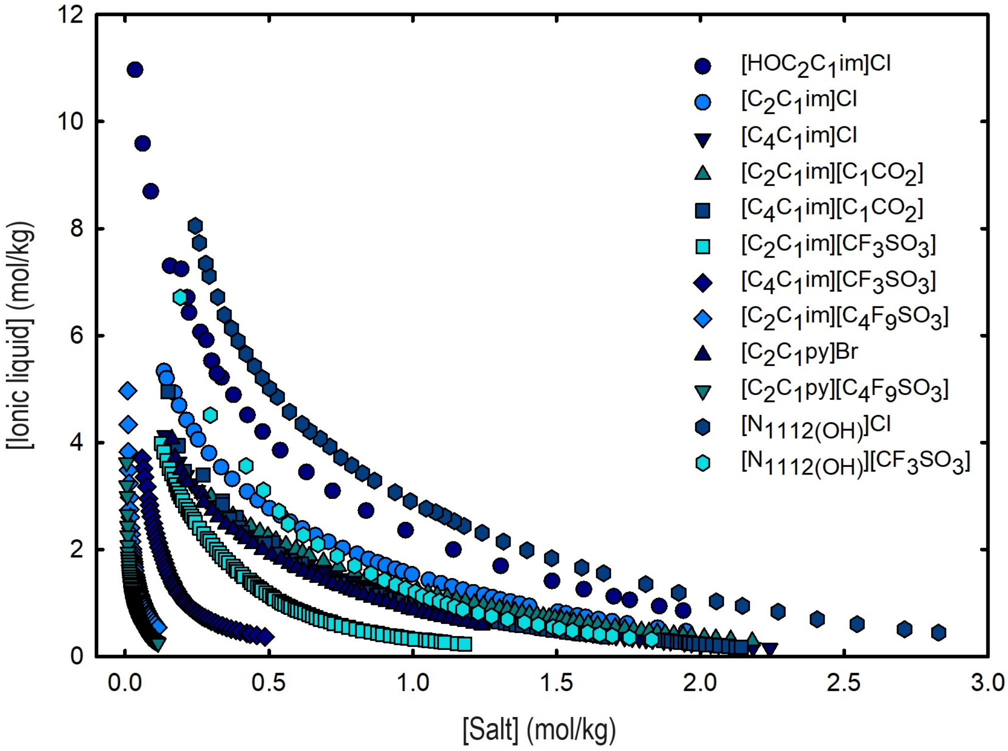


**Figure S7.** Ternary phase diagrams in molality for all the studied conventional ILs ($[HOC_2C_1Im]Cl$, $[C_2C_1Im]Cl$, $[C_4C_1Im]Cl$, $[C_2C_1Im][C_1CO_2]$, $[C_4C_1Im][C_1CO_2]$, $[C_2C_1Py]Br$ and $[N_{1112(OH)}]Cl$), mere fluoro-containing ILs ($[C_2C_1Im][CF_3SO_3]$, $[C_4C_1Im][CF_3SO_3]$ and $[N_{1112(OH)}][CF_3SO_3]$) and fluorinated ILs ($[C_2C_1Im][C_4F_9SO_3]$ and $[C_2C_1Py][C_4F_9SO_3]$) combined with $K_3PO_4$ at 25 °C and atmospheric pressure.

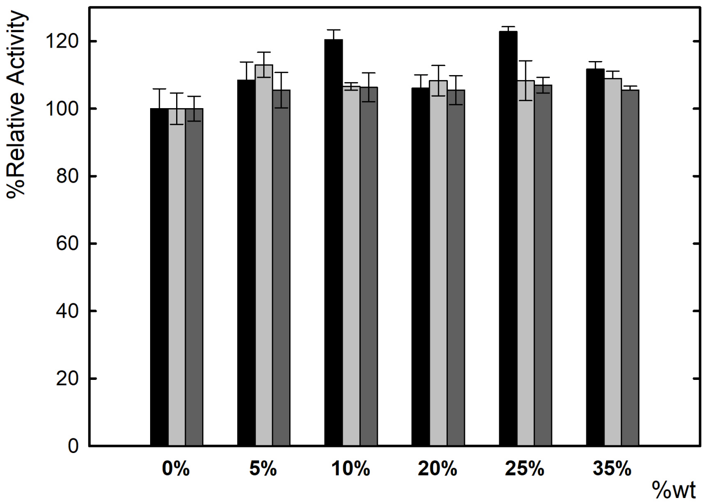


**Figure S8.** Relative Enzymatic Activity of Lysozyme in (black) 0.2, (light gray) 0.5 and (dark gray) 1.0 mg/mL at increasing concentrations of $[C_4C_1Im][CF_3SO_3]$ in water.

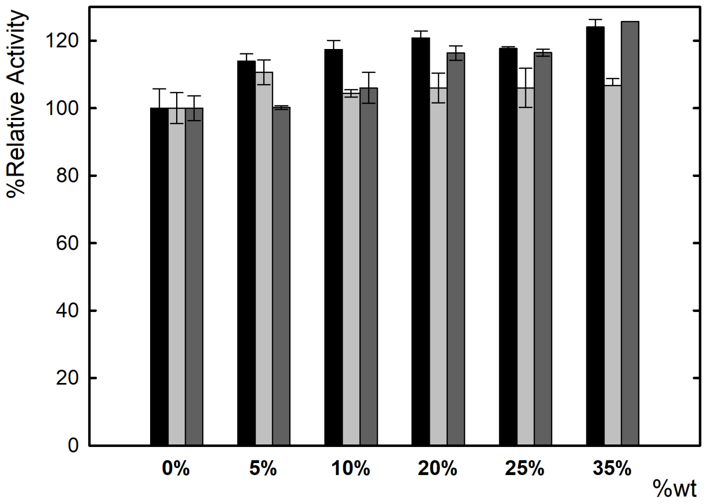


**Figure S9.** Relative Enzymatic Activity of Lysozyme in (black) 0.2, (light gray) 0.5 and (dark gray) 1.0 mg/mL at increasing concentrations of $[C_2C_1Im][C_4F_9SO_3]$ in water.

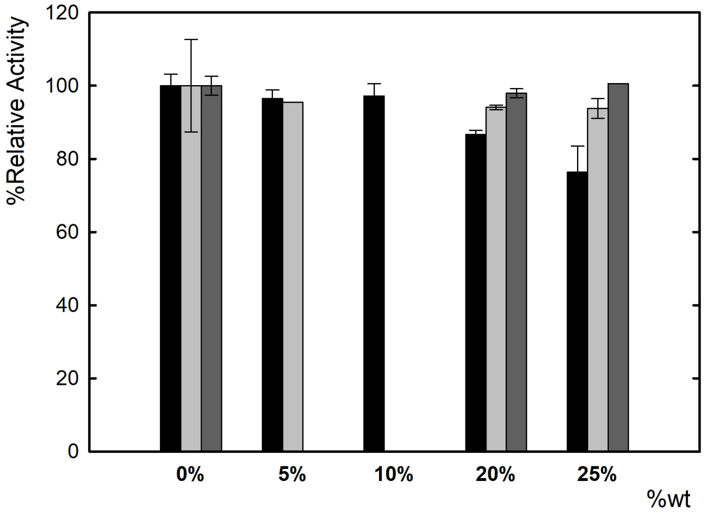


**Figure S10.** Relative Enzymatic Activity of Lysozyme in (black) 0.2, (light gray) 0.5 and (dark gray) 1.0 mg/mL at increasing concentrations of $[N_{1112(OH)}][C_4F_9SO_3]$ in water.

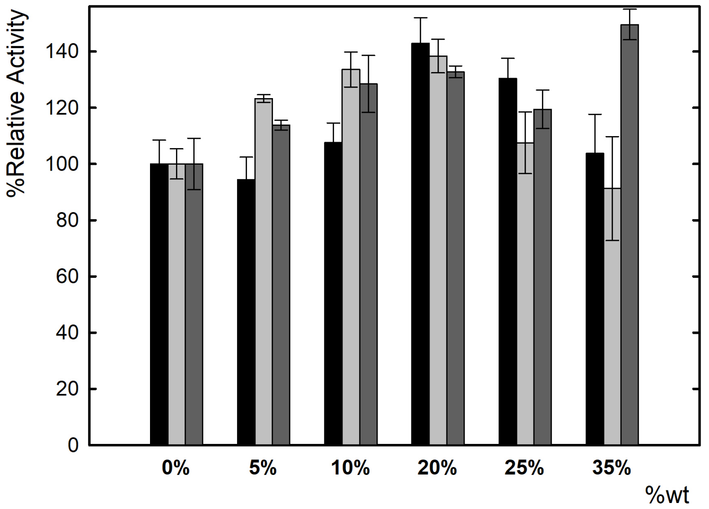


**Figure S11.** Relative Enzymatic Activity of Lysozyme in (black) 0.2, (light gray) 0.5 and (dark gray) 1.0 mg/mL at increasing concentrations of $K_3PO_4$ in water.

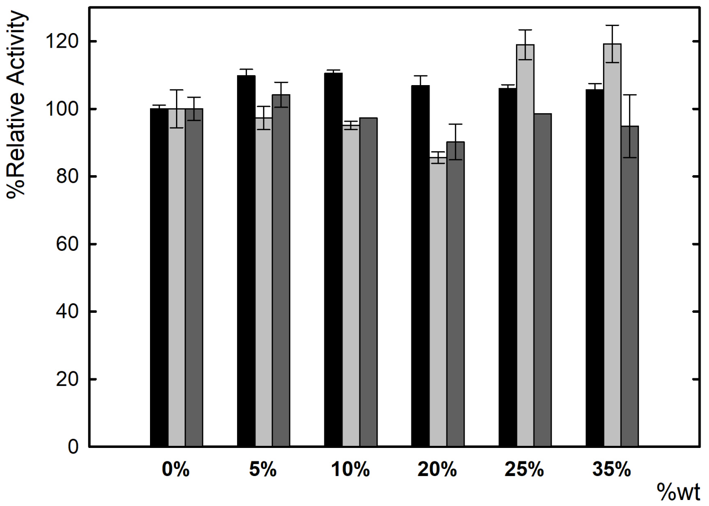


**Figure S12.** Relative Enzymatic Activity of Lysozyme in (black) 0.2, (light gray) 0.5 and (dark gray) 1.0 mg/mL at increasing concentrations of sucrose in water.

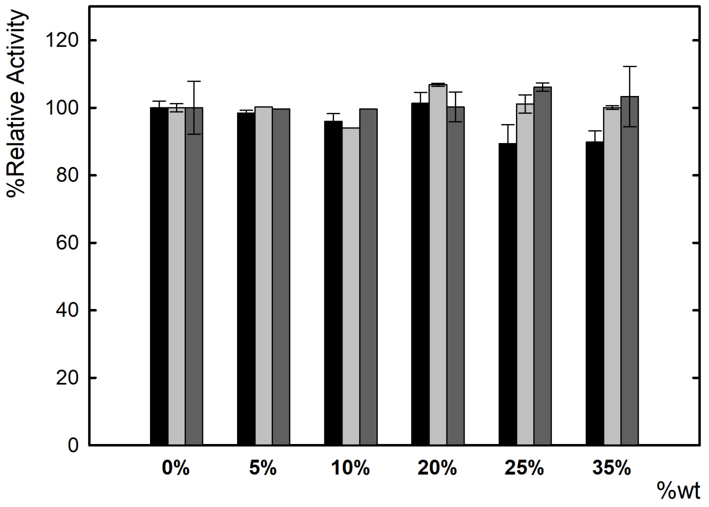


**Figure S13.** Relative Enzymatic Activity of Lysozyme in (black) 0.2, (light gray) 0.5 and (dark gray) 1.0 mg/mL at increasing concentrations of $[N_{1112(OH)}][H_2PO_4]$ in water.

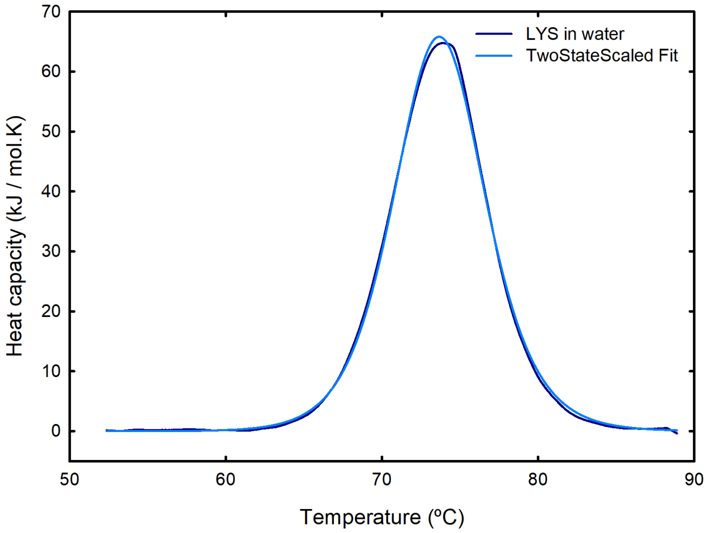


**Figure S14.** DSC curve and Two State Scaled Model fit of 1 mg/mL lysozyme in water. The scan rate was 1.0 °C/min with Heat capacity (Cp) as a function of Temperature, exo-up. The obtained Lys $T_m$ is 74.56±0.835 °C (Tables 2 and 5).

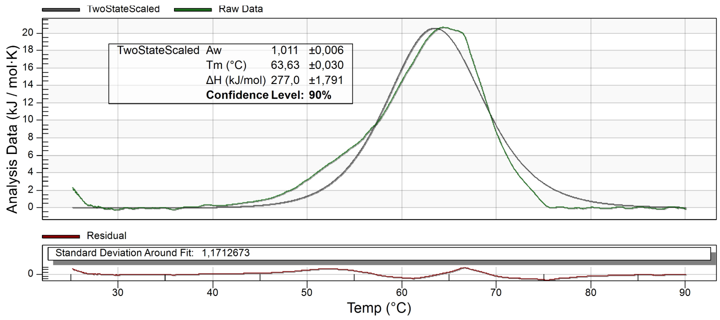


**Figure S15.** DSC curve and Two State Scaled Model fit of 1 mg/mL lysozyme in aqueous solution 25 mM $[C_2C_1Im][C_4F_9SO_3]$. The scan rate was 1.0 °C/min with Heat capacity (Cp) as a function of Temperature, exo-up. The obtained Lys $T_m$ is 63.63 °C (Table 2).

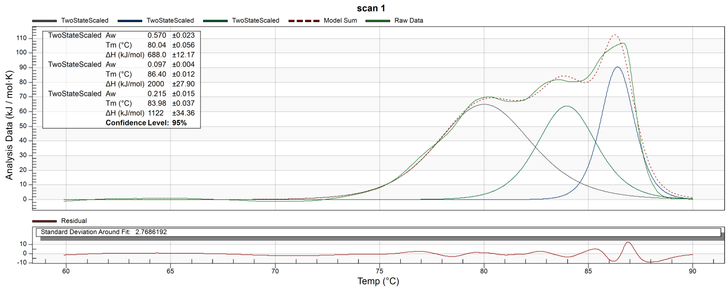


**Figure S16.** 1 mg/mL lysozyme in aqueous solution 25 mM $[N_{1112(OH)}][C_4F_9SO_3]$ DSC curve and Two State Scaled Model fitted with three peaks. The scan rate was 1.0 °C/min with Heat capacity (Cp) as a function of Temperature, exo-up. The obtained Lys $T_m$ is 80.04,83.98 and 86.40 °C.

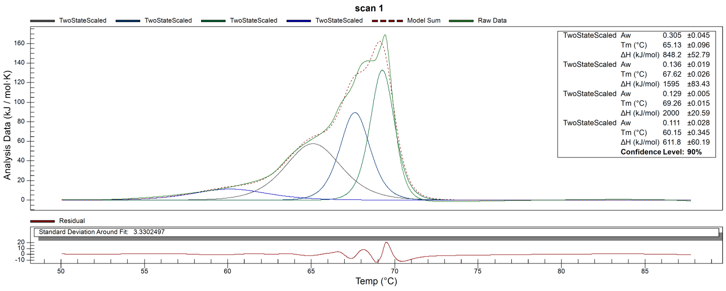


**Figure S17.** 1 mg/mL lysozyme in aqueous solution 200 mM $[N_{1112(OH)}][C_4F_9SO_3]$ DSC curve and Two State Scaled Model fitted with four peaks. The scan rate was 1.0 °C/min with Heat capacity (Cp) as a function of Temperature, exo-up. The obtained Lys $T_m$ is 60.15, 65.13, 67.62 and 69.26 °C.

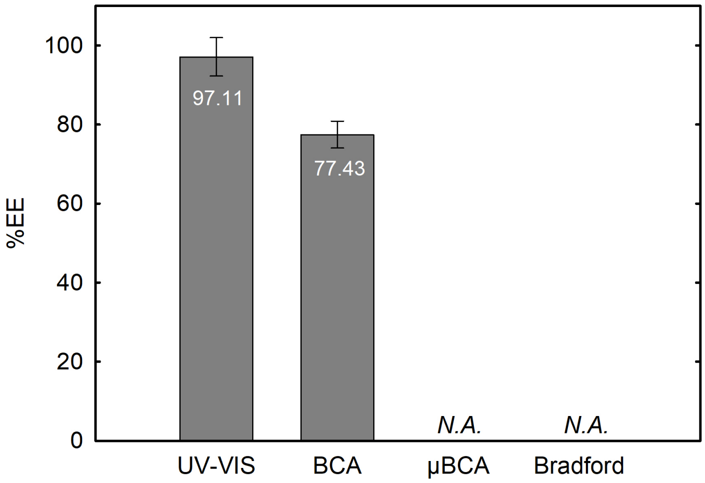


**Figure S18.** Extraction Efficiencies of Lysozyme in BP1 with 30%wt $[C_2C_1Im][CF_3SO_3]$ + 10%wt $K_3PO_4$ by respective detection method:UV-Vis; BCA; $\mu$ BCA; Bradford.

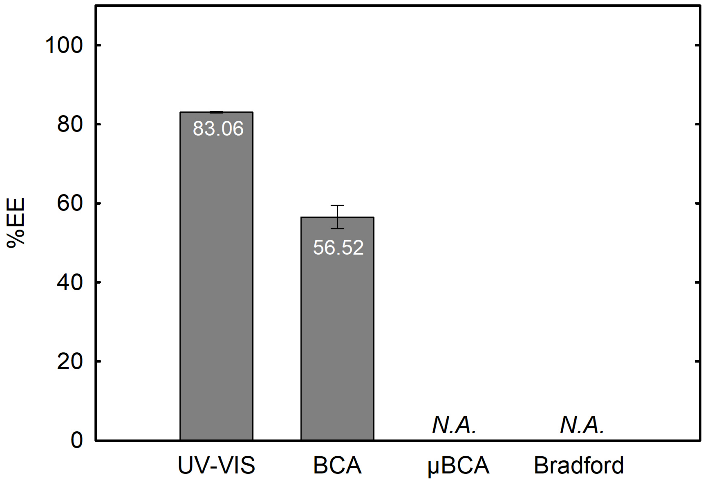


**Figure S19.** Extraction Efficiencies of Lysozyme in BP2 with 30%wt $[C_4C_1Im][CF_3SO_3]$ + 5%wt $K_3PO_4$ by respective detection method:UV-Vis; BCA; $\mu$ BCA; Bradford.

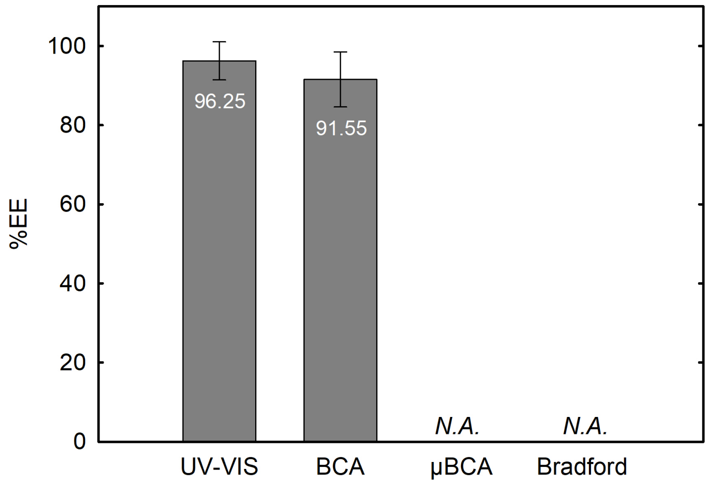


**Figure S20.** Extraction Efficiencies of Lysozyme in BP3 with 30%wt $[C_2C_1Im][C_4F_9SO_3]$ + 2%wt $K_3PO_4$ by respective detection method:UV-Vis; BCA; $\mu$ BCA; Bradford.

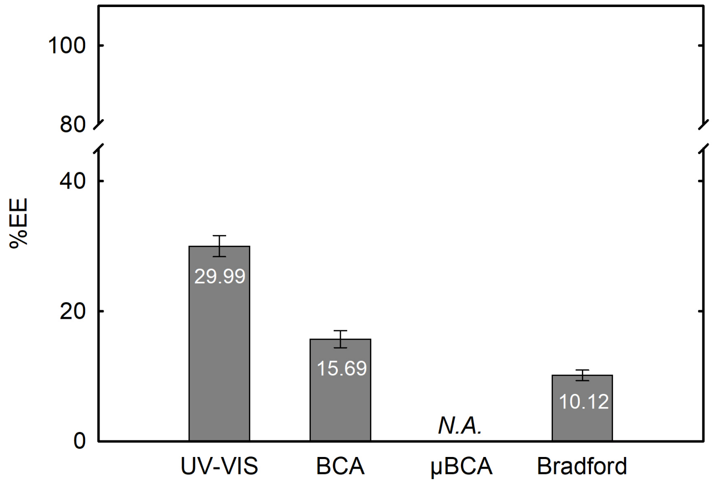


**Figure S21.** Extraction Efficiencies of Lysozyme in BP4 with 30%wt $[C_4C_1Im][CF_3SO_3]$ + 25%wt sucrose by respective detection method:UV-Vis; BCA; $\mu$ BCA; Bradford.

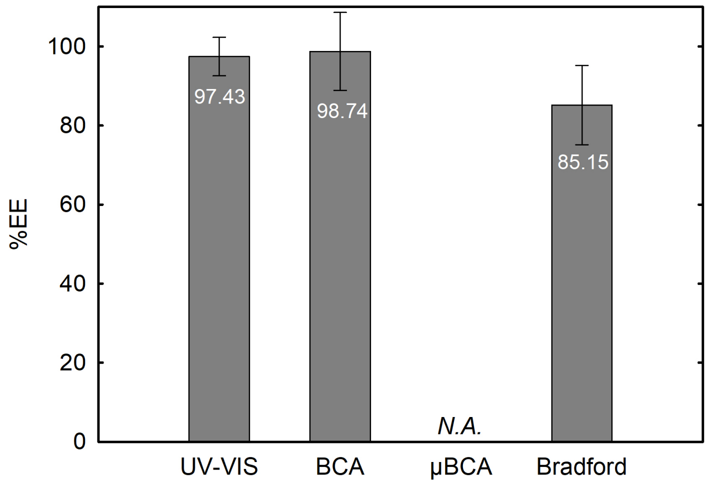


**Figure S22.** Extraction Efficiencies of Lysozyme BP5 with 30%wt [$C_2C_1$Im][$C_4F_9SO_3$] + 25%wt sucrose by respective detection method:UV-Vis; BCA; $\mu$ BCA; Bradford.

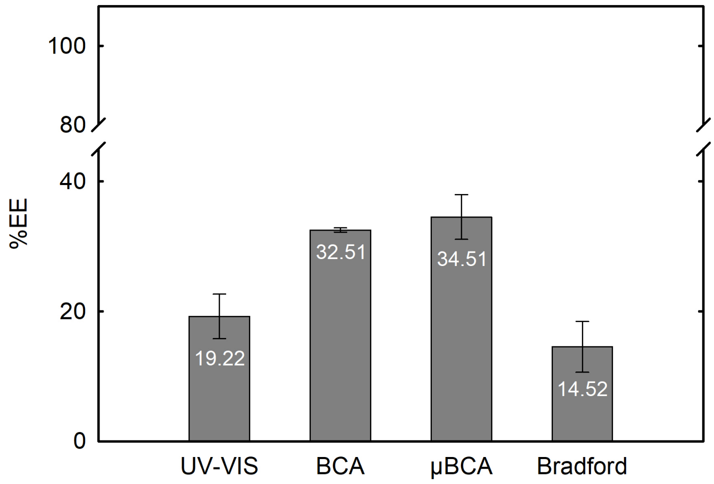


**Figure S23.** Extraction Efficiencies of Lysozyme BP6 with 30%wt [$C_4C_1$Im][$CF_3SO_3$] + 25%wt glucose by respective detection method:UV-Vis; BCA; $\mu$ BCA; Bradford.

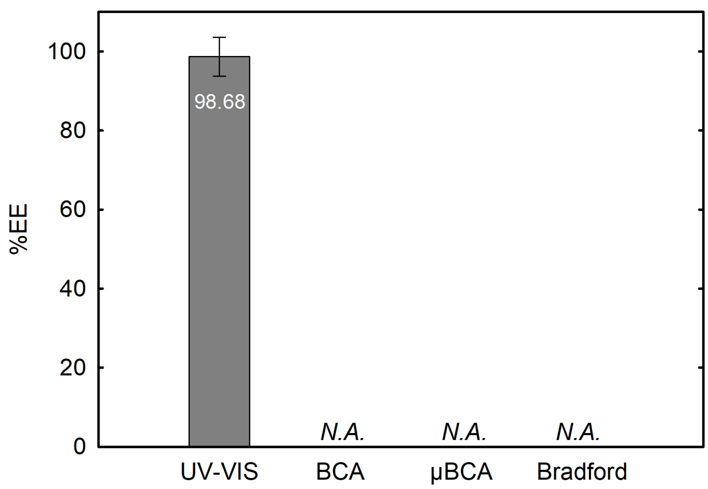


**Figure S24.** Extraction Efficiencies of Lysozyme BP7 with 30%wt [$C_2C_1$Im][$C_4F_9SO_3$] + 25%wt glucose by respective detection method:UV-Vis; BCA; $\mu$ BCA; Bradford.

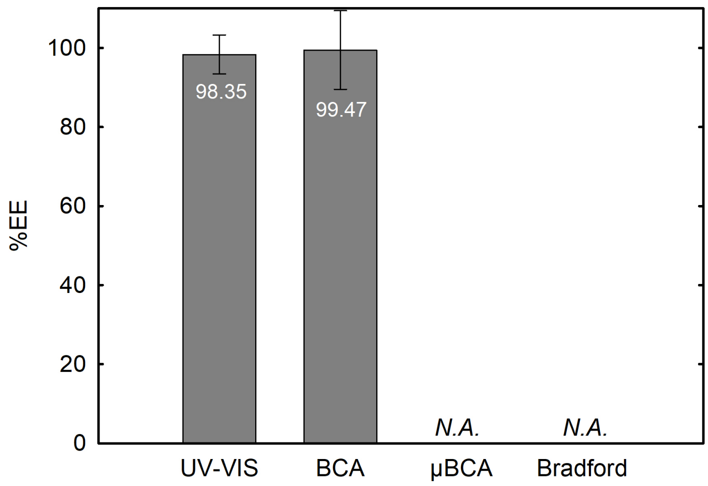


**Figure S25.** Extraction Efficiencies of Lysozyme in BP8 with 30%wt [$C_2C_1$Im][$C_4F_9SO_3$] + 6%wt [$N_{1112(OH)}$][$H_2PO_4$] by respective detection method:UV-Vis; BCA; $\mu$ BCA; Bradford.

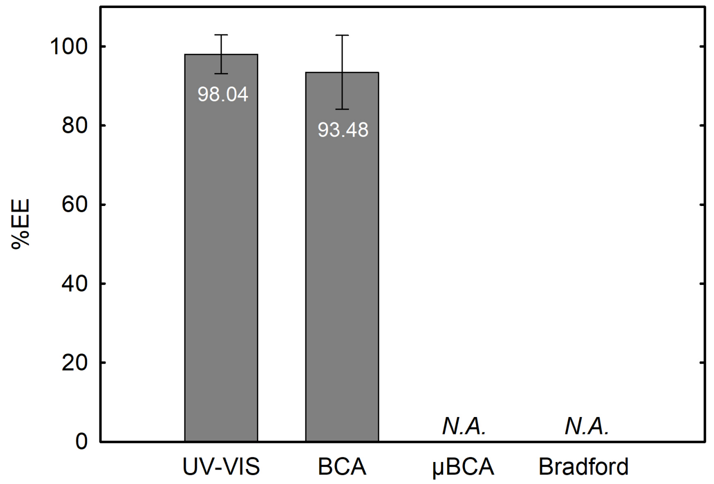


**Figure S26.** Extraction Efficiencies of Lysozyme in BP9 with 30%wt $[C_2C_1Im][C_4F_9SO_3]$ + 10%wt $[N_{1112(OH)}][H_2PO_4]$ by respective detection method:UV-Vis; BCA; $\mu$ BCA; Bradford.

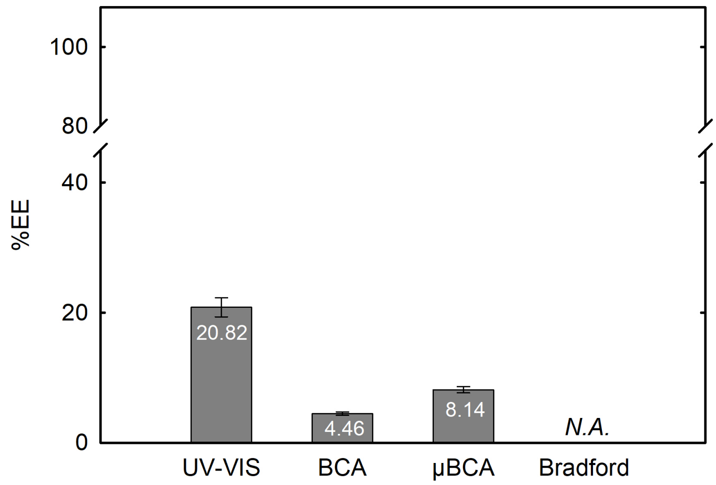


**Figure S27.** Extraction Efficiencies of Lysozyme in BP10 with 30%wt $[C_2C_1Im][C_4F_9SO_3]$ + 20%wt $[N_{1112(OH)}][H_2PO_4]$ by respective detection method:UV-Vis; BCA; $\mu$ BCA; Bradford.

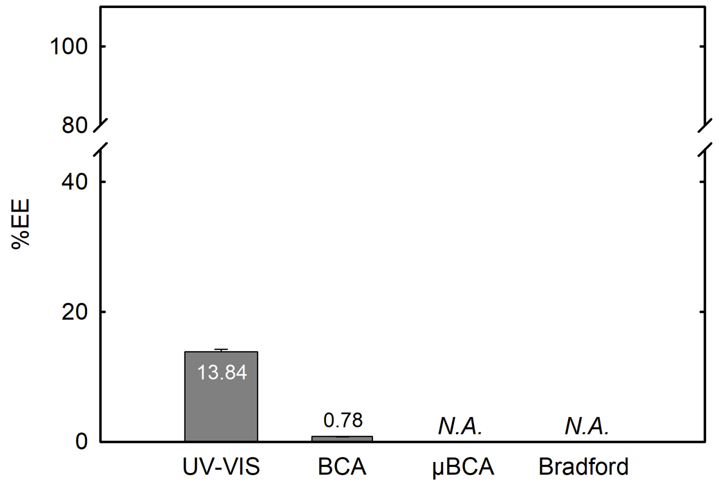


**Figure S28.** Extraction Efficiencies of Lysozyme in BP11 system 30%wt [$C_4C_1$Im][$CF_3SO_3$] + 20%wt [$N_{1112(OH)}$][$H_2PO_4$] by respective detection method:UV-Vis; BCA; $\mu$ BCA; Bradford.

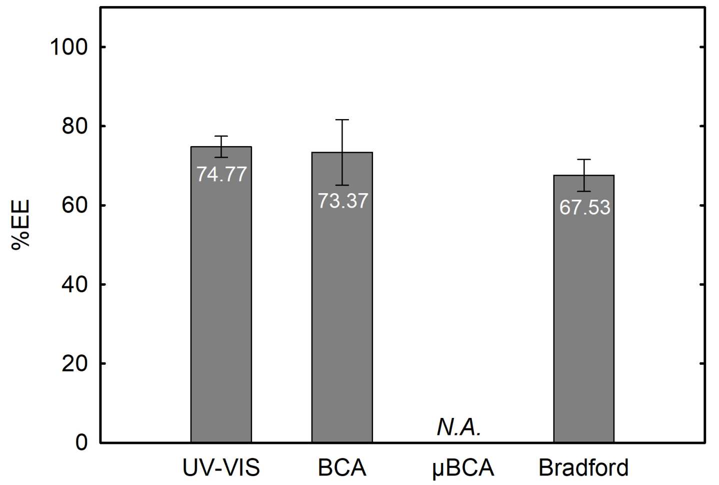


**Figure S29.** Extraction Efficiencies of Lysozyme in BP12 with 30%wt [$N_{1112(OH)}$][$C_4F_9SO_3$] + 30%wt [$N_{1112(OH)}$][$H_2PO_4$] by respective detection method:UV-Vis; BCA; $\mu$ BCA; Bradford.

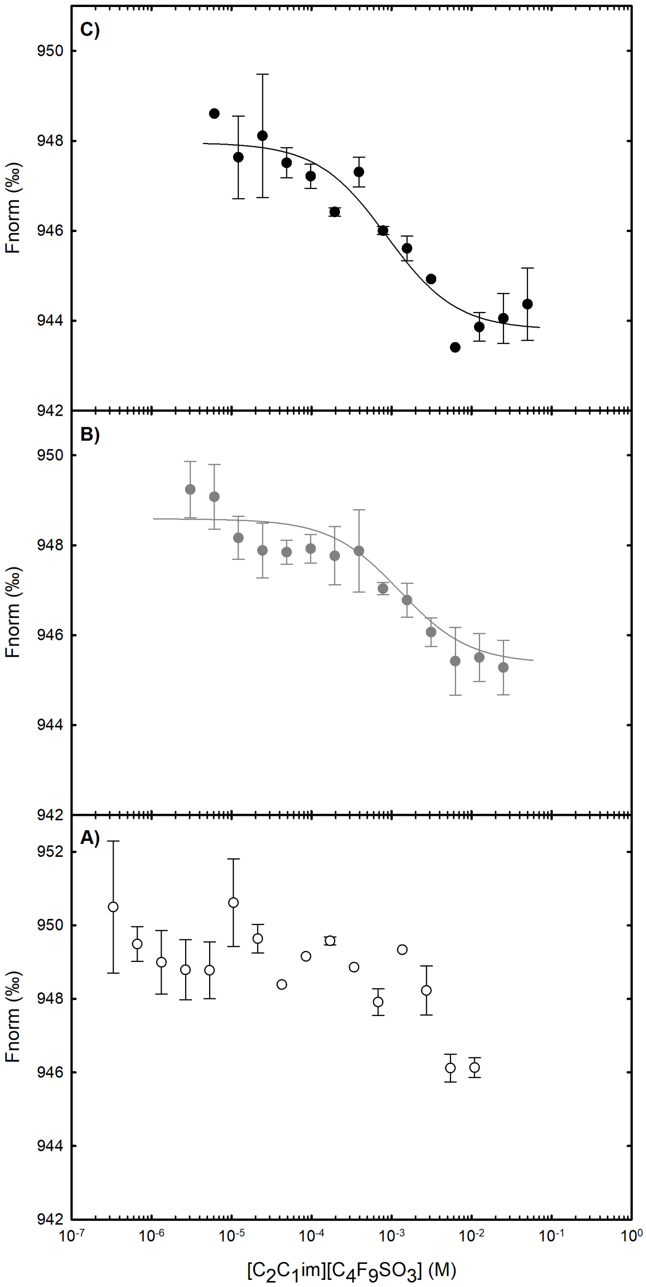


**Figure S30.** MST Binding curve analysis for interaction study between FITC-labeled lys and $[C_2C_1Im][C_4F_9SO_3]$: Normalized fluorescence (Fnorm) in ‰ is plotted over log of FIL concentration in mM. Starting concentrations A) above $2^{nd}$ CAC B) above $1^{st}$ CAC and C) below $1^{st}$ CAC. Error bars represent standard error of at least n=3 measurements.

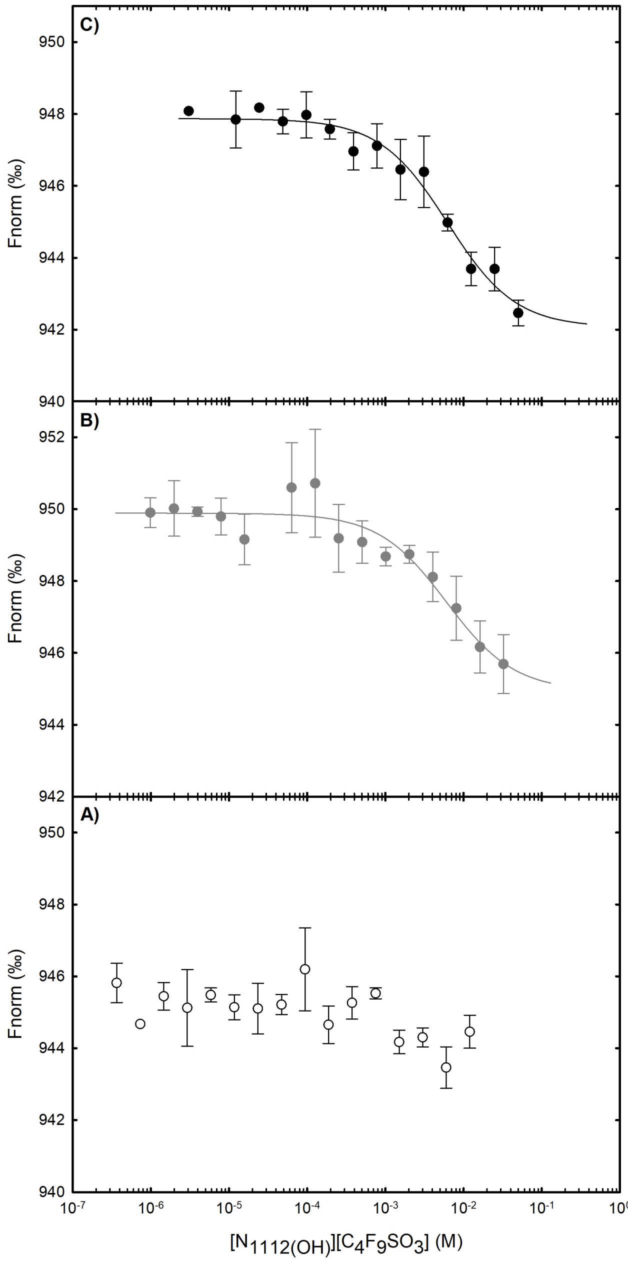


**Figure S31.** MST Binding curve analysis for interaction study between FITC-labeled lys and [$N_{1112(OH)}$][$C_4F_9SO_3$]: Normalized fluorescence (Fnorm) in ‰ is plotted over log of FIL concentration in mM. Starting concentrations A) above $2^{nd}$ CAC B) above $1^{st}$ CAC and C) below $1^{st}$ CAC. Error bars represent standard error of at least n=3 measurements.

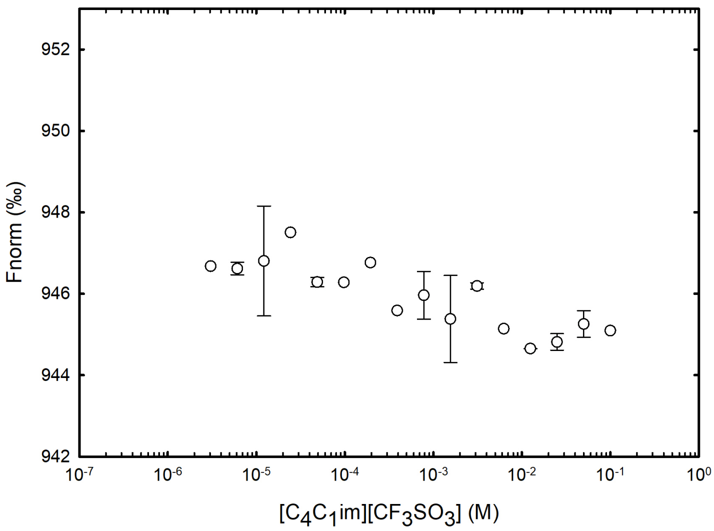


**Figure S32.** MST Binding curve analysis for interaction study between FITC-labeled lys and $[C_4C_1Im][CF_3SO_3]$: Normalized fluorescence (Fnorm) in ‰ is plotted over log of IL concentration in mM. Error bars represent standard error of at least n=3 measurements.

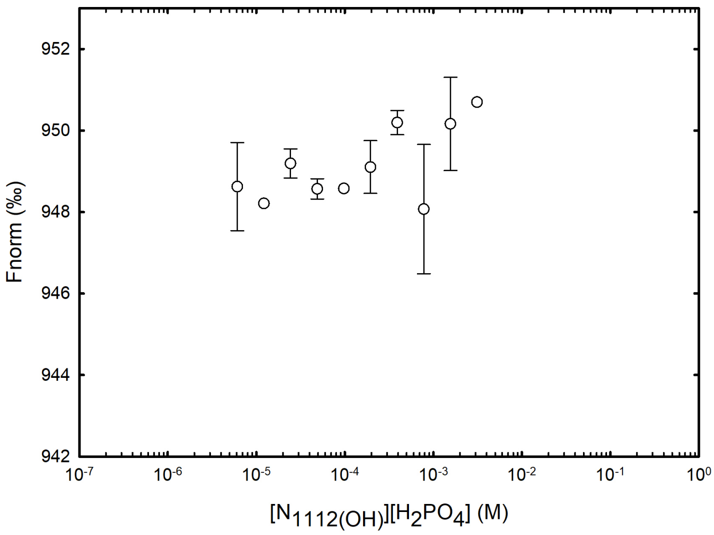


**Figure S33.** MST Binding curve analysis for interaction study between FITC-labeled lys and $[N_{1112(OH)}][H_2PO_4]$: Normalized fluorescence (Fnorm) in ‰ is plotted over log of salt concentration in mM. Error bars represent standard error of at least n=3 measurements.

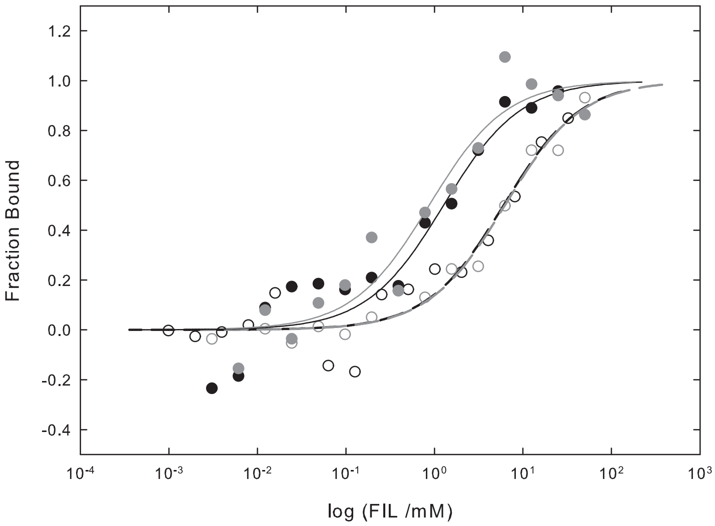


**Figure S34.** Fraction Bound data and fitting over log FIL concentration. Systems $[C_2C_1Im][C_4F_9SO_3]$ and  above the $1^{st}$ and $2^{nd}$ CAC's

**Table S1.** Acronym, designation, purity and respective chemical structure of all ILs and FILs under study.

| **Acronym** | **IL/FIL designation** | **Purity (%wt)** | **Chemical Structure** |
|---|---|---|---|
| $[N_{1112(OH)}]Cl$ | cholinium chloride | >99% | 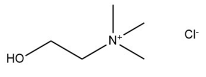 |
| $[N_{1112(OH)}][CF_3SO_3]$ | cholinium trifluoromethanesulfonate | >98% | 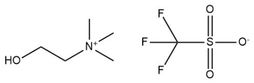 |
| $[N_{1112(OH)}][C_4F_9SO_3]$ | cholinium perfluorobutanesulfonate | >97% | 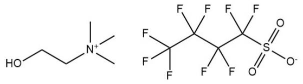 |
| $[HOC_2C_1Im]Cl$ | 1-hydroxyethyl-3-methylimidazolium chloride | >99% | 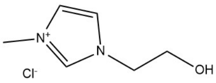 |
| $[C_2C_1Im]Cl$ | 1-ethyl-3-methylimidazolium chloride | >99% | 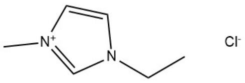 |
| $[C_4C_1Im]Cl$ | 1-butyl-3-methylimidazolium chloride | >99% | 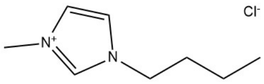 |
| $[C_2C_1Im][C_1CO_2]$ | 1-ethyl-3- methylimidazolium acetate | >95% | 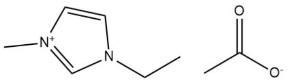 |
| $[C_4C_1Im][C_1CO_2]$ | 1-butyl-3- methylimidazolium acetate | >95% | 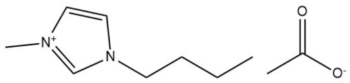 |
| $[C_2C_1Im][CF_3SO_3]$ | 1-ethyl-3-methylimidazolium trifluoromethanesulfonate | >99% | 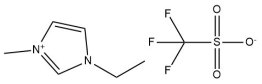 |
| $[C_4C_1Im][CF_3SO_3]$ | 1-butyl-3-methylimidazolium trifluoromethanesulfonate | >99% | 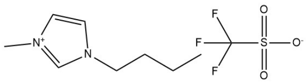 |
| $[C_2C_1Im][C_4F_9SO_3]$ | 1-ethyl-3-methylimidazolium perfluorobutanesulfonate | >98% | 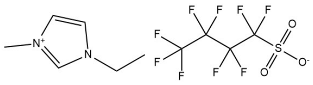 |
| $[C_2C_1Py]Br$ | 1-ethyl-3-methylpyridinium bromide | >99% | 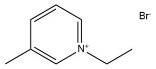 |
| $[C_2C_1Py][C_4F_9SO_3]$ | 1-ethyl-3-methylpyridinium perfluorobutanesulfonate | >99% | 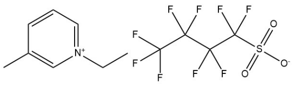 |

**Table S2.** Experimental weight fraction data for [$HOC_2C_1Im$]Cl (1) + salt (2) + $H_2O$ (3) at 25 °C.

| $K_3PO_4$ | | $K_2HPO_4$ | | | | $K_3$Citrate | |
|---|---|---|---|---|---|---|---|
| **100 w1** | **100 w2** | **100 w1** | **100 w2** | **100 w1** | **100 w2** | **100 w1** | **100 w2** |
| 64.0692 | 0.7251 | 58.6910 | 2.7615 | 31.9326 | 15.5488 | 60.7117 | 3.3249 |
| 60.9216 | 1.2762 | 54.4451 | 3.9719 | 30.9787 | 16.4006 | 60.5748 | 3.7213 |
| 58.5690 | 1.8655 | 51.1260 | 5.0015 | 29.5947 | 17.5032 | 58.0856 | 4.0734 |
| 54.2776 | 3.2092 | 47.9915 | 6.2966 | 28.1215 | 18.7286 | 54.5521 | 5.6682 |
| 51.1031 | 4.5018 | 46.2537 | 7.0706 | 26.3592 | 20.1123 | 51.4888 | 7.3143 |
| 49.6522 | 5.2668 | 42.6322 | 8.7116 | 25.1963 | 21.4065 | 50.1565 | 8.4765 |
| 47.3027 | 6.0342 | 41.0756 | 9.4884 | 23.2732 | 23.0817 | 48.3569 | 8.9393 |
| 45.8862 | 6.6417 | 38.9636 | 10.7195 | 22.1743 | 24.2511 | 42.5493 | 13.8371 |
| 44.2960 | 7.4060 | 38.9454 | 10.8562 | 20.7347 | 25.6060 | 37.5465 | 17.9859 |
| 42.3341 | 8.2780 | 38.0616 | 11.3101 | 19.2805 | 27.0321 | 35.3829 | 19.8504 |
| 40.5880 | 9.2148 | 37.2822 | 11.7532 | 18.3735 | 28.3052 | 33.4334 | 21.9731 |
| 38.5271 | 10.2728 | 36.2020 | 12.4486 | 15.8496 | 30.4238 | 30.2121 | 26.9349 |
| 35.9188 | 11.8033 | 36.0711 | 12.5616 | 13.8671 | 32.3230 | | |
| 33.4729 | 13.2721 | 35.2699 | 13.1414 | 13.1601 | 33.1410 | | |
| 30.6959 | 15.0952 | 34.3165 | 13.8300 | | | | |
| 27.7272 | 17.1431 | 33.1723 | 14.6635 | | | | |
| 24.5018 | 19.5034 | | | | | | |
| 21.5771 | 21.7003 | | | | | | |
| 18.6533 | 23.9537 | | | | | | |
| 16.9506 | 25.2923 | | | | | | |
| 15.4260 | 26.5016 | | | | | | |
| 14.6455 | 27.1331 | | | | | | |
| 13.2648 | 28.2726 | | | | | | |
| 12.2111 | 29.1708 | | | | | | |
| 54.0777 | 3.9781 | | | | | | |
| 52.1872 | 4.3807 | | | | | | |
| 49.0305 | 5.6515 | | | | | | |
| 47.3239 | 5.9924 | | | | | | |
| 46.2343 | 6.3425 | | | | | | |

**Table S3.** Experimental weight fraction data for $[C_2C_1Im]Cl$ (1) + salt (2) + $H_2O$ (3) at 25 °C.

| $K_3PO_4$ | | | | $K_2HPO_4$ | | $K_3$Citrate | |
|---|---|---|---|---|---|---|---|
| **100 w1** | **100 w2** | **100 w1** | **100 w2** | **100 w1** | **100 w2** | **100 w1** | **100 w2** |
| 43.8736 | 2.7936 | 19.3585 | 16.6193 | 52.5783 | 0.8567 | 62.6091 | 1.6204 |
| 43.2407 | 2.9671 | 18.8960 | 16.9974 | 50.3778 | 1.4058 | 59.2270 | 2.3927 |
| 41.9561 | 3.5092 | 18.3575 | 17.4705 | 46.5697 | 2.2610 | 58.4557 | 2.2043 |
| 40.7567 | 3.8300 | 17.3529 | 18.3078 | 43.1155 | 3.1371 | 58.2862 | 2.7648 |
| 39.3123 | 4.3315 | 16.6793 | 18.8968 | 40.3877 | 4.1449 | 56.5075 | 2.8338 |
| 38.1979 | 4.8266 | 16.0620 | 19.4355 | 38.2842 | 4.8523 | 54.9062 | 3.8764 |
| 37.2868 | 5.1143 | 15.4747 | 19.9530 | 36.8229 | 5.3549 | 54.8040 | 3.7840 |
| 35.7608 | 5.8247 | 14.9264 | 20.4415 | 35.4855 | 6.0173 | 54.2129 | 5.2065 |
| 34.1595 | 6.6146 | 14.3853 | 20.9280 | 34.2536 | 6.6380 | 53.7832 | 4.1610 |
| 32.7252 | 7.3220 | 13.9528 | 21.3145 | 32.5960 | 7.5641 | 51.6763 | 5.5982 |
| 31.1182 | 8.2485 | 13.5249 | 21.6877 | 30.9729 | 8.5608 | 49.6132 | 7.8382 |
| 29.9933 | 8.9165 | 13.0428 | 22.1356 | 29.5870 | 9.4705 | 49.0565 | 7.3987 |
| 28.8824 | 9.6188 | 12.5913 | 22.5542 | 28.0417 | 10.5727 | 48.3586 | 7.8215 |
| 27.8478 | 10.2886 | 12.1460 | 22.9774 | 26.4555 | 11.8000 | 48.1717 | 7.9699 |
| 26.9741 | 10.8418 | 10.8876 | 24.2086 | 24.7900 | 13.1688 | 42.4865 | 12.6533 |
| 25.9495 | 11.5567 | 10.5421 | 24.5446 | 22.9787 | 14.7426 | 32.3177 | 21.9105 |
| 24.9120 | 12.3112 | 10.0722 | 25.0302 | 20.9842 | 16.6117 | 31.3611 | 23.0489 |
| 23.9086 | 13.0464 | 9.7485 | 25.3674 | 18.3763 | 19.2268 | 29.3029 | 25.9867 |
| 22.8797 | 13.8396 | 9.2717 | 25.8940 | 16.1790 | 21.5587 | 24.8540 | 30.3331 |
| 21.9185 | 14.6135 | 8.7747 | 26.4363 | 14.7495 | 23.1103 | 22.2708 | 34.7482 |
| 21.0763 | 15.2266 | 8.2564 | 27.0325 | 12.9209 | 25.1564 | | |
| 20.4623 | 15.7208 | 7.2735 | 28.2122 | | | | |
| 19.9558 | 16.1128 | 6.4523 | 29.2794 | | | | |

**Table S4.** Experimental weight fraction data for $[C_4C_1Im]Cl$ (1) + salt (2) + $H_2O$ (3) at 25 °C.

| $K_3PO_4$ | | | | $K_2HPO_4$ | | $K_3$Citrate | |
|---|---|---|---|---|---|---|---|
| **100 w1** | **100 w2** | **100 w1** | **100 w2** | **100 w1** | **100 w2** | **100 w1** | **100 w2** |
| 41.8906 | 2.8595 | 12.5327 | 20.1084 | 40.0087 | 3.6182 | 68.6177 | 0.9278 |
| 39.9819 | 3.3058 | 12.1700 | 20.4206 | 38.6057 | 4.2896 | 65.4090 | 1.8106 |
| 38.8739 | 3.8019 | 12.1700 | 20.4206 | 37.4672 | 4.8001 | 61.2769 | 3.2535 |
| 37.8746 | 3.9618 | 11.7955 | 20.7756 | 36.4461 | 5.1206 | 57.5298 | 5.1954 |
| 37.1870 | 4.3259 | 11.4660 | 21.0652 | 35.3489 | 5.6262 | 56.0202 | 5.6722 |
| 36.0395 | 4.6903 | 11.1607 | 21.3412 | 34.3258 | 6.1259 | 52.6189 | 7.6308 |
| 35.1306 | 5.0673 | 10.8382 | 21.6389 | 33.3329 | 6.6003 | 49.5565 | 9.3554 |
| 33.8897 | 5.6076 | 10.5469 | 21.9074 | 32.4140 | 7.0013 | 45.5612 | 10.9534 |
| 33.0657 | 5.9427 | 10.2577 | 22.1749 | 31.5512 | 7.3942 | 44.1917 | 12.0111 |
| 31.9724 | 6.4234 | 9.9859 | 22.4330 | 30.4399 | 8.0651 | 43.3820 | 12.5970 |
| 31.0605 | 6.9405 | 9.7880 | 22.6352 | 28.8977 | 9.0974 | 43.2962 | 12.6158 |
| 30.3987 | 7.2366 | 9.5284 | 22.8906 | 27.9909 | 9.6470 | 42.5537 | 13.2084 |
| 29.6348 | 7.6550 | 9.2815 | 23.1380 | 27.3074 | 10.1086 | 41.7848 | 13.7265 |
| 28.8222 | 8.1629 | 9.0385 | 23.3785 | 26.5486 | 10.6481 | 41.2318 | 14.1761 |
| 28.1936 | 8.4740 | 8.8105 | 23.6072 | 25.9663 | 11.0723 | 40.5983 | 14.6456 |
| 27.4479 | 8.9329 | 8.5801 | 23.8511 | 25.3945 | 11.4868 | 40.0128 | 15.1129 |
| 26.7309 | 9.3710 | 8.2934 | 24.1492 | 24.7208 | 11.9844 | 39.3446 | 15.6226 |
| 26.0723 | 9.7662 | 8.0122 | 24.4598 | 24.0091 | 12.5532 | 38.3873 | 16.4068 |
| 25.4305 | 10.1673 | 7.5913 | 24.8410 | 23.4247 | 13.0014 | 37.2832 | 17.3129 |
| 24.8140 | 10.5459 | 7.3387 | 25.1315 | 22.7241 | 13.5790 | 36.1344 | 18.2787 |
| 24.1910 | 10.9535 | 7.1204 | 25.3747 | 22.1258 | 14.0485 | 35.0170 | 19.2801 |
| 23.5021 | 11.4619 | 6.8886 | 25.6523 | 21.4740 | 14.5958 | 33.7632 | 20.4186 |
| 22.8964 | 11.8638 | 6.6143 | 25.9843 | 20.8253 | 15.1623 | 32.9684 | 21.1321 |
| 22.2675 | 12.3190 | 6.3569 | 26.3028 | 20.1863 | 15.7344 | 32.0615 | 21.9860 |
| 21.6529 | 12.7749 | 6.1140 | 26.5984 | 19.6582 | 16.2054 | 31.7385 | 22.3436 |
| 20.8232 | 13.3813 | 5.8652 | 26.9274 | 18.9176 | 16.6169 | 30.8304 | 23.1889 |
| 20.2981 | 13.7706 | 5.6356 | 27.2293 | 17.7983 | 17.4090 | 29.6213 | 24.3944 |
| 19.4953 | 14.3944 | 5.4275 | 27.5096 | 16.6656 | 18.2870 | 23.0744 | 31.3399 |
| 18.7496 | 14.9807 | 5.2487 | 27.7692 | 15.7079 | 19.0233 | 22.7232 | 31.7418 |
| 18.0786 | 15.4778 | 5.0642 | 28.0141 | 14.7872 | 19.7642 | 22.0533 | 32.4413 |
| 17.4292 | 15.9982 | 4.7666 | 28.4411 | 13.9321 | 20.4962 | 20.9840 | 33.6580 |
| 17.0003 | 16.3515 | 4.5932 | 28.6966 | 13.1754 | 21.0743 | 20.0165 | 34.7270 |
| 16.5925 | 16.6830 | 4.2525 | 29.2191 | 12.2414 | 21.9148 | 19.1486 | 35.7560 |
| 16.2067 | 17.0009 | 4.0769 | 29.4778 | 11.4273 | 22.7275 | 18.3080 | 36.6740 |
| 15.8707 | 17.2635 | 3.8825 | 29.8300 | 10.8673 | 23.2444 | 17.6665 | 37.4760 |
| 15.3709 | 17.6775 | 3.6854 | 30.1753 | 10.2721 | 23.8326 | 17.3627 | 37.7849 |
| 14.8289 | 18.1539 | 3.5230 | 30.4525 | 9.6734 | 24.4565 | 17.1658 | 38.0226 |
| 14.0587 | 18.7824 | 3.3308 | 30.8260 | 9.0804 | 25.1608 | 16.9723 | 38.2626 |
| 13.7651 | 19.0302 | 3.1553 | 31.1503 | 8.6838 | 25.6179 | 16.6935 | 38.5719 |
| 13.3303 | 19.4168 | 2.9000 | 31.6599 | 8.2913 | 26.0929 | 16.5084 | 38.8014 |
| 12.9142 | 19.7848 | 2.6320 | 32.2400 | 7.4773 | 27.1460 | 16.2055 | 39.2175 |
| | | | | 7.0421 | 27.8285 | | |
| | | | | 6.4447 | 28.8033 | | |

**Table S5.** Experimental weight fraction data for $[C_2C_1Im][C_1CO_2]$ (1) + salt (2) + $H_2O$ (3) at 25 °C.

| $K_3PO_4$ | | | | $K_2HPO_4$ | | | |
|---|---|---|---|---|---|---|---|
| **100 w1** | **100 w2** | **100 w1** | **100 w2** | **100 w1** | **100 w2** | **100 w1** | **100 w2** |
| 33.7147 | 5.9587 | 13.3521 | 21.6403 | 64.4609 | 0.3596 | 14.8379 | 22.5164 |
| 32.6150 | 6.3794 | 12.9975 | 21.9878 | 57.2505 | 1.3167 | 14.5792 | 22.8507 |
| 31.8643 | 6.7874 | 12.6416 | 22.3357 | 37.7450 | 4.7559 | 14.1665 | 23.3591 |
| 30.9693 | 7.3683 | 12.3225 | 22.6498 | 36.1272 | 5.9770 | 13.7928 | 23.7710 |
| 30.1263 | 7.9223 | 11.9988 | 22.9719 | 35.3987 | 6.2808 | 13.5092 | 24.1547 |
| 29.2907 | 8.4752 | 11.6822 | 23.3084 | 34.3804 | 6.7697 | 13.1133 | 24.6002 |
| 28.4751 | 9.0306 | 11.4089 | 23.5783 | 33.4691 | 7.2428 | 12.7984 | 25.0119 |
| 27.7403 | 9.5291 | 11.1480 | 23.8331 | 32.1976 | 7.6913 | 12.3074 | 25.5971 |
| 27.0261 | 10.0117 | 10.8982 | 24.0749 | 28.9344 | 10.2613 | 11.7905 | 26.2095 |
| 26.2298 | 10.6063 | 10.6309 | 24.3448 | 25.4291 | 13.0812 | 11.3453 | 26.8193 |
| 25.4570 | 11.1833 | 10.4027 | 24.5780 | 21.9028 | 16.2898 | 10.8198 | 27.3932 |
| 24.8701 | 11.5697 | 10.1259 | 24.8655 | 21.5452 | 15.3799 | 10.4335 | 27.9059 |
| 24.2978 | 11.9627 | 9.8900 | 25.1101 | 21.0955 | 15.7326 | 9.9222 | 28.5973 |
| 23.2662 | 12.7565 | 9.5500 | 25.4701 | 20.6371 | 16.1145 | 9.4734 | 29.1692 |
| 22.2387 | 13.5907 | 9.1996 | 25.8473 | 20.2651 | 16.4594 | 9.0316 | 29.7308 |
| 21.1904 | 14.4816 | 8.8822 | 26.1898 | 19.9704 | 16.7785 | 8.5633 | 30.3355 |
| 20.3560 | 15.1467 | 8.5595 | 26.5535 | 19.6704 | 17.0867 | 8.2434 | 30.8433 |
| 19.7743 | 15.6265 | 8.2274 | 26.9339 | 19.3853 | 17.3948 | 7.7411 | 31.4991 |
| 19.2330 | 16.1074 | 7.9267 | 27.2846 | 19.1239 | 17.6705 | 7.3683 | 32.0542 |
| 18.5844 | 16.7010 | 7.6624 | 27.5844 | 18.9062 | 17.9036 | 6.9664 | 32.5661 |
| 18.0267 | 17.2044 | 7.4036 | 27.8825 | 18.7342 | 18.0946 | 6.5921 | 33.1762 |
| 17.4502 | 17.7401 | 7.1446 | 28.1947 | 18.3751 | 18.4321 | 6.1953 | 33.7963 |
| 16.9037 | 18.2568 | 6.8949 | 28.4892 | 17.9251 | 18.9076 | 5.8205 | 34.4383 |
| 16.4109 | 18.7148 | 6.6317 | 28.8200 | 17.5870 | 19.3054 | 5.3769 | 35.1329 |
| 15.9031 | 19.2007 | 6.3719 | 29.1610 | 17.2448 | 19.7529 | 4.9430 | 35.8293 |
| 15.4433 | 19.6276 | 6.1245 | 29.4701 | 16.8781 | 20.2144 | 4.6607 | 36.3096 |
| 14.9896 | 20.0612 | 5.7816 | 29.9388 | 16.3474 | 20.7657 | 4.5058 | 36.6721 |
| 14.5370 | 20.4902 | 5.4757 | 30.3560 | 15.8588 | 21.3207 | 4.3522 | 37.0148 |
| 14.1127 | 20.9028 | 5.1150 | 30.8848 | 15.6111 | 21.6119 | | |
| 13.7185 | 21.2851 | 4.6403 | 31.6390 | 15.2357 | 22.0713 | | |

**Table S6.** Experimental weight fraction data for $[C_4C_1Im][C_1CO_2]$ (1) + salt (2) + $H_2O$ (3) at 25 °C.

| $K_3PO_4$ | | | | | | $K_2HPO_4$ | |
|---|---|---|---|---|---|---|---|
| **100 w1** | **100 w2** | **100 w1** | **100 w2** | **100 w1** | **100 w2** | **100 w1** | **100 w2** |
| 49.5376 | 3.0609 | 16.5166 | 17.4519 | 8.9296 | 23.9939 | 64.2779 | 0.4361 |
| 43.8625 | 3.7582 | 16.1992 | 17.6013 | 8.6029 | 24.3520 | 61.5277 | 0.8338 |
| 40.7021 | 4.1819 | 15.8280 | 17.8655 | 8.3591 | 24.5756 | 58.2273 | 1.2306 |
| 40.1993 | 5.4375 | 15.5585 | 18.0744 | 8.1610 | 24.8110 | 48.3839 | 4.1522 |
| 36.5350 | 6.7122 | 15.3067 | 18.2469 | 7.9760 | 25.0316 | 46.7228 | 4.3549 |
| 36.0590 | 6.6840 | 15.0934 | 18.3936 | 7.8244 | 25.1898 | 33.0690 | 8.4837 |
| 33.9682 | 7.5799 | 14.6096 | 18.8303 | 7.6606 | 25.3799 | 31.3454 | 9.0560 |
| 33.6767 | 7.1554 | 14.3267 | 19.0436 | 7.4937 | 25.5812 | 29.6689 | 9.7626 |
| 32.3162 | 7.7480 | 14.0949 | 19.2303 | 7.2970 | 25.8491 | 27.4729 | 11.0961 |
| 31.4707 | 7.9372 | 13.8462 | 19.4543 | 7.1059 | 25.9887 | 26.0431 | 12.0258 |
| 29.6739 | 9.8532 | 13.6020 | 19.6702 | 7.0064 | 26.0709 | 24.8647 | 12.8818 |
| 29.4365 | 9.3591 | 13.3725 | 19.8685 | 6.8322 | 26.1996 | 23.7152 | 13.8556 |
| 27.4810 | 10.1152 | 13.0827 | 20.1122 | 5.6796 | 28.4240 | 21.6794 | 15.6908 |
| 26.1796 | 10.9056 | 12.8236 | 20.3689 | 5.5465 | 28.6060 | 19.0984 | 18.1273 |
| 25.6026 | 11.5046 | 12.5637 | 20.6473 | 5.3950 | 28.7198 | 18.0257 | 18.7941 |
| 25.3140 | 11.1070 | 12.2784 | 20.9600 | 5.2481 | 28.9478 | 17.1658 | 19.4190 |
| 21.5016 | 13.4967 | 12.0590 | 21.1711 | 5.0981 | 29.0634 | 14.7928 | 23.3119 |
| 21.2031 | 13.6453 | 11.8042 | 21.4399 | 4.9836 | 29.2359 | 14.1580 | 23.6487 |
| 20.4450 | 14.3774 | 11.6399 | 21.5029 | 4.8687 | 29.3483 | 13.7145 | 24.0245 |
| 20.1138 | 14.5839 | 11.4592 | 21.6098 | 4.7801 | 29.4037 | 13.2863 | 24.1039 |
| 19.6872 | 14.9197 | 11.1161 | 21.8911 | 4.6541 | 29.5399 | 12.4171 | 24.8234 |
| 19.2872 | 15.1253 | 10.8512 | 22.1335 | 4.4723 | 29.7479 | 11.7072 | 25.4608 |
| 18.6438 | 15.6527 | 10.5916 | 22.3943 | 4.2909 | 29.9818 | 11.0517 | 26.0361 |
| 18.4682 | 15.7815 | 10.3567 | 22.6417 | 4.0753 | 30.2185 | 10.6449 | 26.3284 |
| 17.9410 | 16.3757 | 10.0399 | 23.0134 | 3.8540 | 30.4992 | 9.7498 | 27.2873 |
| 17.6278 | 16.6034 | 9.7801 | 23.2937 | 3.6335 | 30.8125 | 8.5634 | 28.6167 |
| 17.2490 | 16.8341 | 9.4793 | 23.5039 | 3.4342 | 31.0638 | 6.3289 | 31.4714 |
| 16.9623 | 17.0224 | 9.2078 | 23.7267 | 3.2789 | 31.2619 | 3.1585 | 35.6491 |

**Table S7.** Experimental weight fraction data for $[C_2C_1Im][CF_3SO_3]$ (1) + salt (2) + $H_2O$ (3) at 25 °C.

| $K_3PO_4$ | | | | | | $K_2HPO_4$ (cont.) | |
|---|---|---|---|---|---|---|---|
| **100 w1** | **100 w2** | **100 w1** | **100 w2** | **100 w1** | **100 w2** | **100 w1** | **100 w2** |
| 50.8886 | 2.6079 | 31.2897 | 7.0667 | 16.3921 | 12.1435 | 55.0085 | 2.3889 |
| 49.8747 | 2.7810 | 30.5183 | 7.3648 | 15.8742 | 12.3286 | 53.3473 | 2.6608 |
| 48.8190 | 2.9531 | 29.8585 | 7.5228 | 15.3503 | 12.5628 | 51.7940 | 2.9055 |
| 47.7844 | 3.1329 | 29.2630 | 7.6275 | 14.8213 | 12.8580 | 50.1866 | 3.2407 |
| 47.0413 | 3.2968 | 28.5613 | 7.9044 | 14.3027 | 13.1563 | 48.7463 | 3.4478 |
| 46.3425 | 3.4580 | 27.9417 | 8.0335 | 13.7978 | 13.3472 | 47.3070 | 3.6525 |
| 45.4704 | 3.5884 | 27.0212 | 8.3856 | 13.1271 | 13.7442 | 45.4085 | 4.0967 |
| 44.7886 | 3.7343 | 26.4922 | 8.4993 | 12.4133 | 14.1467 | 44.2309 | 4.3049 |
| 44.2638 | 3.7883 | 25.8995 | 8.6603 | 11.7822 | 14.5529 | 42.8038 | 4.7428 |
| 43.6999 | 3.9229 | 25.2711 | 8.8643 | 11.1692 | 14.9108 | 41.6915 | 4.9112 |
| 43.1294 | 4.0619 | 24.8053 | 9.0268 | 10.9146 | 14.8956 | 40.3666 | 5.3171 |
| 42.2608 | 4.2531 | 24.2717 | 9.1604 | 10.5571 | 15.3573 | 39.1664 | 5.6976 |
| 41.7110 | 4.4058 | 23.5815 | 9.3981 | 10.0631 | 15.6898 | 38.2672 | 5.8403 |
| 41.1194 | 4.5560 | 22.9230 | 9.6067 | 9.6708 | 15.9465 | 37.1244 | 6.1710 |
| 40.5952 | 4.6709 | 22.3258 | 9.8088 | 9.2275 | 16.3480 | 36.5053 | 6.3230 |
| 40.0057 | 4.8151 | 21.8209 | 9.9271 | 8.8521 | 16.6930 | 35.6853 | 6.6563 |
| 39.4798 | 4.9218 | 21.2068 | 10.2190 | 8.5098 | 16.9180 | 35.1370 | 6.7793 |
| 38.4463 | 5.1784 | 20.6615 | 10.3494 | 8.0981 | 17.2570 | 34.5356 | 6.9337 |
| 37.4058 | 5.4807 | 20.1768 | 10.5203 | 7.7403 | 17.5861 | 33.6722 | 7.2169 |
| 36.4765 | 5.7253 | 19.6992 | 10.7233 | 7.3496 | 18.1176 | 32.9503 | 7.5180 |
| 35.9875 | 5.8509 | 19.2457 | 10.8921 | 7.1174 | 18.3061 | 32.1090 | 7.7707 |
| 35.1151 | 6.0741 | 18.7264 | 11.1080 | 6.8203 | 18.6144 | 31.3517 | 8.0019 |
| 34.2834 | 6.2851 | 18.2595 | 11.2699 | 6.5435 | 18.8928 | 30.6119 | 8.2326 |
| 33.4997 | 6.4836 | 17.8304 | 11.4713 | 6.2814 | 19.0847 | 29.8648 | 8.4482 |
| 32.7553 | 6.6585 | 17.4265 | 11.6628 | 5.9572 | 19.6711 | 29.1842 | 8.6690 |
| 32.0051 | 6.8665 | 16.8964 | 11.9335 | 5.6474 | 20.0350 | 28.5156 | 8.8684 |

**Table S8.** Experimental weight fraction data for $[C_2C_1Im][CF_3SO_3]$ (1) + salt (2) + $H_2O$ (3) at 25 °C (continuation).

| $K_2HPO_4$ (cont.) | | | | $K_3$Citrate | | | |
|---|---|---|---|---|---|---|---|
| **100 w1** | **100 w2** | **100 w1** | **100 w2** | **100 w1** | **100 w2** | **100 w1** | **100 w2** |
| 27.6391 | 9.1764 | 11.3141 | 16.9545 | 53.4904 | 4.4396 | 27.0110 | 13.5644 |
| 26.7096 | 9.5644 | 10.9826 | 17.3075 | 51.9314 | 4.7985 | 26.3998 | 13.8562 |
| 26.0467 | 9.7041 | 10.7404 | 17.4504 | 50.4261 | 5.1283 | 25.8267 | 14.1217 |
| 25.1883 | 10.1117 | 10.5032 | 17.6446 | 49.0406 | 5.4602 | 25.2063 | 14.2979 |
| 24.5003 | 10.3381 | 10.2944 | 17.7977 | 47.9888 | 5.7994 | 24.6349 | 14.5627 |
| 23.8437 | 10.5476 | 10.0496 | 18.0294 | 46.9241 | 6.1345 | 24.1270 | 14.8103 |
| 23.0455 | 10.8533 | 9.7961 | 18.2131 | 45.9246 | 6.4589 | 23.0678 | 15.4041 |
| 22.3950 | 11.1440 | 9.5952 | 18.3740 | 44.9886 | 6.7651 | 22.4689 | 15.6953 |
| 21.7234 | 11.4188 | 9.3671 | 18.6066 | 44.1131 | 7.0371 | 21.7141 | 16.2425 |
| 21.0771 | 11.6357 | 9.0349 | 18.9473 | 43.1320 | 7.4152 | 21.1851 | 16.4965 |
| 20.3926 | 11.9550 | 8.6968 | 19.3124 | 42.3224 | 7.6530 | 20.6671 | 16.7791 |
| 19.7435 | 12.2357 | 8.3208 | 19.6705 | 41.5166 | 7.9183 | 20.1601 | 17.1025 |
| 19.1153 | 12.5536 | 7.9619 | 20.0607 | 40.6736 | 8.2520 | 19.7997 | 17.1889 |
| 18.6083 | 12.7345 | 7.6319 | 20.4921 | 39.9076 | 8.5198 | 19.3551 | 17.5284 |
| 18.0737 | 12.9890 | 7.3690 | 20.6965 | 39.1250 | 8.7936 | 18.9841 | 17.7617 |
| 17.5131 | 13.2970 | 7.0157 | 21.0773 | 38.4277 | 9.0150 | 18.5823 | 17.9753 |
| 17.0356 | 13.5311 | 6.6295 | 21.5643 | 37.6431 | 9.3427 | 18.1176 | 18.3058 |
| 16.6359 | 13.6673 | 6.3299 | 21.9929 | 36.9242 | 9.5693 | 17.5848 | 18.7822 |
| 16.2514 | 13.9118 | | | 36.2391 | 9.8483 | 17.1830 | 18.9277 |
| 15.8125 | 14.1701 | | | 35.5721 | 10.0510 | 16.7735 | 19.2382 |
| 15.3875 | 14.3975 | | | 34.9599 | 10.2754 | 16.3184 | 19.6190 |
| 14.9515 | 14.6169 | | | 34.1488 | 10.7611 | 15.8517 | 19.9673 |
| 14.4568 | 14.9440 | | | 33.4384 | 10.9190 | 15.4478 | 20.2186 |
| 14.1310 | 15.0935 | | | 32.5321 | 11.3269 | 15.1110 | 20.4561 |
| 13.7743 | 15.2763 | | | 31.8710 | 11.4806 | 14.5635 | 21.0149 |
| 13.2934 | 15.5754 | | | 31.0495 | 11.8622 | 14.1313 | 21.3571 |
| 12.8684 | 15.9019 | | | 30.2820 | 12.2018 | 13.5769 | 21.8776 |
| 12.5071 | 16.1324 | | | 29.5428 | 12.5525 | 12.6798 | 22.7646 |
| 12.2171 | 16.3392 | | | 28.9874 | 12.6300 | 11.4910 | 24.0109 |
| 11.9363 | 16.5365 | | | 28.3019 | 12.9594 | 10.9765 | 24.5973 |
| 11.6126 | 16.7729 | | | 27.6524 | 13.2792 | 10.1121 | 25.5868 |

**Table S9.** Experimental weight fraction data for $[C_4C_1Im][CF_3SO_3]$ (1) + salt (2) + $H_2O$ (3) at 25 °C.

| $K_3PO_4$ | | | | $K_2HPO_4$ | | | |
|---|---|---|---|---|---|---|---|
| **100 w1** | **100 w2** | **100 w1** | **100 w2** | **100 w1** | **100 w2** | **100 w1** | **100 w2** |
| 51.6391 | 1.2330 | 24.5425 | 3.9306 | 57.4577 | 1.0303 | 22.2213 | 4.8832 |
| 50.3521 | 1.3259 | 23.9088 | 4.0241 | 55.5480 | 1.1647 | 21.6290 | 4.9723 |
| 49.1749 | 1.4027 | 23.3086 | 4.1142 | 53.6024 | 1.2875 | 20.8830 | 5.1744 |
| 47.7770 | 1.5738 | 22.7503 | 4.2023 | 51.6590 | 1.4167 | 20.2576 | 5.3267 |
| 45.9602 | 1.7001 | 22.2050 | 4.2876 | 49.2137 | 1.5957 | 19.5906 | 5.4624 |
| 44.9028 | 1.7675 | 21.5438 | 4.4735 | 46.8916 | 1.8229 | 18.8398 | 5.7275 |
| 43.8990 | 1.8313 | 21.0063 | 4.5832 | 44.7387 | 2.0054 | 18.2080 | 5.8730 |
| 42.9409 | 1.8949 | 20.5292 | 4.6931 | 43.0330 | 2.2095 | 17.5606 | 6.0780 |
| 41.8119 | 2.0446 | 20.0322 | 4.8075 | 41.6879 | 2.3014 | 16.8355 | 6.3507 |
| 40.8471 | 2.1073 | 19.4597 | 4.9494 | 40.2269 | 2.4946 | 16.0998 | 6.6353 |
| 39.8098 | 2.2124 | 18.9364 | 5.0771 | 38.9778 | 2.5739 | 15.4408 | 6.8984 |
| 38.9810 | 2.2600 | 18.3680 | 5.2368 | 37.6792 | 2.7502 | 14.7695 | 7.1832 |
| 37.9430 | 2.3896 | 17.8367 | 5.3883 | 36.5987 | 2.8149 | 13.9943 | 7.5427 |
| 37.1070 | 2.4415 | 17.3145 | 5.5558 | 35.4202 | 2.9814 | 13.2816 | 7.9234 |
| 36.0785 | 2.5737 | 16.6295 | 5.7710 | 34.1416 | 3.1208 | 12.6346 | 8.2858 |
| 35.1367 | 2.6707 | 15.9991 | 5.9740 | 32.9186 | 3.2396 | 11.6648 | 8.8893 |
| 34.2779 | 2.7616 | 15.3383 | 6.2320 | 31.7771 | 3.3570 | 10.9260 | 9.4050 |
| 33.4042 | 2.8538 | 14.7792 | 6.4336 | 30.4973 | 3.5582 | 9.9885 | 10.1150 |
| 32.4575 | 2.9338 | 14.1688 | 6.6895 | 29.6815 | 3.6736 | | |
| 31.6528 | 3.0227 | 13.5695 | 6.9676 | 28.7189 | 3.7708 | | |
| 30.7545 | 3.1018 | 12.9895 | 7.2525 | 27.6882 | 3.9356 | | |
| 29.8014 | 3.2314 | 12.4036 | 7.5546 | 26.8743 | 4.0099 | | |
| 28.8002 | 3.3522 | 11.8733 | 7.8347 | 26.1181 | 4.1760 | | |
| 28.1672 | 3.4223 | 11.2173 | 8.2394 | 25.3178 | 4.2863 | | |
| 27.4398 | 3.4867 | 10.8063 | 8.4398 | 24.5432 | 4.4057 | | |
| 26.6560 | 3.6062 | 10.2893 | 8.8533 | 23.7772 | 4.5367 | | |
| 25.9126 | 3.7190 | 9.5992 | 9.3546 | 22.9641 | 4.7229 | | |
| 25.2799 | 3.7889 | | | | | | |

**Table S10.** Experimental weight fraction data for $[C_4C_1Im][CF_3SO_3]$(1)+salt or carbohydrate(2)+$H_2O$(3) at 25 °C.

| **$K_3$Citrate** | | | | **D-glucose** | | **sucrose** | |
|---|---|---|---|---|---|---|---|
| **100 w1** | **100 w2** | **100 w1** | **100 w2** | **100 w1** | **100 w2** | **100 w1** | **100 w2** |
| 60.6584 | 1.2207 | 30.9311 | 4.8549 | 68.0542 | 5.7661 | 44.0485 | 14.6802 |
| 59.0553 | 1.3625 | 30.1871 | 5.0037 | 63.7937 | 6.4358 | 41.7608 | 15.6231 |
| 57.5596 | 1.4967 | 29.3776 | 5.1302 | 61.7596 | 6.8919 | 39.3705 | 16.8676 |
| 56.0684 | 1.6190 | 28.5090 | 5.3245 | 59.0517 | 7.5297 | 37.6154 | 17.6697 |
| 54.6630 | 1.7446 | 27.7808 | 5.4403 | 56.8067 | 8.1829 | 36.1079 | 18.4102 |
| 53.3258 | 1.8538 | 27.0187 | 5.6148 | 54.7842 | 8.7489 | 34.3204 | 19.3561 |
| 52.0961 | 1.9635 | 26.2813 | 5.7759 | 52.9062 | 9.2649 | 32.6629 | 20.2228 |
| 50.9151 | 2.0577 | 25.4978 | 5.9892 | 50.8272 | 9.9630 | 31.2445 | 20.9487 |
| 49.4464 | 2.3116 | 24.8371 | 6.1332 | 48.9188 | 10.6149 | 29.2586 | 22.0844 |
| 48.3634 | 2.4027 | 24.1392 | 6.3222 | 47.2480 | 11.1959 | 27.3712 | 23.1617 |
| 47.3753 | 2.4734 | 23.5639 | 6.4367 | 45.6171 | 11.7999 | 25.6644 | 24.1839 |
| 46.0857 | 2.7032 | 22.9451 | 6.5994 | 44.0248 | 12.3438 | 24.6548 | 24.9108 |
| 45.1301 | 2.7885 | 22.2100 | 6.8736 | 42.2454 | 13.0436 | 23.5264 | 25.7243 |
| 44.0371 | 2.9607 | 21.5484 | 7.1038 | 40.8562 | 13.5160 | 21.9871 | 27.1210 |
| 43.1602 | 3.0304 | 20.8845 | 7.3187 | 39.3309 | 14.1092 | 20.4045 | 28.5091 |
| 42.2005 | 3.1686 | 20.3061 | 7.4813 | 37.3799 | 14.8451 | 18.3546 | 30.5272 |
| 41.2199 | 3.3347 | 19.6634 | 7.7071 | 36.0157 | 15.3370 | | |
| 40.4689 | 3.3857 | 19.0439 | 7.9858 | 34.6456 | 15.9303 | | |
| 39.5941 | 3.5232 | 18.3791 | 8.2687 | 33.1655 | 16.4147 | | |
| 38.5465 | 3.6611 | 17.7562 | 8.5347 | 31.6583 | 17.0310 | | |
| 37.3806 | 3.8827 | 17.1158 | 8.8391 | 30.1380 | 17.6960 | | |
| 36.4664 | 3.9970 | 16.4551 | 9.1810 | 29.2005 | 18.1080 | | |
| 35.5797 | 4.1075 | 15.7568 | 9.5841 | 28.3323 | 18.4823 | | |
| 34.7334 | 4.2196 | 14.9607 | 10.0635 | 27.4302 | 18.9056 | | |
| 33.7849 | 4.4098 | 14.2012 | 10.5818 | 26.5675 | 19.3121 | | |
| 32.9819 | 4.5325 | 13.4113 | 11.1555 | 25.7687 | 19.7216 | | |
| 32.2696 | 4.6088 | 12.2743 | 12.1154 | 24.8199 | 20.2364 | | |
| 31.6921 | 4.7130 | | | 23.5388 | 20.9381 | | |

**Table S11.** Experimental weight fraction data for $[C_2C_1Im][C_4F_9SO_3]$ (1) + salt(2) + $H_2O$(3) at 25 °C.

| $K_3PO_4$ | | | | $K_2HPO_4$ | | | |
|---|---|---|---|---|---|---|---|
| **100 w1** | **100 w2** | **100 w1** | **100 w2** | **100 w1** | **100 w2** | **100 w1** | **100 w2** |
| 67.0734 | 0.1799 | 34.1986 | 0.9683 | 60.9131 | 0.2870 | 34.1439 | 1.1513 |
| 63.9977 | 0.2056 | 33.1878 | 1.0237 | 59.1142 | 0.3085 | 33.1381 | 1.2226 |
| 61.0896 | 0.2288 | 32.1075 | 1.0923 | 57.5613 | 0.3281 | 32.4498 | 1.2657 |
| 58.8221 | 0.2524 | 31.3331 | 1.1456 | 55.9842 | 0.3523 | 31.4644 | 1.3322 |
| 57.0255 | 0.2819 | 30.6939 | 1.1832 | 54.0247 | 0.4019 | 30.3951 | 1.4209 |
| 54.9614 | 0.3149 | 29.9747 | 1.2333 | 52.5238 | 0.4251 | 29.3662 | 1.4915 |
| 53.0235 | 0.3401 | 29.0032 | 1.3012 | 50.9097 | 0.4769 | 28.3941 | 1.5758 |
| 51.6575 | 0.3638 | 28.2495 | 1.3608 | 49.5525 | 0.5225 | 27.2975 | 1.6721 |
| 49.6430 | 0.4092 | 27.5038 | 1.4217 | 47.9607 | 0.5624 | 26.1396 | 1.7800 |
| 48.3646 | 0.4315 | 26.7965 | 1.4729 | 46.7681 | 0.5813 | 24.6908 | 1.9232 |
| 46.9012 | 0.4819 | 25.7864 | 1.5583 | 45.6657 | 0.6229 | 23.2515 | 2.0760 |
| 45.2571 | 0.5263 | 24.7638 | 1.6521 | 44.8428 | 0.6420 | | |
| 44.3834 | 0.5393 | 24.0341 | 1.7196 | 44.0553 | 0.6764 | | |
| 43.6917 | 0.5596 | 23.0985 | 1.8071 | 43.0557 | 0.7155 | | |
| 42.8229 | 0.5965 | 22.0362 | 1.9150 | 42.0999 | 0.7563 | | |
| 42.1297 | 0.6164 | 20.8880 | 2.0397 | 41.1599 | 0.7927 | | |
| 41.5187 | 0.6327 | 19.7431 | 2.1677 | 40.7533 | 0.8094 | | |
| 40.6686 | 0.6715 | 18.2581 | 2.3461 | 40.3645 | 0.8236 | | |
| 39.6177 | 0.7045 | | | 39.8906 | 0.8484 | | |
| 38.4673 | 0.7561 | | | 39.4521 | 0.8733 | | |
| 37.7276 | 0.7919 | | | 38.5226 | 0.9181 | | |
| 37.0047 | 0.8253 | | | 37.6568 | 0.9596 | | |
| 36.2908 | 0.8586 | | | 36.7246 | 1.0105 | | |
| 35.6433 | 0.8892 | | | 35.8722 | 1.0536 | | |
| 34.9897 | 0.9202 | | | 34.9777 | 1.1055 | | |

**Table S12.** Experimental weight fraction data for $[C_2C_1Im][C_4F_9SO_3]$)+salt or carbohydrate(2)+$H_2O$(3) at 25 °C.

| $K_3$Citrate | | | | | | $[N_{1112(OH)}][H_2PO_4]$ | |
|---|---|---|---|---|---|---|---|
| **100 w1** | **100 w2** | **100 w1** | **100 w2** | **100 w1** | **100 w2** | **100 w1** | **100 w2** |
| 69.3584 | 0.2385 | 52.1212 | 0.5475 | 43.7778 | 0.8956 | 71.2034 | 1.0773 |
| 66.1879 | 0.2692 | 51.2352 | 0.5684 | 42.7725 | 0.9539 | 64.4969 | 1.2404 |
| 62.8602 | 0.3012 | 49.8899 | 0.6183 | 41.1686 | 1.0516 | 57.0785 | 1.3781 |
| 61.1375 | 0.3397 | 48.9197 | 0.6428 | 40.2988 | 1.1085 | 53.2272 | 1.5600 |
| 59.5655 | 0.3594 | 48.1465 | 0.6876 | 39.6444 | 1.1527 | 50.4243 | 1.7238 |
| 57.3760 | 0.4179 | 47.3687 | 0.7273 | 37.9975 | 1.2724 | 46.8205 | 1.9545 |
| 56.2506 | 0.4458 | 46.6704 | 0.7580 | 36.8605 | 1.3579 | 42.9811 | 2.2695 |
| 55.1911 | 0.4612 | 46.1124 | 0.7868 | | | 41.2743 | 2.4138 |
| 53.7578 | 0.5045 | 45.0326 | 0.8348 | | | 36.6504 | 2.9723 |

**Table S13.** Experimental weight fraction data for $[C_2C_1Im][C_4F_9SO_3]$(1)+carbohydrate(2)+$H_2O$(3) at 25 °C.

| **sucrose** | | | | | | **D-glucose** | |
|---|---|---|---|---|---|---|---|
| **100 w1** | **100 w2** | **100 w1** | **100 w2** | **100 w1** | **100 w2** | **100 w1** | **100 w2** |
| 73.6181 | 4.2230 | 22.8928 | 26.7674 | 11.8479 | 33.6776 | 66.3437 | 6.8144 |
| 69.1293 | 5.3734 | 21.9879 | 27.1740 | 11.3493 | 34.1291 | 57.7611 | 9.9901 |
| 64.4455 | 6.8218 | 21.3402 | 27.5484 | 10.9476 | 34.4312 | 52.6374 | 11.9595 |
| 60.2470 | 8.6771 | 20.5174 | 28.0228 | 10.50275 | 34.82687 | 46.3800 | 14.3971 |
| 57.2167 | 9.9599 | 19.6252 | 28.5518 | 10.12422 | 35.12123 | 41.0286 | 16.5870 |
| 51.9941 | 12.4876 | 19.0897 | 28.8927 | 9.788693 | 35.38466 | 37.9954 | 17.8356 |
| 49.1097 | 13.8082 | 18.4264 | 29.2558 | 9.307898 | 35.77819 | 35.5705 | 18.8274 |
| 46.5459 | 14.9972 | 17.7863 | 29.6443 | 8.94533 | 36.09242 | 33.0919 | 19.8727 |
| 44.1989 | 16.1036 | 17.0784 | 30.0978 | 8.655774 | 36.36196 | 31.0098 | 20.7492 |
| 41.1491 | 17.6338 | 16.4678 | 30.4783 | 8.304012 | 36.72493 | 28.7985 | 21.7469 |
| 39.2673 | 18.4827 | 15.9812 | 30.7989 | 7.91576 | 37.17296 | 27.1289 | 22.4658 |
| 36.5419 | 19.8833 | 15.5827 | 31.0429 | 7.526402 | 37.64713 | 25.6903 | 23.0910 |
| 34.3661 | 20.9179 | 15.0865 | 31.3574 | 7.222182 | 37.84561 | 24.1395 | 23.8206 |
| 32.3723 | 21.8028 | 14.6329 | 31.7052 | 6.836664 | 38.2801 | 22.4307 | 24.6972 |
| 30.6322 | 22.6647 | 14.1355 | 32.0321 | 6.422921 | 38.77917 | 20.3458 | 25.7388 |
| 28.8530 | 23.6576 | 13.7601 | 32.3025 | 6.010203 | 39.21311 | 17.8538 | 26.9797 |
| 27.4487 | 24.3317 | 13.4188 | 32.5191 | 5.344232 | 40.28328 | 15.7982 | 28.1802 |
| 26.0397 | 25.0895 | 13.0396 | 32.8015 | 5.042588 | 40.65576 | 13.7945 | 29.3183 |
| 24.7942 | 25.7481 | 12.5674 | 33.1344 | | | 12.6282 | 29.9651 |
| 23.6876 | 26.3055 | 12.2268 | 33.3843 | | | 9.7764 | 32.1870 |

**Table S14.** Experimental weight fraction data for $[C_2C_1Py]Br$ (1) + salt (2) + $H_2O$ (3) at 25 °C.

| $K_3PO_4$ | | | | $K_2HPO_4$ | | | | $K_3$Citrate | |
|---|---|---|---|---|---|---|---|---|---|
| **100 w1** | **100 w2** | **100 w1** | **100 w2** | **100 w1** | **100 w2** | **100 w1** | **100 w2** | **100 w1** | **100 w2** |
| 45.1303 | 3.3374 | 18.9498 | 14.9754 | 49.3596 | 2.1114 | 15.8674 | 19.0686 | 58.7831 | 3.9822 |
| 42.9513 | 3.5392 | 18.3028 | 15.3983 | 44.9698 | 3.1027 | 15.2668 | 19.5411 | 56.3222 | 4.4001 |
| 41.3284 | 3.9371 | 17.6985 | 15.7885 | 44.0815 | 3.4404 | 15.0342 | 19.7272 | 53.4089 | 5.7731 |
| 40.4825 | 4.1994 | 17.1317 | 16.1650 | 42.8321 | 3.8061 | 14.7327 | 19.9811 | 50.9875 | 6.8678 |
| 39.7150 | 4.3839 | 16.5142 | 16.5490 | 38.9479 | 4.9953 | 14.4347 | 20.2246 | 48.3876 | 8.1121 |
| 38.0800 | 5.3899 | 15.8985 | 16.9506 | 37.7421 | 5.4650 | 14.0270 | 20.5337 | 44.0567 | 10.5960 |
| 36.8312 | 5.4853 | 15.3206 | 17.3312 | 37.3946 | 5.6016 | 13.6913 | 20.8375 | 40.5697 | 12.9628 |
| 35.5436 | 6.0453 | 14.9679 | 17.5799 | 35.7933 | 6.3296 | 12.6661 | 21.7171 | 37.6104 | 14.9742 |
| 34.5030 | 6.4010 | 14.3597 | 17.9941 | 34.3476 | 6.9550 | 12.2771 | 22.0629 | 34.2384 | 17.4888 |
| 33.3721 | 6.9046 | 13.9289 | 18.3100 | 33.6160 | 7.2930 | 11.8171 | 22.4777 | 29.9723 | 20.7688 |
| 32.5017 | 7.3873 | 13.4498 | 18.6510 | 32.7289 | 7.8166 | 11.3475 | 22.9035 | 27.4127 | 22.7383 |
| 31.5978 | 7.9053 | 13.0359 | 18.9506 | 31.7379 | 8.3050 | 11.0167 | 23.2204 | 24.9534 | 24.7075 |
| 30.7943 | 8.1731 | 12.5812 | 19.3149 | 30.6456 | 8.9095 | 10.7126 | 23.5191 | 22.6999 | 26.5685 |
| 29.8655 | 8.6055 | 12.0660 | 19.7066 | 29.6435 | 9.4650 | 10.3720 | 23.8603 | 20.4431 | 28.4993 |
| 28.8053 | 9.1869 | 11.5773 | 20.0975 | 28.4218 | 10.2569 | 10.0615 | 24.1859 | 17.4772 | 31.2771 |
| 27.8010 | 9.7509 | 11.1211 | 20.4695 | 27.6344 | 10.6428 | 9.7978 | 24.4237 | | |
| 26.8297 | 10.3303 | 10.6781 | 20.8433 | 26.5432 | 11.3248 | 9.5196 | 24.7341 | | |
| 25.9669 | 10.8084 | | | 25.3182 | 12.1129 | 9.162667 | 25.11003 | | |
| 25.1633 | 11.2517 | | | 24.3388 | 12.8006 | 8.671642 | 25.66839 | | |
| 24.4164 | 11.6593 | | | 23.1696 | 13.6201 | 8.087715 | 26.33655 | | |
| 23.7020 | 12.0589 | | | 22.3732 | 14.2188 | 7.80855 | 26.66258 | | |
| 23.0295 | 12.4529 | | | 21.3281 | 14.9651 | 7.393966 | 27.19272 | | |
| 22.2849 | 12.9270 | | | 20.3531 | 15.6765 | 6.917106 | 27.79912 | | |
| 21.6729 | 13.2788 | | | 19.3408 | 16.3794 | 6.52382 | 28.33958 | | |
| 21.0176 | 13.7039 | | | 18.4857 | 17.0243 | 6.152447 | 28.86116 | | |
| 20.4487 | 14.0390 | | | 17.8743 | 17.4637 | 5.400522 | 29.936 | | |
| 20.0039 | 14.3039 | | | 17.0889 | 18.0793 | 4.730756 | 31.0823 | | |
| 19.5743 | 14.5712 | | | 16.3805 | 18.6556 | | | | |

**Table S15.** Experimental weight fraction data for $[C_2C_1py][C_4F_9SO_3]$ (1) + salt (2) + $H_2O$ (3) at 25 °C.

| $K_3PO_4$ | | | | | | $K_2HPO_4$ | |
|---|---|---|---|---|---|---|---|
| **100 w1** | **100 w2** | **100 w1** | **100 w2** | **100 w1** | **100 w2** | **100 w1** | **100 w2** |
| 60.4076 | 0.1079 | 32.3213 | 0.5197 | 19.3682 | 1.1809 | 62.0357 | 0.1554 |
| 57.3410 | 0.1223 | 32.0724 | 0.5307 | 19.0296 | 1.2120 | 56.2688 | 0.1732 |
| 55.7904 | 0.1389 | 31.7871 | 0.5413 | 18.6898 | 1.2406 | 53.4451 | 0.2028 |
| 52.8160 | 0.1627 | 31.2050 | 0.5635 | 18.4657 | 1.2564 | 50.7185 | 0.2269 |
| 50.6311 | 0.1806 | 30.6261 | 0.5837 | 18.0921 | 1.2900 | 48.6808 | 0.2374 |
| 48.7278 | 0.1973 | 30.2419 | 0.5943 | 17.6846 | 1.3252 | 47.1283 | 0.2602 |
| 46.6352 | 0.2132 | 29.7733 | 0.6143 | 17.2328 | 1.3685 | 45.8784 | 0.2879 |
| 45.1169 | 0.2280 | 29.2220 | 0.6336 | 16.7194 | 1.4121 | 44.4791 | 0.3025 |
| 44.0094 | 0.2422 | 28.6129 | 0.6649 | 16.3276 | 1.4479 | 43.3693 | 0.3285 |
| 43.2305 | 0.2597 | 28.0721 | 0.6833 | 15.9253 | 1.4906 | 42.2941 | 0.3508 |
| 42.4762 | 0.2778 | 27.5359 | 0.7056 | 15.5722 | 1.5221 | 41.2815 | 0.3712 |
| 41.6186 | 0.2951 | 27.2121 | 0.7250 | 15.2805 | 1.5522 | 40.1251 | 0.3913 |
| 40.6257 | 0.3092 | 26.7693 | 0.7411 | 14.9635 | 1.5856 | 38.8828 | 0.4376 |
| 39.9348 | 0.3199 | 26.3236 | 0.7663 | 14.6378 | 1.6236 | 37.8746 | 0.4517 |
| 39.1989 | 0.3330 | 25.8219 | 0.7880 | 14.3338 | 1.6597 | 36.8752 | 0.4917 |
| 38.3882 | 0.3567 | 25.1921 | 0.8283 | 13.9421 | 1.7098 | 36.1328 | 0.5078 |
| 37.6847 | 0.3697 | 24.7460 | 0.8484 | 13.5620 | 1.7530 | 35.5749 | 0.5287 |
| 37.0045 | 0.3818 | 24.2437 | 0.8751 | 13.2102 | 1.7983 | 35.0316 | 0.5488 |
| 36.2557 | 0.4084 | 23.8350 | 0.9004 | 12.8293 | 1.8498 | 34.5157 | 0.5655 |
| 35.5397 | 0.4196 | 23.4746 | 0.9209 | 12.4101 | 1.9055 | 34.0125 | 0.5810 |
| 34.8767 | 0.4464 | 22.9023 | 0.9556 | 12.0578 | 1.9580 | 33.4934 | 0.6006 |
| 34.2162 | 0.4559 | 22.3136 | 0.9894 | 11.6121 | 2.0273 | 33.0348 | 0.6159 |
| 33.8996 | 0.4710 | 21.7757 | 1.0179 | 11.0848 | 2.1089 | 32.5556 | 0.6335 |
| 33.5863 | 0.4835 | 21.1806 | 1.0576 | 10.6342 | 2.1885 | 32.1496 | 0.6489 |
| 33.0754 | 0.4994 | 20.5671 | 1.1014 | 10.0551 | 2.2934 | 31.7365 | 0.6626 |
| 32.6011 | 0.5086 | 19.9829 | 1.1376 | 9.6600 | 2.3696 | 31.1899 | 0.6953 |

**Table S16.** Experimental weight fraction data for $[C_2C_1py][C_4F_9SO_3]$ (1) + salt(2) + $H_2O$(3) at 25 °C (continuing).

| $K_2HPO_4$ (cont.) | | | | $K_3$Citrate | | | |
|---|---|---|---|---|---|---|---|
| **100 w1** | **100 w2** | **100 w1** | **100 w2** | **100 w1** | **100 w2** | **100 w1** | **100 w2** |
| 30.4707 | 0.7324 | 15.6420 | 1.7995 | 52.1556 | 0.2651 | 35.7465 | 0.6626 |
| 29.9350 | 0.7449 | 15.3252 | 1.8395 | 50.7781 | 0.2807 | 35.4106 | 0.6737 |
| 29.2638 | 0.7759 | 15.1296 | 1.8608 | 49.5852 | 0.2979 | 34.9332 | 0.6983 |
| 28.6602 | 0.8042 | 14.9163 | 1.8841 | 48.3328 | 0.3094 | 34.6380 | 0.7109 |
| 28.1491 | 0.8345 | 14.5019 | 1.9369 | 47.2610 | 0.3273 | 34.1728 | 0.7335 |
| 27.4683 | 0.8693 | 14.2323 | 1.9710 | 46.6932 | 0.3435 | 33.7343 | 0.7561 |
| 26.7981 | 0.9045 | 13.9573 | 2.0089 | 45.9310 | 0.3581 | 33.4710 | 0.7669 |
| 26.1460 | 0.9416 | 13.7450 | 2.0378 | 45.0245 | 0.3731 | 33.0419 | 0.7892 |
| 25.6419 | 0.9683 | 13.4714 | 2.0759 | 44.2745 | 0.3879 | 32.3986 | 0.8237 |
| 25.0711 | 1.0023 | 13.1996 | 2.1151 | 43.4897 | 0.4156 | 31.9807 | 0.8447 |
| 24.4970 | 1.0335 | 13.0639 | 2.1360 | 42.8019 | 0.4307 | 31.3758 | 0.8775 |
| 23.8170 | 1.0820 | 12.9083 | 2.1575 | 42.1361 | 0.4450 | 30.7585 | 0.9106 |
| 23.2541 | 1.1155 | 12.6429 | 2.1973 | 41.5590 | 0.4583 | 30.2784 | 0.9439 |
| 22.4294 | 1.1797 | 12.5151 | 2.2161 | 40.7839 | 0.4837 | 29.8348 | 0.9723 |
| 21.7115 | 1.2378 | 12.3361 | 2.2475 | 40.2993 | 0.4969 | 29.3366 | 0.9978 |
| 21.1017 | 1.2788 | 12.0381 | 2.2949 | 39.8347 | 0.5097 | 28.7378 | 1.0372 |
| 20.3906 | 1.3377 | 11.9007 | 2.3174 | 39.3625 | 0.5250 | 28.1020 | 1.0850 |
| 19.7052 | 1.3970 | 11.7662 | 2.3391 | 38.9853 | 0.5378 | 27.6503 | 1.1190 |
| 19.1696 | 1.4411 | 11.4296 | 2.3976 | 38.5317 | 0.5501 | 27.0915 | 1.1573 |
| 18.6235 | 1.4903 | 11.0722 | 2.4621 | 37.9793 | 0.5782 | 26.6232 | 1.1912 |
| 18.0137 | 1.5493 | 10.7831 | 2.5186 | 37.5467 | 0.5901 | 25.9215 | 1.2543 |
| 17.4941 | 1.6020 | 10.5503 | 2.5653 | 37.1287 | 0.6046 | 25.3508 | 1.3027 |
| 17.0714 | 1.6442 | 10.3117 | 2.6129 | 36.7476 | 0.6150 | 24.7786 | 1.3507 |
| 16.7122 | 1.6794 | 9.5704 | 2.7733 | 36.2147 | 0.6408 | 24.3515 | 1.3933 |
| 16.2801 | 1.7278 | | | | | | |
| 15.9275 | 1.7663 | | | | | | |

**Table S17.** Experimental weight fraction data for $[C_2C_1Py][C_4F_9SO_3]$(1) + salt and carbohydrate (2) + $H_2O$(3) at 25 °C.

| $K_3$Citrate (cont.) | | D-glucose | | | | | |
|---|---|---|---|---|---|---|---|
| **100 w1** | **100 w2** | **100 w1** | **100 w2** | **100 w1** | **100 w2** | **100 w1** | **100 w2** |
| 24.0307 | 1.4177 | 72.3358 | 4.1621 | 34.5023 | 13.3212 | 18.7484 | 18.4672 |
| 23.6395 | 1.4516 | 69.5677 | 4.6194 | 33.3403 | 13.6202 | 18.1394 | 18.7137 |
| 23.1223 | 1.4965 | 67.2718 | 4.8876 | 32.5428 | 13.8366 | 17.5165 | 19.0007 |
| 22.6326 | 1.5502 | 65.3132 | 5.2518 | 31.8013 | 14.0663 | 17.0231 | 19.1659 |
| 22.1537 | 1.6020 | 63.4322 | 5.6236 | 31.0344 | 14.2839 | 16.4594 | 19.4499 |
| 21.6060 | 1.6569 | 61.1539 | 6.2014 | 30.2946 | 14.4903 | 15.8431 | 19.7321 |
| 20.8745 | 1.7428 | 59.4149 | 6.5206 | 29.5757 | 14.7411 | 15.3414 | 19.9443 |
| 20.4882 | 1.7891 | 57.3154 | 7.0156 | 28.7381 | 14.9802 | 14.8566 | 20.1994 |
| 19.8947 | 1.8582 | 55.6995 | 7.5059 | 27.9289 | 15.2225 | 14.3923 | 20.4205 |
| 19.3896 | 1.9192 | 54.3886 | 7.8126 | 27.2675 | 15.4613 | 13.8900 | 20.6521 |
| 18.6816 | 2.0026 | 52.5888 | 8.2431 | 26.5715 | 15.6449 | 13.3757 | 20.9298 |
| 17.7121 | 2.1424 | 51.1896 | 8.6951 | 25.8351 | 15.8962 | 12.6957 | 21.2551 |
| 17.0739 | 2.2342 | 49.5985 | 9.0740 | 25.1060 | 16.1547 | 11.9911 | 21.7114 |
| 15.8572 | 2.4236 | 47.8381 | 9.5977 | 24.4852 | 16.3446 | 11.2317 | 22.1672 |
| | | 46.4231 | 9.9222 | 23.7961 | 16.5994 | 10.5059 | 22.6205 |
| | | 44.9095 | 10.3604 | 23.1678 | 16.8009 | 9.8598 | 23.0740 |
| | | 43.4664 | 10.8094 | 22.5770 | 16.9892 | 9.4220 | 23.3572 |
| | | 41.7061 | 11.3337 | 22.0369 | 17.1542 | 8.8813 | 23.7839 |
| | | 40.2231 | 11.6694 | 21.4656 | 17.3829 | 8.2128 | 24.3022 |
| | | 38.4733 | 12.1962 | 20.7488 | 17.6340 | 7.8332 | 24.6705 |
| | | 37.1019 | 12.5781 | 20.1508 | 17.8809 | 7.3907 | 25.1384 |
| | | 35.7798 | 12.9487 | 19.4878 | 18.1122 | | |

**Table S18.** Experimental weight fraction data for [$C_2C_1$Py][$C_4F_9SO_3$] (1) + salt and carbohydrate (2) + $H_2O$ (3) at 25 °C (continuing).

| [$N_{1112(OH)}$][$H_2PO_4$] | | sucrose | | | | | |
|---|---|---|---|---|---|---|---|
| **100 w1** | **100 w2** | **100 w1** | **100 w2** | **100 w1** | **100 w2** | **100 w1** | **100 w2** |
| 48.2200 | 0.7662 | 59.5843 | 7.5286 | 29.1866 | 18.0462 | 16.6380 | 23.4559 |
| 47.2940 | 0.8011 | 58.0039 | 7.9735 | 28.1321 | 18.4684 | 16.1702 | 23.7060 |
| 46.2691 | 0.8292 | 56.5897 | 8.3954 | 27.6562 | 18.6111 | 15.7586 | 23.9028 |
| 45.3560 | 0.8697 | 54.8686 | 9.0365 | 27.0530 | 18.8820 | 15.3076 | 24.2345 |
| 44.5731 | 0.8979 | 52.9264 | 9.7432 | 26.3950 | 19.1096 | 14.7369 | 24.6313 |
| 43.7998 | 0.9270 | 51.2443 | 10.1424 | 25.6722 | 19.4256 | 14.3638 | 24.7870 |
| 43.0149 | 0.9584 | 48.9973 | 10.9228 | 25.1663 | 19.5991 | 13.9304 | 25.0142 |
| 41.8690 | 0.9986 | 48.0300 | 11.3349 | 24.3429 | 19.9205 | 13.5113 | 25.2257 |
| 40.8571 | 1.0474 | 46.8410 | 11.7671 | 23.7446 | 20.1581 | 13.1068 | 25.5647 |
| 39.6186 | 1.1076 | 45.7874 | 12.0632 | 23.2110 | 20.3817 | 12.7355 | 25.7488 |
| 38.3772 | 1.1705 | 44.5030 | 12.5643 | 22.9073 | 20.5325 | 12.2957 | 26.0542 |
| 37.2422 | 1.2296 | 42.8355 | 13.2093 | 22.4083 | 20.7358 | 11.9233 | 26.2941 |
| 36.4321 | 1.2770 | 41.3518 | 13.6480 | 21.9046 | 20.9532 | 11.7645 | 26.4338 |
| 35.2159 | 1.3526 | 39.9257 | 14.1898 | 21.5692 | 21.0754 | 11.2113 | 26.9278 |
| 33.5356 | 1.4641 | 39.0018 | 14.4752 | 21.1018 | 21.2770 | 10.6204 | 27.5583 |
| 32.1523 | 1.5654 | 37.5397 | 14.9942 | 20.6880 | 21.4386 | 10.0966 | 27.9372 |
| 30.6952 | 1.6828 | 36.4590 | 15.3059 | 20.2655 | 21.6591 | 9.2392 | 28.4343 |
| 28.9770 | 1.8380 | 35.2322 | 15.8600 | 19.8687 | 21.8461 | 8.6638 | 28.7878 |
| 27.6284 | 1.9715 | 34.7382 | 16.0454 | 19.5945 | 21.9680 | 8.1418 | 29.4978 |
| 25.6203 | 2.1934 | 33.9119 | 16.3313 | 19.1534 | 22.1691 | 7.7287 | 29.7425 |
| | | 32.8136 | 16.7653 | 18.7346 | 22.4496 | 7.3883 | 30.2072 |
| | | 31.7368 | 17.1832 | 18.1505 | 22.7076 | 6.9449 | 30.8214 |
| | | 30.9518 | 17.3696 | 17.6250 | 22.9362 | 6.4956 | 31.5100 |
| | | 30.0172 | 17.7104 | 17.1023 | 23.2087 | | |

**Table S19.** Experimental weight fraction data for $[N_{1112(OH)}]Cl$ (1) + salt (2) + $H_2O$ (3) at 25 °C.

| $K_3PO_4$ | | | | $K_2HPO_4$ | | | |
|---|---|---|---|---|---|---|---|
| **100 w1** | **100 w2** | **100 w1** | **100 w2** | **100 w1** | **100 w2** | **100 w1** | **100 w2** |
| 52.9119 | 4.9225 | 29.9999 | 16.6125 | 16.9473 | 31.3898 | 33.5110 | 15.6848 |
| 51.9041 | 5.1866 | 28.8514 | 17.3820 | 17.4535 | 30.7127 | 34.3513 | 14.9820 |
| 50.6307 | 5.6084 | 27.8491 | 18.1735 | 17.6752 | 30.4097 | 35.5284 | 14.1904 |
| 49.8301 | 5.8383 | 27.3485 | 18.6490 | 18.5056 | 29.6181 | 36.4818 | 13.5564 |
| 48.3981 | 6.3976 | 26.7466 | 19.0876 | 19.4098 | 28.7994 | 37.0210 | 13.1046 |
| 47.1306 | 6.8293 | 26.0811 | 19.5449 | 19.7108 | 28.2876 | 38.2874 | 12.3264 |
| 46.1309 | 7.2748 | 25.4084 | 20.0060 | 20.2213 | 27.6924 | 39.0102 | 11.8186 |
| 45.1638 | 7.6929 | 24.3875 | 20.8441 | 20.9383 | 26.9995 | 40.0343 | 11.2742 |
| 44.1246 | 8.1950 | 23.0683 | 21.8204 | 22.4198 | 25.6743 | 41.1526 | 10.5671 |
| 43.0908 | 8.7071 | 21.7196 | 22.8696 | 22.8313 | 25.1790 | 43.2143 | 9.3689 |
| 42.0780 | 9.1871 | 20.4302 | 23.9417 | 23.3715 | 24.6022 | 44.7333 | 8.6207 |
| 41.1850 | 9.6209 | 18.8356 | 25.2023 | 24.2880 | 23.7559 | 46.8797 | 7.7870 |
| 40.3771 | 10.0932 | 17.8460 | 26.0977 | 25.2628 | 22.8457 | 47.8480 | 7.3668 |
| 38.9789 | 10.8153 | 15.8885 | 27.7530 | 26.1816 | 22.0118 | 48.7169 | 6.9264 |
| 37.7687 | 11.5465 | 14.2686 | 29.0028 | 26.8262 | 21.3401 | 50.1469 | 6.3380 |
| 37.0059 | 12.0068 | 12.5870 | 30.3777 | 27.6569 | 20.5938 | 51.1365 | 5.9493 |
| 36.2484 | 12.5814 | 11.6598 | 31.3002 | 28.4353 | 19.9003 | 52.1718 | 5.5856 |
| 35.3874 | 13.1249 | 10.4596 | 32.5179 | 29.4190 | 19.0291 | 53.0523 | 5.2420 |
| 34.1945 | 13.7273 | 8.8588 | 33.8017 | 30.0881 | 18.2662 | 54.2844 | 4.9103 |
| 33.2952 | 14.3230 | 7.7642 | 35.0724 | 30.8720 | 17.6630 | | |
| 32.3465 | 14.8946 | 6.6287 | 36.5148 | 31.8915 | 16.8202 | | |
| 31.4618 | 15.5357 | 5.8572 | 37.5086 | 32.7093 | 16.2298 | | |

**Table S20.** Experimental weight fraction data for $[N_{1112(OH)}][CF_3SO_3]$(1) + salt (2) + $H_2O$ (3) at 25°C.

| $K_3PO_4$ | | | | $K_2HPO_4$ | | $K_3Citrate$ | |
|---|---|---|---|---|---|---|---|
| **100 w1** | **100 w2** | **100 w1** | **100 w2** | **100 w1** | **100 w2** | **100 w1** | **100 w2** |
| 62.9609 | 3.9033 | 13.8763 | 22.7615 | 63.5806 | 3.5574 | 55.4793 | 10.2390 |
| 53.3120 | 5.9101 | 13.0276 | 23.3592 | 58.8441 | 4.7846 | 51.2535 | 12.3148 |
| 47.4099 | 8.1823 | 12.1743 | 23.9924 | 54.6407 | 5.7235 | 47.5477 | 13.9839 |
| 44.0098 | 9.2503 | 11.6524 | 24.2574 | 49.7735 | 7.3686 | 43.2097 | 16.3416 |
| 40.6945 | 10.1776 | 10.8524 | 24.8148 | 44.2078 | 9.2749 | 39.6761 | 18.2293 |
| 38.5269 | 10.7258 | 10.1051 | 25.4410 | 40.0447 | 10.9647 | 36.2537 | 20.1060 |
| 36.4822 | 11.6112 | 9.4619 | 25.9319 | 36.6140 | 12.5193 | 34.9092 | 20.7886 |
| 34.6457 | 12.4542 | 8.8616 | 26.4403 | 33.5704 | 13.9230 | 33.4074 | 21.6332 |
| 32.1920 | 13.5221 | 8.1379 | 27.1258 | 31.0381 | 15.2709 | 30.9812 | 22.9985 |
| 30.0192 | 14.4792 | 7.3892 | 27.9733 | 29.3263 | 16.0830 | 29.5906 | 23.8592 |
| 28.1415 | 15.3511 | | | 27.8992 | 16.8089 | 28.3462 | 24.5966 |
| 26.4941 | 16.0607 | | | 25.8081 | 17.8859 | 27.3028 | 25.2474 |
| 25.3723 | 16.6319 | | | 24.5282 | 18.5639 | 26.1602 | 25.9582 |
| 24.3285 | 17.1311 | | | 22.7511 | 19.5615 | 24.4412 | 27.0962 |
| 23.3581 | 17.6317 | | | 21.3438 | 20.3458 | 23.3150 | 27.8905 |
| 22.2472 | 17.9723 | | | 20.0622 | 21.0971 | 22.2294 | 28.6553 |
| 21.4494 | 18.3736 | | | 18.6201 | 21.9852 | | |
| 20.6530 | 18.8225 | | | 17.3586 | 22.7892 | | |
| 19.9097 | 19.1873 | | | 15.9926 | 23.7097 | | |
| 19.0442 | 19.5418 | | | 14.8084 | 24.5812 | | |
| 18.1524 | 19.9300 | | | 13.4921 | 25.4516 | | |
| 17.0590 | 20.6610 | | | 12.6056 | 26.2366 | | |
| 15.9871 | 21.2819 | | | 11.7005 | 26.9721 | | |
| 14.9124 | 22.0000 | | | 10.5349 | 28.0283 | | |

**Table S21.** Experimental weight fraction data for $[N_{1112(OH)}][C_4F_9SO_3]$ and $[C_4C_1Im][CF_3SO_3]$ (1) + salt $[N_{1112(OH)}][H_2PO_4]$ (2) + $H_2O$ (3) at 25 °C.

| $[N_{1112(OH)}][C_4F_9SO_3]$ | | | | $[N_{1112(OH)}][H_2PO_4]$ | | | |
|---|---|---|---|---|---|---|---|
| **100 w1** | **100 w2** | **100 w1** | **100 w2** | **100 w1** | **100 w2** | **100 w1** | **100 w2** |
| 54.8872 | 14.5791 | 19.1061 | 33.4106 | 62.2263 | 6.4012 | 34.9019 | 15.2711 |
| 49.7528 | 17.0081 | 18.0377 | 34.0873 | 59.3938 | 7.2366 | 33.0835 | 15.9876 |
| 42.9443 | 20.3352 | 16.5712 | 35.0247 | 56.1494 | 7.9080 | 31.1697 | 16.9228 |
| 37.7050 | 23.1091 | 15.2406 | 35.9032 | 52.5370 | 8.9746 | 29.7810 | 17.5491 |
| 32.1217 | 26.1228 | 13.8883 | 36.8681 | 49.2556 | 10.0138 | 28.0254 | 18.5280 |
| 27.0911 | 28.8457 | 12.4932 | 37.9306 | 46.3900 | 10.9404 | 26.0145 | 19.6923 |
| 25.4181 | 29.7782 | 10.6497 | 39.4429 | 43.7295 | 11.8109 | 23.9068 | 21.1201 |
| 24.0434 | 30.5399 | | | 41.2732 | 12.7586 | 22.2976 | 22.3603 |
| 21.9049 | 31.7717 | | | 39.0804 | 13.4878 | 20.4727 | 23.9438 |
| 20.2362 | 32.7171 | | | 36.8798 | 14.4279 | 19.1818 | 25.1866 |

**Table S22.** Tie-line length and tie-line slope for every ternary mixture of the selected ABS BP at 25 °C.

| BP# | ABS Composition (%wt) | TLL | TL slope |
|---|---|---|---|
| BP#1 | 30% $[C_2C_1Im][CF_3SO_3]$ + 10% $K_3PO_4$ | 50.0766 | -3.2441 |
| BP#2 | 30% $[C_4C_1Im][CF_3SO_3]$ + 5% $K_3PO_4$ | 50.4683 | -8.4054 |
| BP#3 | 30% $[C_2C_1Im][C_4F_9SO_3]$ + 2% $K_3PO_4$ | 47.8966 | -11.8432 |
| BP#4 | 30% $[C_4C_1Im][CF_3SO_3]$ + 25% sucrose | 45.4241 | -1.8444 |
| BP#5 | 30% $[C_2C_1Im][C_4F_9SO_3]$ + 25% sucrose | 57.3121 | -2.0039 |
| BP#6 | 30% $[C_4C_1Im][CF_3SO_3]$ + 25% glucose | 66.2689 | -2.1517 |
| BP#7 | 30% $[C_2C_1Im][C_4F_9SO_3]$ + 25% glucose | 74.8624 | -2.2647 |
| BP#8 | 30% $[C_2C_1Im][C_4F_9SO_3]$ + 6% $[N_{1112(OH)}][H_2PO_4]$ | 52.6002 | -4.8715 |
| BP#9 | 30% $[C_2C_1Im][C_4F_9SO_3]$ + 10% $[N_{1112(OH)}][H_2PO_4]$ | 83.3946 | -2.8459 |
| BP#10 | 30% $[C_2C_1Im][C_4F_9SO_3]$ + 20% $[N_{1112(OH)}][H_2PO_4]$ | 68.3043 | -4.5550 |
| BP#11 | 30% $[C_4C_1Im][CF_3SO_3]$ + 20% $[N_{1112(OH)}][H_2PO_4]$ | 48.8872 | -2.5518 |
| BP#12 | 30% $[N_{1112(OH)}][C_4F_9SO_3]$ + 30% $[N_{1112(OH)}][H_2PO_4]$ | 65.5894 | -1.6632 |

**Table S23.** Critical Aggregation Concentration, CAC, of $[C_2C_1Im][C_4F_9SO_3]$and $[N_{1112(OH)}][C_4F_9SO_3]$in aqueous solution at 25 °C [1].

| | $[C_2C_1Im][C_4F_9SO_3]$(mM)$^{\dagger}$ | $[N_{1112(OH)}][C_4F_9SO_3]$(mM)$^{\ddagger}$ |
|---|---|---|
| 1$^{st}$ CAC | 14.40 | 16.02 |
| 2$^{nd}$ CAC | 34.48 | 35.17 |
| 3$^{rd}$ CAC | 76.54 | 185.65 |
| 4$^{th}$ CAC | 106.09 | n.d. |

$^{\dagger}$Determined by ITC; $^{\ddagger}$Determined by Conductometry.

**Table S24.** DSC melting temperature ($T_m$) and enthalpy change (ΔH) for 1.0 mg/mL of Lys in water and aqueous solutions of an increasing concentration of ABS phase-forming components.

| | $[C_2C_1Im][C_4F_9SO_3]$ | | $[C_4C_1Im][CF_3SO_3]$ | | $[N_{1112(OH)}][C_4F_9SO_3]$ | | $[N_{1112(OH)}][H_2PO_4]$ | |
|---|---|---|---|---|---|---|---|---|
| **Concentration (mM)** | **$T_m$ (°C)** | **ΔH (KJ/mol)** | **$T_m$ (°C)** | **ΔH (KJ/mol)** | **$T_m$ (°C)** | **ΔH (KJ/mol)** | **$T_m$ (°C)** | **ΔH (KJ/mol)** |
| 0 | 74.56 | 493.6 | 74.56 | 493.6 | 74.56 | 493.6 | 74.56 | 493.6 |
| 0.1 | 75.45 | 466.5 | 74.50 | 441.3 | 77.15 | 570.4 | 77.10 | 564.1 |
| 1 | 75.44 | 469.6 | 75.54 | 438.1 | 76.65 | 534.5 | 77.11 | 554.2 |
| 5 | 73.52 | 483.4 | 74.64 | 404.6 | 75.19 | 514.5 | 76.70 | 597.6 |
| 10 | 72.66 | 431.7 | 74.16 | 395.6 | 74.28 | 560.8 | 77.06 | 576.4 |
| 25 | 63.63 | 277.0 | - | - | 80.04;83.98;86.40 | 688.6;1120;2000 | - | - |
| 55 | - † | - | - | - | - † | - | - | - |
| 120 | - † | - | - | - | - | - | - | - |
| 200 | - | - | - | - | 60.15;65.13;67.62;69.26 | 611.8;848.2;1595;2000; | - | - |

† insufficient signal/non detectable

**Table S25.** Estimation of 1.0 mg/ml Lys secondary structure content from CD spectra deconvolution using K2D algorithm [2], at 25 °C.

| | $[N_{1112(OH)}][H_2PO_4]$ | | | $[N_{1112(OH)}][C_4F_9SO_3]$ | | | $[C_2C_1Im][C_4F_9SO_3]$ | | | $[C_4C_1Im][CF_3SO_3]$ | | |
|---|---|---|---|---|---|---|---|---|---|---|---|---|
| **Conc. (mM)** | $\alpha$-helix | $\beta$-sheet | Random coil | $\alpha$-helix | $\beta$-sheet | Random coil | $\alpha$-helix | $\beta$-sheet | Random coil | $\alpha$-helix | $\beta$-sheet | Random coil |
| 0 | 0.29 | 0.14 | 0.57 | 0.29 | 0.14 | 0.57 | 0.2 | 0.14 | 0.57 | 0.29 | 0.14 | 0.57 |
| 0.1 | 0.29 | 0.15 | 0.56 | 0.28 | 0.15 | 0.57 | 0.28 | 0.15 | 0.57 | 0.28 | 0.15 | 0.56 |
| 1 | 0.28 | 0.14 | 0.58 | 0.28 | 0.14 | 0.58 | 0.28 | 0.14 | 0.58 | 0.28 | 0.15 | 0.57 |
| 5 | 0.25 | 0.18 | 0.56 | 0.25 | 0.19 | 0.56 | 0.28 | 0.14 | 0.57 | 0.28 | 0.15 | 0.57 |
| 25 | 0.24 | 0.2 | 0.57 | 0.24 | 0.2 | 0.57 | 0.28 | 0.15 | 0.57 | 0.28 | 0.15 | 0.57 |
| 55 | -† | -† | -† | -† | -† | -† | 0.28 | 0.14 | 0.58 | 0.28 | 0.15 | 0.57 |
| 90 | -† | -† | -† | -† | -† | -† | 0.31 | 0.1 | 0.59 | 0.28 | 0.15 | 0.56 |
| 120 | -† | -† | -† | -† | -† | -† | -† | -† | -† | 0.28 | 0.15 | 0.56 |
| 200 | -† | -† | -† | -† | -† | -† | -† | -† | -† | 0.29 | 0.16 | 0.55 |

†Not able to predict secondary content.

**Table S26.** Lys extraction efficiency (%EE; Equation 6) determined for the studied biphasic systems. Protein concentration in both ABS phases were quantified using four complementary quantitative methods: UV-VIS, BCA, MICRO BCA and Bradford Coomassie protein assay. The %EE values are the results of at least 3 partition experiments.

| **BP#** | **ABS Composition (%wt)** | **%EE UV-VIS** | **%EE BCA** | **%EE $\mu$BCA** | **%EE Bradford** |
|---|---|---|---|---|---|
| BP#1 | 30% $[C_2C_1Im][CF_3SO_3]$ + 10% $K_3PO_4$ | 97.11 ± 4.86 | 77.43 ± 3.41 | n.a. | n.a. |
| BP#2 | 30% $[C_4C_1Im][CF_3SO_3]$ + 5% $K_3PO_4$ | 83.06 ± 0.17 | 56.52 ± 2.94 | n.a. | n.a. |
| BP#3 | 30% $[C_2C_1Im][C_4F_9SO_3]$ + 2% $K_3PO_4$ | 96.25 ± 4.81 | 91.55 ± 6.96 | n.a. | n.a. |
| BP#4 | 30% $[C_4C_1Im][CF_3SO_3]$ + 25% sucrose | 29.99 ± 1.62 | 15.69 ± 1.31 | n.a. | 10.12 ± 0.81 |
| BP#5 | 30% $[C_2C_1Im][C_4F_9SO_3]$ + 25% sucrose | 97.43 ± 4.87 | 98.74 ± 9.87 | n.a. | 85.15 ± 10.04 |
| BP#6 | 30% $[C_4C_1Im][CF_3SO_3]$ + 25% glucose | 19.22 ± 3.43 | 32.51 ± 0.33 | 34.51 ± 3.45 | 14.52 ± 3.92 |
| BP#7 | 30% $[C_2C_1Im][C_4F_9SO_3]$ + 25% glucose | 98.68 ± 4.93 | n.a. | n.a. | n.a. |
| BP#8 | 30% $[C_2C_1Im][C_4F_9SO_3]$ + 6% $[N_{1112(OH)}][H_2PO_4]$ | 98.35 ± 4.92 | 99.47 ± 9.95 | n.a. | n.a. |
| BP#9 | 30% $[C_2C_1Im][C_4F_9SO_3]$ + 10% $[N_{1112(OH)}][H_2PO_4]$ | 98.04 ± 4.90 | 93.48 ± 9.35 | n.a. | n.a. |
| BP#10 | 30% $[C_2C_1Im][C_4F_9SO_3]$ + 20% $[N_{1112(OH)}][H_2PO_4]$ | 20.82 ± 1.46 | 4.46 ± 0.27 | 8.14 ± 0.49 | n.a. |
| BP#11 | 30% $[C_4C_1Im][CF_3SO_3]$ + 20% $[N_{1112(OH)}][H_2PO_4]$ | 13.84 ± 0.39 | 0.78 ± 0.01 | n.a. | n.a. |
| BP#12 | 30% $[N_{1112(OH)}][C_4F_9SO_3]$ + 30% $[N_{1112(OH)}][H_2PO_4]$ | 74.77 ± 2.69 | 73.37 ± 8.28 | n.a. | 67.53 ± 4.05 |

**Table S27.** ABS phase-forming ionic liquid and non-ionic liquid composition (mM) of both ionic liquid-rich phase (FIL-rich phase and mere fluoro-containing IL-rich phase) and non-ionic liquid-rich phase ($K_3PO_4$-rich phase, sucrose-rich phase, glucose-rich phase, and $[N_{1112(OH)}][H_2PO_4]$-rich phase), at 25 °C.

| | | ionic liquid-rich phase (bottom phase) | | non-ionic liquid-rich phase (top phase) | |
|---|---|---|---|---|---|
| **BP#** | **ABS Composition (%wt)** | **IL (mM)** | **non-IL (mM)** | **IL (mM)** | **non-IL (mM)** |
| BP#1 | 30% $[C_2C_1Im][CF_3SO_3]$ + 10% $K_3PO_4$ | 5301 | 237 | 454 | 1075 |
| BP#2 | 30% $[C_4C_1Im][CF_3SO_3]$ + 5% $K_3PO_4$ | 6411 | 110 | 630 | 405 |
| BP#3 | 30% $[C_2C_1Im][C_4F_9SO_3]$ + 2% $K_3PO_4$ | 3007 | 36 | 116 | 358 |
| BP#4 | 30% $[C_4C_1Im][CF_3SO_3]$ + 25% sucrose | 6396 | 969 | 1299 | 1978 |
| BP#5 | 30% $[C_2C_1Im][C_4F_9SO_3]$ + 25% sucrose | 5884 | 658 | 705 | 1101 |
| BP#6 | 30% $[C_4C_1Im][CF_3SO_3]$ + 25% glucose | 10028 | 1068 | 777 | 3400 |
| BP#7 | 30% $[C_2C_1Im][C_4F_9SO_3]$ + 25% glucose | 9013 | 1122 | 318 | 3232 |
| BP#8 | 30% $[C_2C_1Im][C_4F_9SO_3]$ + 6% $[N_{1112(OH)}][H_2PO_4]$ | 3266 | 186 | 117 | 714 |
| BP#9 | 30% $[C_2C_1Im][C_4F_9SO_3]$ + 10% $[N_{1112(OH)}][H_2PO_4]$ | 5561 | 173 | 148 | 1679 |
| BP#10 | 30% $[C_2C_1Im][C_4F_9SO_3]$ + 20% $[N_{1112(OH)}][H_2PO_4]$ | 11456 | 195 | 184 | 2086 |
| BP#11 | 30% $[C_4C_1Im][CF_3SO_3]$ + 20% $[N_{1112(OH)}][H_2PO_4]$ | 8310 | 1027 | 1326 | 2131 |
| BP#12 | 30% $[N_{1112(OH)}][C_4F_9SO_3]$ + 30% $[N_{1112(OH)}][H_2PO_4]$ | 6237 | 1990 | 415 | 4758 |

**Table S28.** DSC melting temperature $T_m$ (°C) for 0.5 mg/ml, 1.0 mg/ml and 3.0 mg/mL Lys in water, ≈1.0 mg/ml lys resuspended in water (recover from $[C_2C_1Im][C_4F_9SO_3]$-rich phase of BP#5 (30% wt $[C_2C_1Im][C_4F_9SO_3]$ + 25% wt sucrose), BP#7 (30% wt $[C_2C_1Im][C_4F_9SO_3]$ + 25% wt glucose), and BP#8 (30% wt $[C_2C_1Im][C_4F_9SO_3]$ + 6% wt $[N_{1112(OH)}][H_2PO_4]$) in the partition of 1.0 mg/ml Lys, and resuspended in water), ≈1.0 mg/ml Lys in $[C_4C_1Im][CF_3SO_3]$-rich phase (partition of 1.0 mg/ml Lys in BP#4, 30% wt $[C_4C_1Im][CF_3SO_3]$ + 25%wt sucrose, and BP#6, 30% wt $[C_4C_1Im][CF_3SO_3]$ + 25%wt glucose), ≈4.0 mg/ml Lys in $[C_2C_1Im][C_4F_9SO_3]$-rich phase (partition of 1.0 mg/ml Lys in BP#5, 30% wt $[C_2C_1Im][C_4F_9SO_3]$ + 25%wt sucrose), ≈3.0 mg/ml Lys in $[C_2C_1Im][C_4F_9SO_3]$-rich phase (partition of 1.0 mg/ml Lys in BP#7, 30% wt $[C_2C_1Im][C_4F_9SO_3]$ + 25%wt glucose), ≈1.0 mg/ml Lys in $[N_{1112(OH)}][H_2PO_4]$-rich phase (partition of 1.0 mg/ml Lys in BP#10, 30% wt $[C_2C_1Im][C_4F_9SO_3]$ + 20%wt $[N_{1112(OH)}][H_2PO_4]$, and BP#11, 30% wt $[C_4C_1Im][CF_3SO_3]$ + 20%wt $[N_{1112(OH)}][H_2PO_4]$) and ≈0.5 mg/ml Lys in $[N_{1112(OH)}][H_2PO_4]$-rich phase (partition of 1.0 mg/ml Lys in BP#12, 30% wt $[N_{1112(OH)}][C_4F_9SO_3]$ + 30%wt $[N_{1112(OH)}][H_2PO_4]$).

| | |
|---|---|
| **Lys 3.0 mg/ml** | |
| Water | 73.85±0.005 |
| $[C_2C_1Im][C_4F_9SO_3]$-rp (BP#5 and BP#7) | 39.95±0.010 (BP#5) and 41.30±0.008 (BP#7) |
| **Lys 1.0 mg/ml** | |
| Water | 74.56±0.835 |
| $[C_4C_1Im][CF_3SO_3]$-rp (BP#4 and BP#6) | 37.81±0.011 (BP#4) and 36.09±0.011 (BP#6) |
| $[N_{1112(OH)}][H_2PO_4]$-rp (BP#10 and BP#11) | 77.11±0.007 (BP#10) and 54.89±0.012 (BP#11) |
| resuspended in Water (BP#5, BP#7 and BP#8) (Lys recover from $[C_2C_1Im][C_4F_9SO_3]$-rp) | 69.04±0.005 (BP#5), 68.43±0.004 (BP#7) and 69.15±0.013 (BP#8) |
| **Lys 0.5 mg/ml** | |
| Water | 69.26±0.031 |
| $[N_{1112(OH)}][H_2PO_4]$-rp (BP#12) | 66.70±0.008 |

**Table S29.** Discriminated identification of the samples loaded in each of the lanes corresponding to the gels displayed in Figure 17 (C and D monochromatic version of A and B, respectively).

| SDS-PAGE | lane identification |
|---|---|
| Figure 17 A and C BP#3 | lane 1, protein ladder; lane 2, Lys+BSA standard solution; lane 3, empty lane;<br>lane 4, $K_3PO_4$-rp individual Lys partition‡; lane 5, $K_3PO_4$-rp individual Lys partition†;<br>lane 6, $[C_2C_1Im][C_4F_9SO_3]$-rp individual Lys partition; lane 7, $K_3PO_4$-rp individual BSA partition;<br>lane 8, $[C_2C_1Im][C_4F_9SO_3]$-rp individual BSA partition†;<br>lane 9, $K_3PO_4$-rp Lys+BSA partition;<br>lane 10, $[C_2C_1Im][C_4F_9SO_3]$-rp Lys+BSA partition;<br>lane 11, empty lane; lane 12, $K_3PO_4$-rp Lys+BSA partition‡. |
| Figure 17 B and D BP#5 | lane 1, protein ladder; lane 2, empty lane; lane 3, Lys+BSA standard solution;<br>lane 4, empty lane; lane 5, $[C_2C_1Im][C_4F_9SO_3]$-rp Lys partition‡;<br>lane 6, Sucrose-rp individual Lys partition; lane 7, empty lane;<br>lane 8, $[C_2C_1Im][C_4F_9SO_3]$-rp individual BSA partition;<br>lane 9, Sucrose-rp individual BSA partition‡;<br>lane 10, empty lane;<br>lane 11, $[C_2C_1Im][C_4F_9SO_3]$-rp individual Lys+BSA partition; lane 12, Sucrose-rp individual Lys+BSA partition. |

†amount of protein lower than 0.5 $\mu$g, due to the used dilution factor equal to the counterpart ABS phase (0.5 $\mu$g of protein). counterpart ABS phase in adjacent lane, left or right.
‡amount of protein lower than 15 ng (determined detection limit), thus sampling without dilution. correspondent counterparts maintaining sampling without dilution (> 0.5 $\mu$g).

## References


1. Pereiro, A.B.; Araújo, J.M.; Teixeira, F.S.; Marrucho, I.M.; Piñeiro, M.M.; Rebelo, L.P.N. Aggregation behavior and total miscibility of fluorinated ionic liquids in water. *Langmuir* **2015**, *31*, 1283–1295. https://doi.org/10.1021/la503961h.
2. Andrade, M.A.; Chactfn, P.; Merelo, J.J.; Mordn, F. Evaluation of secondary structure of proteins from UV circular dichroism spectra using an unsupervised learning neural network, 1993.


**Disclaimer/Publisher's Note:** The statements, opinions and data contained in all publications are solely those of the individual author(s) and contributor(s) and not of MDPI and/or the editor(s). MDPI and/or the editor(s) disclaim responsibility for any injury to people or property resulting from any ideas, methods, instructions or products referred to in the content.
